# Integrated microbiomics and metabolomics analysis reveals distinct profiles in carbapenem-resistant *Acinetobacter baumannii* and *Escherichia coli* infections in Pancreatitis-associated sepsis

**DOI:** 10.1371/journal.pone.0340895

**Published:** 2026-02-10

**Authors:** Kongfan Zhu, Hua Hu, Zhijian Yang, Zhongchao Zhu

**Affiliations:** 1 Department of Pancreatic surgery, Renmin Hospital of Wuhan University, Wuhan, China; 2 Department of Bone and Joint Surgery, Renmin Hospital of Wuhan University, Wuhan, China; Yenepoya University, INDIA

## Abstract

**Background:**

Pancreatitis-associated sepsis (PAS) caused by carbapenem-resistant bacteria poses significant clinical challenges. The objective of this research was to examine the microbial and metabolic profiles of individuals with carbapenem-resistant *Acinetobacter baumannii* (CRAB) and *Escherichia coli* (CREC) infections using integrated microbiomics and metabolomics approaches.

**Methods:**

Peripheral blood samples from 11 PAS patients (8 CRAB, 3 CREC) were analyzed using 16S rDNA gene sequencing and untargeted metabolomics via LC-MS. Microbial diversity, community structure, and differential metabolites were examined between CRAB and CREC groups.

**Results:**

CRAB patients exhibited higher microbial diversity compared to CREC patients. p-*Proteobacteria*, p-*Firmicutes*, and p-*Cyanobacteria* predominated in both patient groups. Significant differences in microbial composition were observed, with p-*Proteobacteria* more abundant in CRAB and p-*Cyanobacteria* in CREC samples. g-*Enhydrobacter* and s-*Moraxella osloensis* were the biomarkers, significantly higher in CREC patients. Metabolomic analysis revealed 328 differential metabolites between groups, with the majority being downregulated in CRAB. The main categories of identified differential metabolites were amino acids and their derivatives. These differential metabolites were closely related to various metabolic pathways. The most significant metabolic difference between the two patient groups was the level of triglycerides. R-2 Methanandamide and 13-(β-D-glucosyloxy) docosanoic acid showed the highest correlation with g-*Enhydrobacter* and s-*Moraxella osloensis*.

**Conclusion:**

In PAS patients, s-*Moraxella osloensis* is a biomarker distinguishing CRAB and CREC infections, correlating with R-2 Methanandamide and 13-(β-D-glucosyloxy) docosanoic acid.

## 1. Introduction

Pancreatitis represents a complex inflammatory condition affecting the digestive system, marked by acute inflammatory responses and significant systemic complications. Its incidence is increasing globally [1,2]. Due to pancreatic function impairment, patients are prone to developing severe pancreatitis, which may ultimately lead to sepsis, PAS frequently develops under these circumstances [3]. Sepsis, a systemic inflammatory response caused by infection, can result in organ dysfunction and is a leading cause of mortality in intensive care units [4]. PAS significantly increases the mortality rate of pancreatitis patients, particularly when infected with drug-resistant bacteria, posing substantial challenges for clinical management. In recent years, the emergence of antibiotic-resistant bacteria such as CREC and CRAB has further exacerbated the treatment difficulties for PAS patients. These superbugs have developed resistance to multiple antibiotics, posing a severe threat to patients’ lives [5]. A review of the current literature reveals a paucity of studies specifically investigating the comparative roles of CREC and CRAB in the pathogenesis, diagnosis, or management paradigms within the distinct context of established PAS. While patients with MDRO infections in infected pancreatic necrosis are known to have higher mortality [6–8], and CRAB and CREC are independently recognized as significant pathogens imposing substantial clinical burdens [9,10], a direct comparison of their impact within the PAS cohort is lacking. Evidence from high-risk settings, such as ICUs, confirms the emergence of high-risk CREC and CRAB strains, with the COVID-19 pandemic further accelerating CRAB spread [11–13].

Current diagnostics mainly rely on microbial culture and susceptibility testing, which are time-consuming and may delay treatment [14]. International guidelines emphasize early, aggressive management [15], but for resistant strains, traditional empirical antibiotics often fail. This diagnostic delay underscores an urgent clinical need. Extrapolating from existing evidence, the early identification of patients infected with these specific pathogens is critical for facilitating the timely adjustment of therapeutic strategies and improving prognosis in PAS.

To address this challenge, researchers are exploring new diagnostic and therapeutic strategies, including genomic sequencing and metabolomics. Genomic sequencing offers rapid, culture-independent pathogen identification [16,17], though it faces challenges such as contamination from host DNA [18,19]. Metabolomics analysis via LC-MS provides a functional readout of the host’s physiological state, offering new insights into the metabolic perturbations caused by specific infections [20–22]. However, comparative studies focusing specifically on sepsis induced by CREC versus CRAB remain notably scarce [23,24]. Significant knowledge gaps persist regarding differential risk factors, clinical prognoses, and optimal therapeutic strategies for infections caused by these distinct pathogens, particularly after the development of PAS [25,26].

To address this critical knowledge gap, we initiated this investigation by collecting peripheral blood samples from patients diagnosed with PAS and infected with either CRAB or CREC. We employed an integrated multi-omics strategy, hypothesizing that this approach could reveal distinct host-pathogen interaction profiles and provide novel biomarkers. We utilized 16S rDNA gene sequencing to characterize and compare the microbial community structures, identifying differential taxa and potential biomarkers using LEfSe analysis. Concurrently, untargeted LC-MS-based metabolomics was used to profile metabolic alterations, identifying differential metabolites and perturbed pathways. Finally, we performed correlation analyses to investigate the interrelationships between microbial biomarkers and differential metabolites. This study aims to move beyond single-level descriptions to explore the complex interplay between the host microbiome and metabolome in response to CRAB versus CREC infection during PAS. We hypothesize that this integrative analysis may reveal pathogen-specific signatures that could serve as a reference point for rapid diagnostics, aiding clinicians in differentiating these critical infections hours or days before culture results become available. Such timely identification is paramount for optimizing antimicrobial therapy, advancing precision medicine for PAS, and potentially illuminating novel therapeutic targets related to host metabolic responses.

## 2. Materials and methods

### 2.1. Patients

This single-centre observational investigation enrolled 11 patients with PAS (CRAB n = 8; CREC n = 3). The research received approval from the Clinical Research Ethics Committee, Renmin Hospital of Wuhan University (Ethics approval number: 2024K-K304(C01)). Sample collection for this study was conducted from [Dec 25, 2024] to [Mar 20, 2025]. Recruitment lasted for 146 days. The recruitment method involved collecting blood samples from patients with severe pancreatitis complicated by intra-abdominal infection. Peripheral blood reflects systemic microbial and metabolic changes associated with PAS and is readily accessible clinically. Therefore, peripheral blood samples were collected from all patients for 16S rDNA sequencing and untargeted metabolomics analysis. Baseline demographic and clinical characteristics of the patients are provided in S1 Table. All patients received standardized treatment for severe pancreatitis, which included the STEP-UP (Step-up Approach) strategy for peripancreatic necrotic tissue debridement. All patients underwent varying degrees of abdominal paracentesis and video-assisted pancreatic necrosectomy, as well as combined drug therapy with specialized antibiotics such as cefoperazone-tazobactam, tigecycline, and polymyxin. The Minimum Inhibitory Concentration (MIC) values for all patients are presented in S2 Table. MIC values were used to determine the resistance of 17 antibiotics. This research followed the guidelines set forth in the Declaration of Helsinki, and all participants provided their informed consent before enrollment. Inclusion Criteria: (1) Diagnostic Criteria: Patients meeting the Atlanta criteria for severe acute pancreatitis and with confirmed intra-abdominal infection via paracentesis drainage. (2) Infecting Pathogen: Blood or pus culture results indicating infection with CREC or CRAB. (3) Informed Consent: Patients or their legal representatives willing to participate in the study and having signed an informed consent form. Exclusion Criteria: (1) Non-severe Pancreatitis: Patients not meeting the Atlanta criteria for severe acute pancreatitis. (2) No Intra-abdominal Infection: Patients without confirmed intra-abdominal infection via paracentesis drainage. (3) Non-CREC/CRAB Infection: Patients whose blood or pus culture results do not indicate CREC or CRAB infection. (4) Immunosuppressed Individuals: Patients receiving immunosuppressive therapy, organ transplant recipients, or those currently undergoing chemotherapy, as this may affect the assessment of infection characteristics. (5) Pregnant or Breastfeeding Women: Pregnant or breastfeeding women are excluded to ensure the safety of the study participants. (6) Severe Comorbidities: Patients with severe liver or kidney dysfunction, terminal illness, or other conditions that may confound the study results. (7) Inability to Provide Valid Informed Consent: Patients unable to provide informed consent due to mental disorders or other reasons.

### 2.2. 16S rDNA sequencing

Genomic DNA was isolated from peripheral blood samples utilizing the QIAamp Fast DNA Stool Mini Kit (Cat. No. 51604, Qiagen, USA) following the guidelines provided by the manufacturer [27]. The negative control and samples were assessed for contamination by agarose gel electrophoresis during the amplification stage (S1 Fig). Amplicons targeting the bacterial V4 region were amplified with primers 515F and 806R purchased from Thermo Fisher Scientific. PCR utilized Phusion® High-Fidelity PCR Master Mix with GC Buffer (New England Biolabs) under the cycling conditions recommended by Earth Microbiome Project protocols. Amplicons were pooled, purified with AMPure XP beads, and sequenced on an Illumina NovaSeq 6000 platform (2 × 250 bp). Representative sequences were generated through read assembly, filtering, clustering, or denoising methods. OTU abundance statistics were calculated using filtered tags. Sequence information was compared against databases for species annotation and abundance analysis.

The top 10 organisms with the greatest prevalence at each taxonomic rank (Phylum, Class, Order, Family, Genus, Species) were selected for each specimen or group to create stacked bar charts depicting relative organism abundance. The top 35 microbial taxa, ranked by the total of quantitative measurements across all samples, were chosen. Heatmaps were constructed by organizing both species and sample levels according to their quantitative data in each sample. Analyses of alpha and beta diversity were utilized to clarify differences in species composition inside samples and community structure among samples, respectively. NMDS and weighted UniFrac PCoA were utilized to reflect inter- and intra-group differences among samples. LEfSe analysis was conducted to identify differential biomarkers between CRAB and CREC patients.

#### 2.2.1. The criteria for filtering and clustering reads.

Raw sequencing data were demultiplexed according to barcode sequences and PCR amplification primers to assign reads to their respective samples. Barcode and primer sequences were subsequently trimmed. Raw reads were processed using fastp (v0.22.0, https://github.com/OpenGene/fastp) to obtain high-quality reads by applying the following filtering criteria: automatic detection and removal of adapter sequences; removal of reads containing one or more N bases; removal of reads where over 40% of bases had a quality score < 15; trimming based on a 4-base sliding window where reads were truncated if the average quality within the window dropped below 20; removal of terminal polyG sequences; and discarding reads shorter than 150 bp. The resulting high-quality paired-end reads were merged using FLASH (v1.2.11, http://ccb.jhu.edu/software/FLASH/) to generate high-quality tags (Clean Tags). These Clean Tags were then aligned against a species annotation database using vsearch (v2.22.1, https://github.com/torognes/vsearch/) to detect chimeric sequences. Identified chimeric sequences were removed, yielding the final dataset of effective tags.

Effective Tags from all samples were clustered into OTUs using the Uparse algorithm (USEARCH v7, http://www.drive5.com/uparse/) with a default 97% sequence identity threshold. Based on the algorithm’s principles, the most abundant sequence within each cluster was selected as the representative sequence for that OTU. Single OTU in all samples tags sum’s lowest value, defined as all OTUs after flattening tags total number’s 0.005% [28]. Taxonomic assignment for OTU representative sequences was performed using the Mothur software (v1.48) against the SILVA SSUrRNA database (v138.1, http://www.arb-silva.de/) with a confidence threshold range of 0.8–1.0 [29]. This yielded taxonomic information, and the community composition for each sample was summarized at various taxonomic levels. Multiple sequence alignment of all representative OTU sequences was performed using MAFFT (v7.520, https://mafft.cbrc.jp/alignment/software/) to establish phylogenetic relationships. Finally, the OTU abundance data were normalized by rarefying each sample to the lowest sequencing depth present across the dataset. Subsequent diversity analyses were based on these rarefied data.

### 2.3. Untargeted metabolomics [30–32]

Serum aliquots stored at −80 °C were thawed on ice, vortexed for 10 s, and combined with 300 μL extraction solvent (acetonitrile: methanol 1:4, v/v) containing isotopically labelled internal standards (SCIEX). After vortexing for 3 min, samples were centrifuged at 12,000 × g (4 °C, 10 min); 200 μL supernatant was precipitated at −20 °C (30 min) and recentrifuged, and 180 μL was transferred to autosampler vials. Pooled QC samples, generated by combining equal aliquots from every extract, were injected after system conditioning and then after every tenth analytical sample; solvent blanks bracketed each batch to monitor carryover. Chromatography employed a Waters ACQUITY Premier HSS T3 column (1.8 μm, 2.1 × 100 mm) held at 40 °C with the gradient 0–2 min 5–20% B, 2–5 min 20–60% B, 5–6 min 60–99% B, 6–7.5 min hold 99% B, 7.5–7.6 min return to 5% B, 7.6–10 min re-equilibrate (flow 0.4 mL·min ⁻ ¹, injection 4 μL). Mass spectrometry was conducted on an AB SCIEX TripleTOF 6600 + instrument operated in positive and negative electrospray ionisation modes under information-dependent acquisition (IDA) using Analyst TF 1.7.1. Source parameters were: ion spray voltage +5000 V (positive) and −4000 V (negative); source gas 1 = 50 psi; source gas 2 = 60 psi; curtain gas = 35 psi; source temperature 550 °C (positive) and 450 °C (negative); declustering potential ±60 V. TOF MS scans covered m/z 50–1000 (200 ms accumulation), while product ion scans covered m/z 25–1000 (40 ms accumulation) with collision energy ±30 V and collision energy spread 15 V.

Raw data were converted to mzXML via ProteoWizard. Peak detection, alignment, and retention-time correction were performed in XCMS with centWave parameters. Features with >50% missing values per group were removed; intensity-dependent signal drift was corrected using QC-based support vector regression (SVR). Remaining missing values were imputed using k-nearest neighbour (k = 5). Features detected in solvent blanks were removed unless the sample-to-blank ratio exceeded 5:1. Positive and negative mode data were merged by retaining the highest MSI identification level and lowest QC coefficient of variation per compound. Intensities were log₂ transformed and unit-variance scaled prior to PCA and OPLS-DA. In order to avoid overfitting, a permutation test (200 permutations) was performed (S2 Fig).

Metabolite identification relied on an in-house spectral library augmented with KEGG, HMDB, METLIN, and metDNA resources. MSI levels were assigned as: Level 1a (reference standard matching for retention time, MS1, and MS/MS), Level 1b (reference standard matching with minor MS/MS deviations), Level 2 (library MS/MS match with accurate mass and retention time), Level 3 (putative class annotation), and Level 4 (accurate mass only; excluded from differential analysis). Differential metabolites were defined by VIP > 1 and Student’s t-test *P <* 0.05, with MSI Levels 1a–4 annotated.

### 2.4. Spearman statistical analysis

Unsupervised PCA was performed by statistics function prcomp within R (www.r-project.org, Version: 4.1.2). Correlation data were filtered according to a correlation coefficient |r| ≥ 0.9 and a significance test *P* *<* 0.05. All plots were generated using the ggplot2 package in R.

## 3. Results

### 3.1. Microbial diversity analysis between CRAB and CREC groups

**Fig 1:**
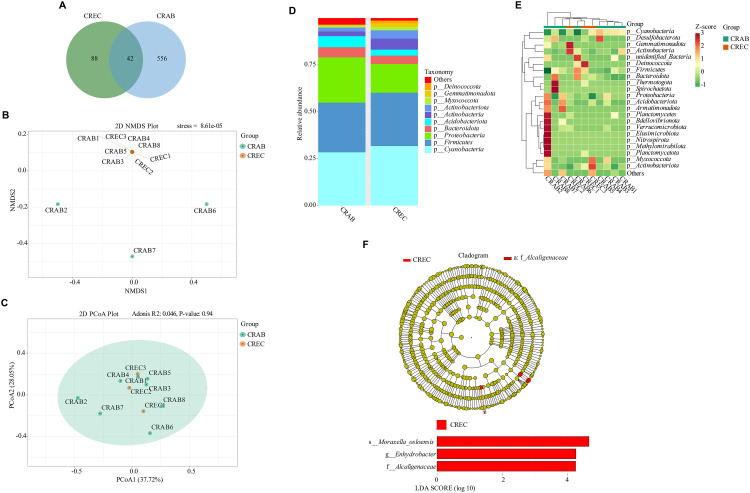
Diversity analysis of microbiota between *Carbapenem-resistant Acinetobacter baumannii* (CRAB) and *Carbapenem-resistant Escherichia coli* (CREC) groups. (A) Venn diagram of Amplicon Sequence Variants (ASVs) among the two groups. (B, C) Non-Metric Multi-Dimensional Scaling (NMDS) and weighted Unweighted UniFrac (UniFrac) Principal Coordinate Analysis (PCoA) conducted on the Operational Taxonomic Units (OTUs) of the microbiota from the CREC and CRAB groups. (D) Relative phylum abundance of ASVs among the CREC and CRAB groups. (E) Cluster analysis of the top 35 phylum abundances among the CREC and CRAB groups. (F) Cladogram generated from Linear Discriminant Analysis Effect Size (LEfSe) analysis showing the association between taxa (the levels represent, from inner to outer rings, phylum, class, order, family, and genus). The thresholds were set at a Linear Discriminant Analysis (LDA) score >4 and *P <* 0.05, with a focus on family, genus, and species level biomarkers. n = 8 in the CRAB group, and n = 3 in the CREC group.

High-throughput sequencing revealed distinct microbial community compositions in the CRAB and CREC groups. The CRAB group exhibited significantly higher microbial diversity, with 556 unique OTUs compared to 88 in the CREC group, while 42 OTUs were shared between groups (Fig 1A). Despite observable differences in overall community structure (Fig 1B and 1C), alpha diversity analyses indicated that there were no notable alterations in diversity and richness indices (S3 Fig). At the taxonomic rank of phylum, *Proteobacteria*, *Firmicutes*, and *Cyanobacteria* were the most abundant in both groups, collectively accounting for over 75% of total abundance (Fig 1D). Notably, the CREC group displayed a higher proportion of *Cyanobacteria* and lower *Proteobacteria* compared to the CRAB group. Heatmap analysis of the top 35 phyla further illustrated these compositional differences (Fig 1E). LEfSe identified *g-Enhydrobacter* and *s*-*Moraxella osloensis* as biomarkers in the CREC group (Fig 1F, S3 Table). Significant differences at the genus level for *Enhydrobacter* and species level for *Moraxella osloensis* were observed in three CRAB and three CREC patients (S4 Fig).

### 3.2. Differential metabolite analysis between CRAB and CREC groups

**Fig 2:**
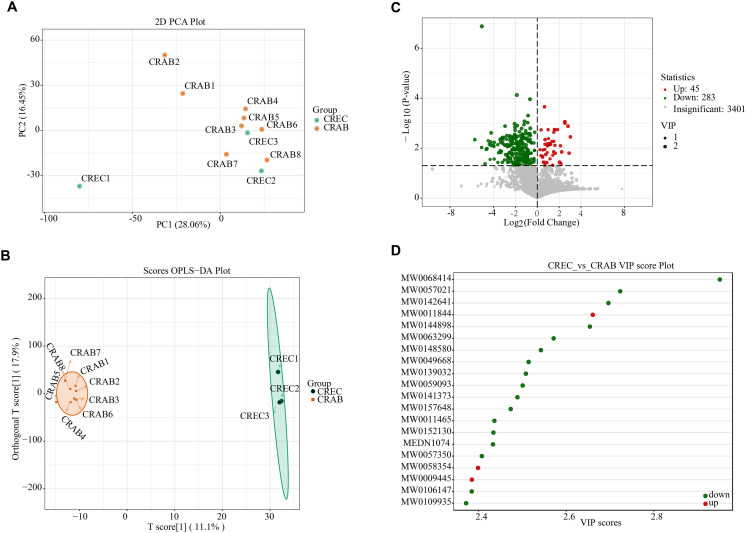
Differential metabolite analysis between *Carbapenem-resistant Acinetobacter baumannii* (CRAB) and *Carbapenem-resistant Escherichia coli* (CREC) groups. (A) 2D Principal Component Analysis (PCA) plot illustrating overall differences in metabolite composition between CRAB (red) and CREC (green) samples. (B) Orthogonal Partial Least Squares Discriminant Analysis (OPLS-DA) score plot. (C) Volcano plot of differential metabolites, with the x-axis representing log2 fold change in metabolite expression and the y-axis representing statistical significance (-log10 (p)). (D) Variable Importance in Projection (VIP) score plot displaying metabolites with the highest VIP values in the OPLS-DA model based on screening criteria for differential metabolites identified in group comparisons. n = 8 in the CRAB group, and n = 3 in the CREC group.

**Fig 3:**
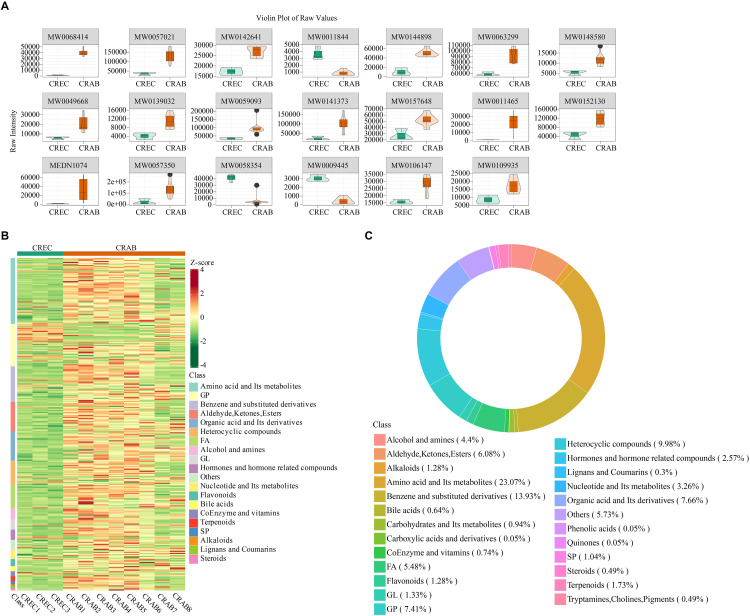
Differential metabolite enrichment analysis between *Carbapenem-resistant Acinetobacter baumannii* (CRAB) and *Carbapenem-resistant Escherichia coli* (CREC) groups. (A) Violin plot of the top 20 differential metabolites. (B) Enrichment analysis. (C) Differential metabolite classification. n = 8 in the CRAB group, and n = 3 in the CREC group.

Sample quality control was performed by analyzing the total ion current (TIC) chromatograms, the peak profiles of internal standards in blank samples, the Pearson correlation analysis of QC samples, and the distribution of the coefficient of variation (CV) for internal standard responses. The results showed that the instrument was stable, the data were reliable, and the repeatability was good (S5 Fig, S4 Table). PCA and OPLS-DA demonstrated clear separation between CRAB and CREC samples, indicating significant metabolic profile differences (Fig 2A and 2B). The CRAB group showed more dispersed sample distribution, suggesting greater heterogeneity. Differential metabolite analysis identified 283 downregulated and 45 upregulated metabolites in the CRAB group compared to CREC (Fig 2C). The metabolite with the highest VIP score was TG (14:1(9Z)/18:4(6Z,9Z,12Z,15Z)/20:5(5Z,8Z,11Z,14Z,17Z)) (Figs 2D and 3A). The leading 20 metabolites exhibiting the greatest disparity in concentration between the two cohorts are depicted in Fig 3A and Table 1. Enrichment analysis revealed that amino acids and their metabolites were the most abundant type of differential metabolites, accounting for 23.07% of the differences between groups (Fig 3B and 3C).

**Table 1:** The top 20 differential metabolites.

Compounds	Class I	Class II	Formula	Level	score	CAS	CREC1	CREC2	CREC3	CRAB1	CRAB2	CRAB3	CRAB4	CRAB5	CRAB6	CRAB7	CRAB8	VIP	P-value	FDR	Fold_Change	Type
TG(14:1(9Z)/18:4(6Z,9Z,12Z,15Z)/20:5(5Z,8Z,11Z,14Z,17Z))	GL	TG	C55H86O6	3	0.8475	–	684.57	752.81	2089.7	36217.16	36379.97	36493.73	33282.21	39148.64	44187.25	51351.1	42088.17	2.950705	1.32E-07	0.000491	0.029471	down
1-(9Z-octadecenoyl)-2-(9Z,12Z-octadecadienoyl)-sn-glycero-3-phosphocholine	GP	PC	C44H82NO8P	3	0.512	17041-44-0	39919.2	26140.72	41806.43	76007.74	151741.1	94718.46	182173.5	106550.5	156569	143704.9	146975.1	2.723167	7.43E-05	0.135956	0.271762	down
6-Oxoestradiol diacetate	Aldehyde,Ketones,Esters	Esters	C22H26O5	3	0.6284	3434-45-5	19315.11	17206.28	15285.49	26958.01	29580.44	29607.02	23394.55	24153.26	28730.86	28833.66	25173.42	2.69627	0.001601	0.237992	0.638317	down
Tacalcitol	CoEnzyme and vitamins	CoEnzyme and vitamins	C27H44O3	3	0.7912	57333-96-7	4801.79	3286.74	2881.56	634.4	616.18	1164.36	484.91	967.97	985.44	691.95	1549.81	2.660706	0.036677	0.472087	4.123114	up
Undecaprenyl phosphate	Organic acid and Its derivatives	Phosphoric acids	C55H91O4P	3	0.7125	25126-51-6	2675.8	8659.46	19241.24	46139.79	46255.41	46400.06	42316.76	49775.65	56182.03	65290.5	53513.12	2.653955	0.003909	0.237992	0.200894	down
R-2 Methanandamide	FA	Oxidized lipids	C23H39NO2	2	0.9334	157182-47-3	57490.15	56247.71	62855.4	108523.9	101202.7	99790.66	99217.44	107898.1	77068.57	78679.45	77379.47	2.571241	0.000109	0.135956	0.628088	down
Diospyrin	Benzene and substituted derivatives	Benzene and substituted derivatives	C22H14O6	3	0.6023	28164-57-0	3839.26	6173.99	5890.28	12201.38	9073.8	13454.32	18453.03	12580.06	8185.49	11872.41	10116.73	2.542104	0.000842	0.237992	0.442054	down
1-Pentadecanoyl-3-osbondoyl-sn-glycerol	GL	DG	C40H68O5	3	0.5761	–	6524.33	3883.63	6859.39	14837.09	26566.48	34653.85	28751.65	15337.42	17918.01	11390.85	15150.26	2.514069	0.001245	0.237992	0.279737	down
Myricanol 5-beta-sophoroside	Benzene and substituted derivatives	Benzene and substituted derivatives	C33H46O15	3	0.5074	116107-17-6	5527.96	4301.36	2364.09	7017.51	7895.45	9268.89	13168.47	8679.14	12373.04	15940.02	13721.86	2.507905	0.001637	0.237992	0.369227	down
1-(1Z-octadecenyl)-2-(9Z-octadecenoyl)-sn-glycero-3-phosphoethanolamine	GP	PE-P	C41H80NO7P	3	0.6185	144371-68-6	34603.98	26654.97	42095.13	86861.54	205602.9	80469.73	102065.2	85913.89	90469.58	99679.61	60589.47	2.500538	0.003233	0.237992	0.339568	down
13-(beta-D-glucosyloxy) docosanoic acid	FA	FFA	C28H54O8	3	0.5448	–	19774.8	16683.47	35898.31	121282.3	113591.2	96895.04	127233.9	172369.7	96551.18	48009.9	42084.6	2.488622	0.001051	0.237992	0.235876	down
Thr-Gly-Ile-Phe-Thr	Amino acid and Its metabolites	Small Peptide	C25H39N5O8	3	0.5971	–	20117.26	23325	38384.24	67793.38	47917.67	48998.35	58936.73	51661.25	56881.33	49907.81	37033.56	2.473238	0.024192	0.395672	0.520612	down
(8’R)-Neochrome	Others	Others	C41H58O3	3	0.6932	59491-56-4	1053.39	1239.54	1224.83	37511.64	22643.03	34136.81	28940.35	26241.86	18907.22	5256.8	2162.36	2.436061	0.00244	0.237992	0.05336	down
Pretyrosine	Organic acid and Its derivatives	Organic acid and Its derivatives	C10H13NO5	3	0.6653	–	24738.25	58891.31	53905.3	133502	106424.4	154103.2	134234.7	96408.79	155058.3	82345.09	80731.8	2.433708	0.002766	0.237992	0.389008	down
Allocholic acid	Bile acids	Bile acids	C24H40O5	2	0.9359	2464-18-8	714.45	903.27	2142.45	2390.05	20104.02	11144.36	33125.41	66870.99	55734.42	7863.69	57514.74	2.43277	0.01104	0.275335	0.039361	down
PC(20:4(5Z,8Z,11Z,14Z)/18:1(11Z))	GP	PC	C46H82NO8P	3	0.5137	–	1822.48	7118.11	50516.68	266934.8	201153.5	115203.4	148888	103861.2	139733.6	76718.2	45608.78	2.407373	0.003173	0.237992	0.144388	down
PE(18:3(9Z,12Z,15Z)/18:1(9Z))	GP	PE	C41H74NO8P	3	0.7531	–	43261.15	33682.31	46299.54	3879.4	4691.57	4015.58	6100.57	4180.74	1268.48	2885.44	29883.95	2.39862	0.000874	0.237992	5.775306	up
4-Tetradecanamidobenzylph osphonic acid	Benzene and substituted derivatives	Benzene and substituted derivatives	C21H36NO4P	3	0.883	–	2978.6	3485.68	2699.93	494.43	142.33	132.07	562.52	177.63	85.17	791.24	1082.99	2.384662	0.001315	0.237992	7.04591	up
Chlorthiophos	Organic acid and Its derivatives	Phosphoric acids	C11H15Cl2O3PS2	3	0.5587	60238-56-4 | 21923-23-9	17543.71	14207.7	15480.56	31964.04	34785.37	17935.04	20636.11	33124.24	31337.85	28361.42	27853.45	2.384267	0.000492	0.237992	0.557315	down
Threoninyl-Isoleucine	Amino acid and Its metabolites	Small Peptide	C10H20N2O4	1b	0.715	–	5829.15	11423.99	8400.87	23056.62	13421.37	18895.64	13371.15	24324.49	17489.57	12075.85	17125.83	2.371419	0.007421	0.237992	0.489485	down

Note: CRAB: Carbapenem-resistant *Acinetobacter baumannii*; CREC: Carbapenem-resistant *Escherichia coli*; VIP: Variable Importance in Projection. Values represent QC-SVR-corrected peak areas summarized as mean per participant; VIP scores derive from the combined positive/negative-ion OPLS-DA model. Compounds: Name of the metabolite; Class I: Primary class of the metabolite; Class II: Secondary class of the metabolite; Formula: Molecular formula of the metabolite; Level: Metabolite identification level (1a: indicates metabolite identification based on a high-confidence match to a standard using MS1, RT, and MS2 data; 1b: indicates metabolite identification based on a moderate-confidence match to a standard using MS1, RT, and MS2 data; 2: indicates metabolite identification based on a high-confidence match (without a standard) using MS1, RT, and MS2 data; 3: indicates metabolite identification based on a moderate-confidence match (without a standard) using MS1, RT, and MS2 data; 4: indicates metabolite identification based on a match using MS1 and RT data only; metabolites at this level are not included in the differential analysis); Score: Metabolite identification score; CAS: Metabolite CAS number; VIP: Variable Importance in Projection; FDR: False Discovery Rate; Type: Up/down regulation type of the metabolite.

### 3.3. Differential metabolite KEGG analysis between CRAB and CREC groups

**Fig 4:**
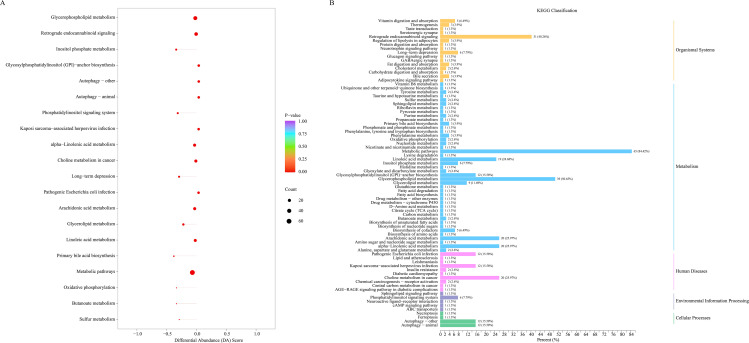
Differential metabolite Kyoto Encyclopedia of Genes and Genomes (KEGG) analysis between *Carbapenem-resistant Acinetobacter baumannii* (CRAB) and *Carbapenem-resistant Escherichia coli* (CREC) groups. (A) KEGG enrichment analysis. (B) KEGG classification. n = 8 in the CRAB group, and n = 3 in the CREC group..

KEGG analysis showed that differential metabolites were primarily associated with metabolism, clustering predominantly in Metabolic pathways (84.42%) and Glycerophospholipid metabolism (50.65%) pathways (Fig 4).

### 3.4. Correlation between Metabolomic Signatures and Microbial Community

**Fig 5:**
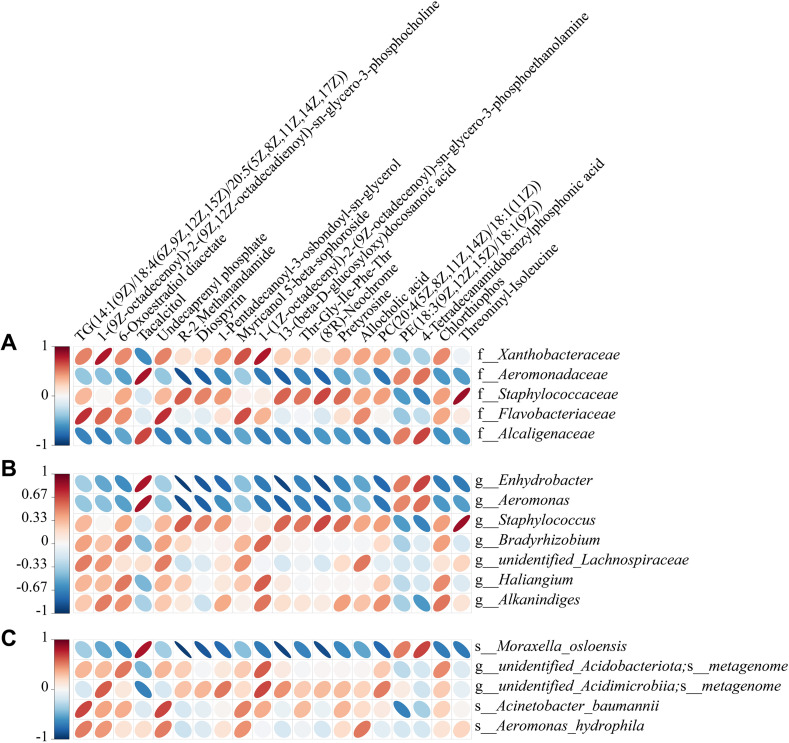
Correlation heatmap of top 20 differential metabolites and differential microbiota. (A) Correlation heatmap of top 20 differential metabolites and differential microbiota at the family level. (B) Correlation heatmap of top 20 differential metabolites and differential microbiota at the genus level. (C) Correlation heatmap of top 20 differential metabolites and differential microbiota at the species level. Rows represent microbiota, columns represent metabolites. Red ellipses indicate positive correlations; blue ellipses indicate negative correlations. The thinner the ellipse, the greater the absolute value of the correlation. Blank cells indicate *P* *>* 0.05.

Correlation analysis revealed that TG (14:1(9Z)/18:4(6Z,9Z,12Z,15Z)/20:5(5Z,8Z,11Z,14Z,17Z)) was positively correlated with microbes within the *Flavobacteriaceae* family and inversely associated with those within the *Alcaligenaceae* family (Fig 5A). At the *Enhydrobacter* genus and *Moraxella osloensis* species levels, 14 differential metabolites showed correlations (Fig 5B and 5C). Notably, tacalcitol and 4-tetradecanamidobenzylphosphonic acid exhibited positive correlations, while the remaining 12 metabolites showed negative correlations. Among the top 20 differential metabolites, R-2 Methanandamide and 13-(β-D-glucosyloxy) docosanoic acid demonstrated the strongest correlations with microbes at the *Enhydrobacter* genus and *Moraxella osloensis* species levels (Tables 2–4). However, no differential metabolites with correlation coefficients |r| ≥ 0.9 were observed at the family level (S5 Table).

**Table 2:** CREC_vs_CRAB. order. spearman correlation. Spearman correlation coefficient |r| ≥ 0.9 and *P* < 0.05.

Index	Taxonomy	Correlation	*P*-value	Compounds
MW0109935	k__*Bacteria;* p__*Firmicutes;* c__*Bacilli;* o__*unidentified_Bacilli*	0.907097322686796	0.000116077270185678	Threoninyl-Isoleucine

Note: CRAB: Carbapenem-resistant *Acinetobacter baumannii*; CREC: Carbapenem-resistant *Escherichia coli*;

**Table 3:** CREC_vs_CRAB. genus. spearman correlation. Spearman correlation coefficient |r| ≥ 0.9 and *P* < 0.05.

Index	Taxonomy	Correlation	*P*-value	Compounds
MW0063299	k__*Bacteria;* p__*Proteobacteria;* c__*Gammaproteobacteria;* o__*Pseudomonadales;* f__*Moraxellaceae;* g__*Enhydrobacter*	−0.943927963353136	0.0000126338424246318	R-2 Methanandamide
MW0141373	k__*Bacteria;* p__*Proteobacteria;* c__*Gammaproteobacteria;* o__*Pseudomonadales;* f__*Moraxellaceae;* g__*Enhydrobacter*	−0.915324085675769	0.0000774159252418635	13-(beta-D-glucosyloxy) docosanoic acid

Note: CRAB: Carbapenem-resistant *Acinetobacter baumannii*; CREC: Carbapenem-resistant *Escherichia coli*;

**Table 4:** CREC_vs_CRAB. species. spearman correlation. Spearman correlation coefficient |r| ≥ 0.9 and *P* < 0.05.

Index	Taxonomy	Correlation	*P*-value	Compounds
MW0063299	k__*Bacteria;* p__*Proteobacteria;* c__*Gammaproteobacteria;* o__*Pseudomonadales;* f__*Moraxellaceae;* g__*Enhydrobacter;* s__*Moraxella_osloensis*	−0.943927963353136	0.0000126338424246318	R-2 Methanandamide
MW0145216	k__*Bacteria;* p__*Proteobacteria;* c__*Gammaproteobacteria;* o__*Pseudomonadales;* f__*Moraxellaceae;* g__*Enhydrobacter;* s__*Moraxella_osloensis*	−0.915324085675769	0.0000774159252418635	Aquayamycin
MW0141373	k__*Bacteria;* p__*Proteobacteria;* c__*Gammaproteobacteria;* o__*Pseudomonadales;* f__*Moraxellaceae;* g__*Enhydrobacter;* s__*Moraxella_osloensis*	−0.915324085675769	0.0000774159252418635	13-(beta-D-glucosyloxy)docosanoic acid

Note: CRAB: Carbapenem-resistant *Acinetobacter baumannii*; CREC: Carbapenem-resistant *Escherichia coli*.

## 4. Discussion

This study elucidates the distinctive characteristics of CRAB and CREC infections in patients with PAS through an integrated microbiomics and metabolomics approach. Our findings offer new understanding of the infection processes employed by such resistant strains and how they affect host metabolism, while also suggesting potential avenues for diagnostic biomarkers and therapeutic targets. We observed notable variations in the structure of microbial communities in CRAB and CREC-infected patients. The CRAB group exhibited a reduced proportion of *Cyanobacteria* and an increased proportion of *Proteobacteria* in relation to the CREC group. This finding aligns with Ceccarani et al., who reported *Proteobacteria* overgrowth in the gut microbiome of patients with glycogen storage disease [33]. These compositional differences may reflect the specific impacts of different resistant bacteria on the host microecological environment, potentially explaining why no metabolites with high correlation coefficients (|r| ≥ 0.9) were observed at the genus level in our study.

Notably, we identified significant differences in the genera *Enhydrobacter* and *Moraxella osloensis* between the two patient groups, with *Moraxella osloensis* emerging as a potential biomarker. *Moraxella osloensis*, a Gram-negative bacterium, has been implicated in various infections. Lee et al. reported a fatal case of bacteremia caused by this organism in an immunocompromised patient [34]. Koleri et al. documented multiple bacteremia cases, further emphasizing its pathogenic potential [35]. In the realm of central nervous system infections, Li et al. described a rare case of acute meningitis attributed to *Moraxella osloensis* [36]. Richards et al. highlighted a case of simultaneous bacteremia and pneumonia caused by *Moraxella osloensis* in a 17-year-old patient [37]. These findings underscore the clinical significance of *Moraxella osloensis* in various infectious contexts.

Our metabolomics analysis revealed substantial metabolic differences between CRAB and CREC-infected patients, with most differential metabolites showing downregulation in the CRAB group. This suggests that CRAB infection may exert a broader inhibitory effect on host metabolism. Specifically, we found that amino acids and their metabolites were the primary differential metabolites, consistent with previous research on metabolic changes in sepsis patients. Yang et al. demonstrated that specific metabolite-receptor binding patterns might influence inflammatory responses and immune regulation [38]. Intriguingly, we discovered that R-2 Methanandamide and 13-(β-D-glucosyloxy) docosanoic acid exhibited high correlations with the genus *Enhydrobacter* and species *Moraxella osloensis*. This finding opens new avenues for in-depth research on the interactions between specific metabolites and microbes in PAS. Luz-Martínez et al. indicated that Methanandamide affects physiological functions through the TRPV1 channel [39]. These metabolites may play crucial roles in CRAB and CREC infection processes, offering potential targets for future therapeutic strategies.

R-2 Methanandamide, an endogenous cannabinoid receptor agonist, has not been explicitly studied in the context of PAS, CRAB, or CREC infections. However, research has shown that activation of the cannabinoid receptor CB2 can modulate inflammation, infection, and immune responses, potentially playing a significant role in pancreatitis and sepsis treatment [40]. Additionally, the cannabinoid receptor VR1 is upregulated in pancreatic cancer, with its expression level associated with prognosis, suggesting potential involvement in the pathogenesis of pancreatitis and sepsis [41]. 13-(β-D-glucosyloxy) docosanoic acid, a fatty acyl compound [42], has received limited attention in research. However, it has garnered some interest in plant physiology and pharmacology. The strong correlation of both R-2 Methanandamide and 13-(β-D-glucosyloxy) docosanoic acid with *Enhydrobacter* and *Moraxella osloensis* provides further microbiological and metabolomic insights into the microbial differences between CRAB and CREC infections.

The prevalence of CRAB and CREC among patients with PAS presents substantial hurdles for effective treatment and control. The urgent unmet clinical need lies in the rapid and accurate differentiation between these infections, as delays or inappropriate empirical therapy stemming from the days-long turnaround of traditional culture methods can significantly worsen patient prognosis and fuel antimicrobial resistance. Our study holds considerable promise in addressing this challenge. We identified *Moraxella osloensis* as significantly enriched in CREC infections, which, particularly when considered alongside correlated metabolites such as R-2 Methanandamide and 13-(β-D-glucosyloxy) docosanoic acid, suggests the potential for constructing a multi-marker signature. Such a signature could pave the way for the development of point-of-care diagnostic tools. Critically, these tools aim to provide pathogen-leaning information within hours, not days, enabling clinicians to initiate timely, targeted antimicrobial therapy or make informed adjustments to empirical regimens much sooner. Beyond their diagnostic potential, these findings hold significant implications for future therapeutic strategies and provide a foundation for precision medicine in PAS. The strong correlation observed between specific microbial taxa, such as *Moraxella osloensis*, and differential metabolites, like R-2 Methanandamide, highlights the endocannabinoid system and associated pathways as potential novel avenues for non-antibiotic, host-directed interventions [43,44]. The development of such strategies is critically important to circumvent established antibiotic resistance mechanisms. Furthermore, the capacity to rapidly differentiate between CRAB and CREC using the proposed biomarker signatures can directly inform clinical decision-making. For instance, early biomarker evidence suggestive of CRAB infection might prompt clinicians to consider earlier escalation to last-resort antibiotics and to institute rigorous monitoring for complications potentially linked to its unique metabolic disruption profile. Conversely, a signature indicative of CREC could guide alternative empirical therapeutic choices or different monitoring approaches. By dissecting these pathogen-specific host responses, this research moves beyond simple pathogen identification towards enabling truly personalized management strategies. Such strategies aim to target both the specific etiological agent and the host’s unique physiological response to it. Crucially, however, these preliminary findings require extensive validation before they can be considered for clinical implementation.

First, although this study provides valuable preliminary insights, the sample size was limited, particularly for the CREC subgroup, which restricts the statistical power and generalizability of our findings. As indicated by the permutation test results, while the very high R^2^Y value indicates strong explanatory power for the observed class separation, the moderate Q^2^ suggests limited predictive power. The strong correlation between *Moraxella osloensis* and key metabolites is noteworthy but requires validation in a larger cohort to rule out chance associations. Second, our analysis was limited to peripheral blood samples. While circulating biomarkers reflect systemic responses, they may underestimate local pathogenic processes in organs such as the pancreas or gut. Future work will involve collecting target organ tissues or specific bodily fluids to uncover molecular events more proximal to the site of pathology. Third, the cross-sectional nature of our sampling provides only a single-time-point snapshot, precluding the tracking of microbiome and metabolome dynamics throughout the infection process. We have planned a prospective longitudinal follow-up study involving multi-timepoint sampling to characterize temporal heterogeneity and validate the reproducibility of the identified signatures. Fourth, metabolite identification was performed by searching an in-house database, integrated public and predictive libraries, and using the metDNA approach, but these identifications lack quantitative validation with chemical standards. Therefore, based on the findings from the correlation and integrative analyses, targeted quantification of specific key metabolites will be performed in future mechanistic studies. Finally, as the serum samples were de-identified upon collection, we were unable to obtain complete clinical covariates for integrated analysis. Therefore, we are establishing a prospective multicenter cohort to concurrently collect standardized clinical phenotypic and metabolomic data. We will collaborate with a biostatistics team to perform multivariate modeling to assess whether these metabolites can predict the need for organ support, length of hospital stay, and treatment response. Concurrently, mechanistic studies focusing on R-2-methylarachidonylamine and 13-(β-D-glucopyranosyloxy) docosanoic acid will be pursued to advance their functional validation and translational potential.

## 5. Conclusion

In conclusion, this study reveals unique characteristics of CRAB and CREC infections in PAS patients through integrated microbiomics and metabolomic analyses. We identified *Moraxella osloensis* as a potential biomarker for differentiating CRAB and CREC infections, correlating with R-2 Methanandamide and 13-(β-D-glucosyloxy) docosanoic acid. These findings not only deepen our understanding of the infection mechanisms of these resistant strains but also provide new directions for developing future diagnostic and therapeutic strategies in the management of PAS. Future longitudinal studies will incorporate larger cohorts with pancreatic and/or gut microbiome samples collected over extended time periods to further investigate the underlying biomarker mechanisms.

## Supporting information

S1 Fig The agarose gel experiment monitored the negative control of 11 samples, and after two amplifications, the negative control did not show amplification bands, and the amplification was free of contamination. (TIF)

S2 Fig OPLS-DA model validation, permutation test (200 permutations). (TIF)

S3 Fig Differences in α-diversity and β-diversity between Carbapenem-resistant *Acinetobacter baumannii* (CRAB) and Carbapenem-resistant *Escherichia coli* (CREC) groups. (A-D) Analysis of α-diversity between two groups by Abundance-based Coverage Estimator (ACE) index, Chao1 index, Shannon index, and Simpson index. (E-H) Analysis of β-diversity between two groups by Non-Metric Multidimensional Scaling (NMDS), Principal Component Analysis (PCA), Principal Coordinate Analysis (PCoA), and Bray-Curtis analysis. n = 8 in the CRAB group, and n = 3 in the CREC group.
(TIF)

S4 Fig Differential microbiota analysis by Linear Discriminant Analysis Effect Size (LEfSe) analysis. (A) The family level. (B) The genus level. (C) The species level.
(TIF)

S5 Fig Sample quality control analysis. In positive ion mode, (A) Total ion current (TIC) chromatogram. (B) Extracted ion chromatogram (EIC) of the blank sample. (C) Pearson correlation analysis. (D) The distribution of Coefficients of Variation (CV). In negative ion mode, (E) TIC chromatogram. (F) EIC of the blank sample. (G) Pearson correlation analysis. (H) The distribution of CV.
(TIF)

S1 Table Baseline demographic and clinical characteristics. (DOCX)

S2 Table Minimum Inhibitory Concentration/Antibiotic Susceptibility Result. (DOCX)

S3 Table LEfSe analysis. (DOCX)

S4 Table Internal standard information. (DOCX)

S5 Table Correlation analysis between microorganisms and metabolites. (DOCX)

## Abbreviations

PAS: Pancreatitis-associated sepsis
CRAB: carbapenem-resistant *Acinetobacter baumannii*
CREC: carbapenem-resistant *Escherichia coli*
LC-MS: liquid chromatography-mass spectrometry
OTU: operational taxonomic unit
NMDS: non-metric multidimensional scaling
PCoA: principal coordinate analysis
LDA: linear discriminant analysis
LEfSe: linear discriminant analysis effect size
UPLC-MS/MS: ultra-performance liquid chromatography-tandem mass spectrometry
GC-MS: gas chromatography-mass spectrometry
KEGG: Kyoto encyclopedia of genes and genomes
HMDB: human metabolome database
TG: triglyceride
VIP: variable importance in projection
OPLS-DA: orthogonal partial least squares discriminant analysis
PCA: principal component analysis
MDRO: multidrug-resistant organism
AP: acute pancreatitis
IDA: information-dependent acquisition
