# Supplementary figures and images for: Integrated microbiomics and metabolomics analysis reveals distinct profiles in carbapenem-resistant Acinetobacter baumannii and Escherichia coli infections in Pancreatitis-associated sepsis
Source: PLoS One. 2026 Feb 10;21(2):e0340895. doi: 10.1371/journal.pone.0340895 (PMC12890157; doi:10.1371/journal.pone.0340895)

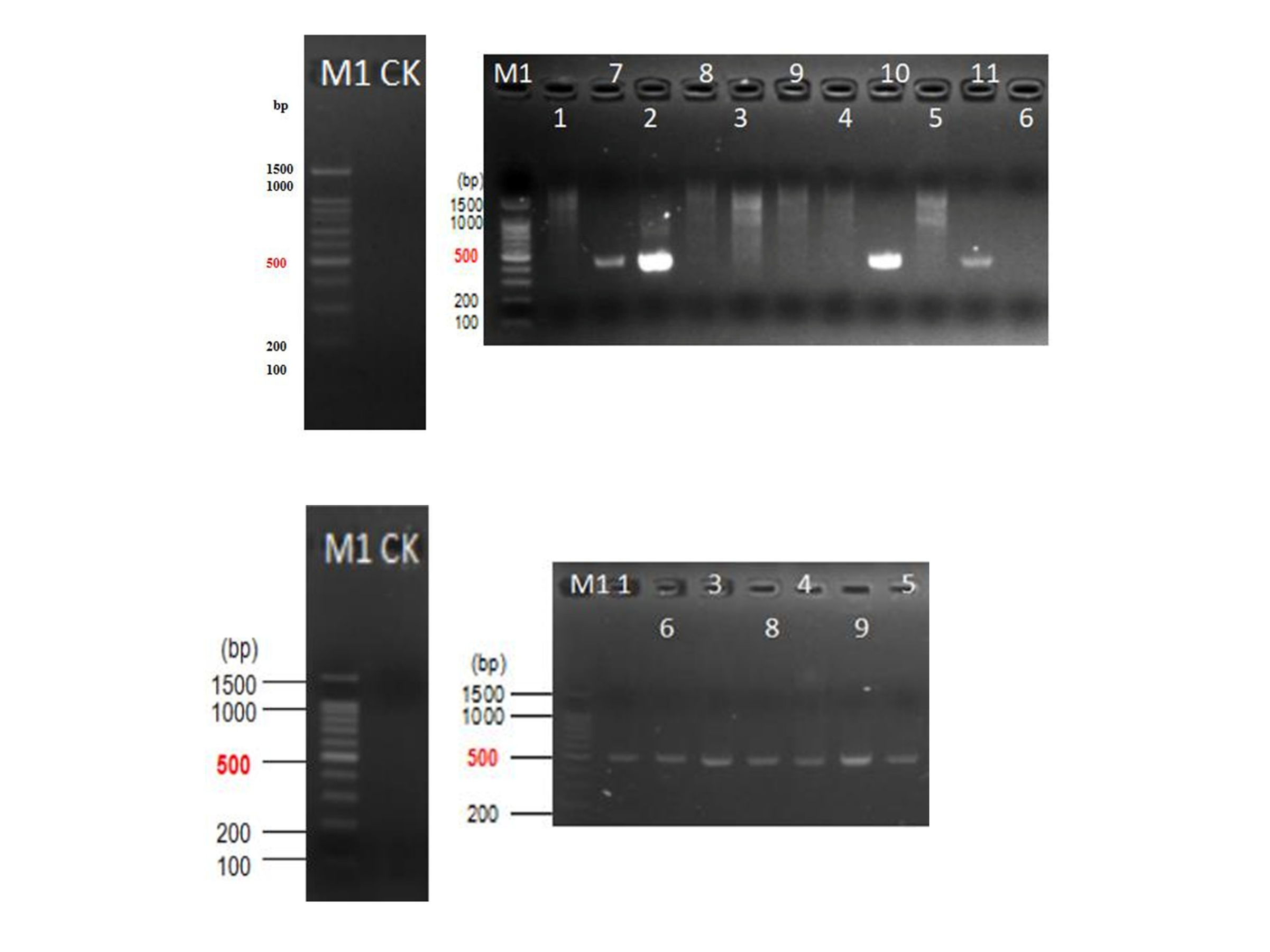

Supplement: S1 Fig — (TIF) [file pone.0340895.s001.tif]

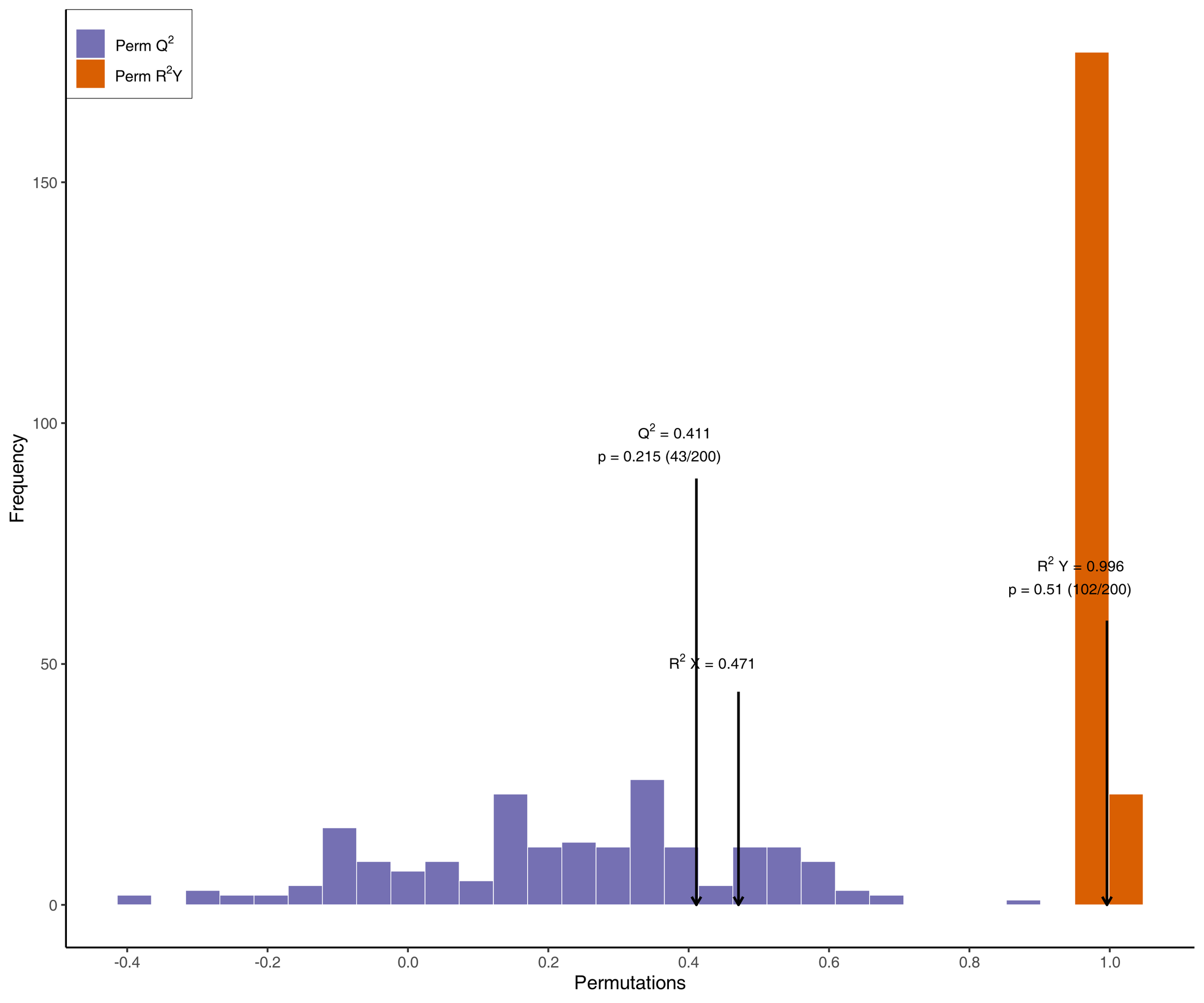

Supplement: S2 Fig — (TIF) [file pone.0340895.s002.tif]

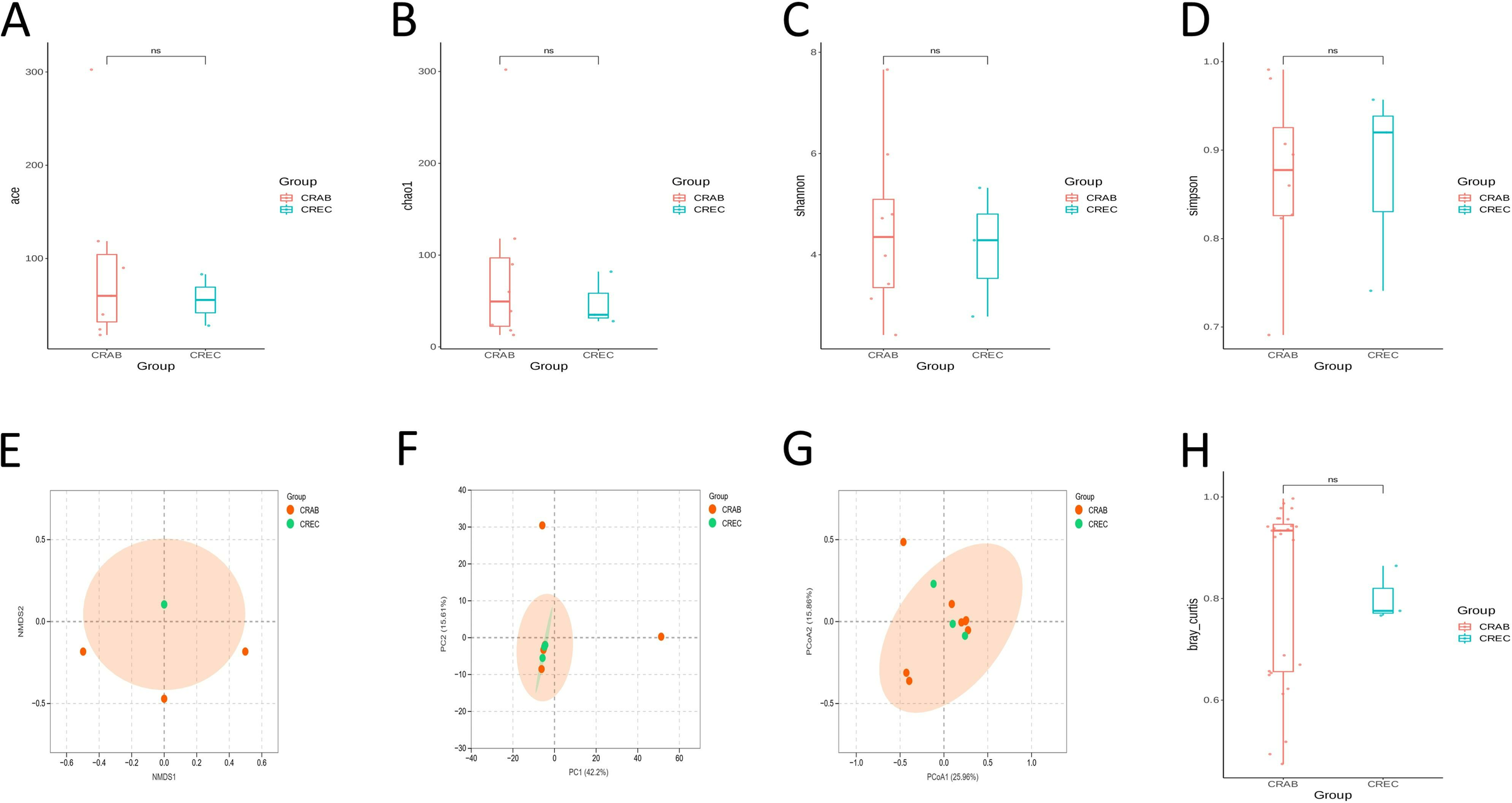

Supplement: S3 Fig — (A-D) Analysis of α-diversity between two groups by Abundance-based Coverage Estimator (ACE) index, Chao1 index, Shannon index, and Simpson index. (E-H) Analysis of β-diversity between two groups by Non-Metric Multidimensional Scaling (NMDS), Principal Component Analysis (PCA), Principal Coordinate Analysis (PCoA), and Bray-Curtis analysis. n = 8 in the CRAB group, and n = 3 in the CREC group. (TIF) [file pone.0340895.s003.tif]

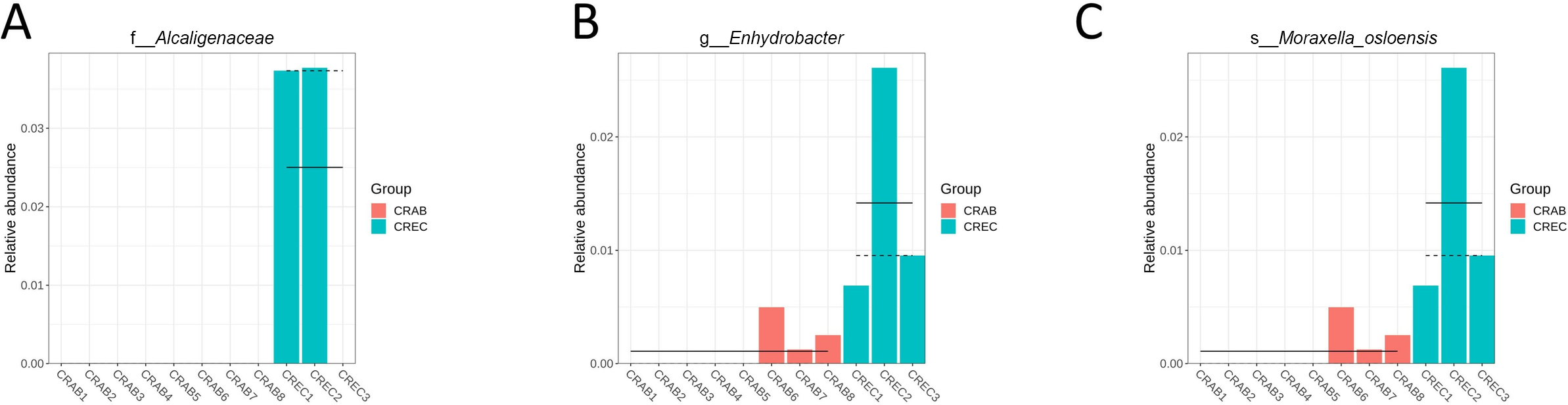

Supplement: S4 Fig — (A) The family level. (B) The genus level. (C) The species level. (TIF) [file pone.0340895.s004.tif]

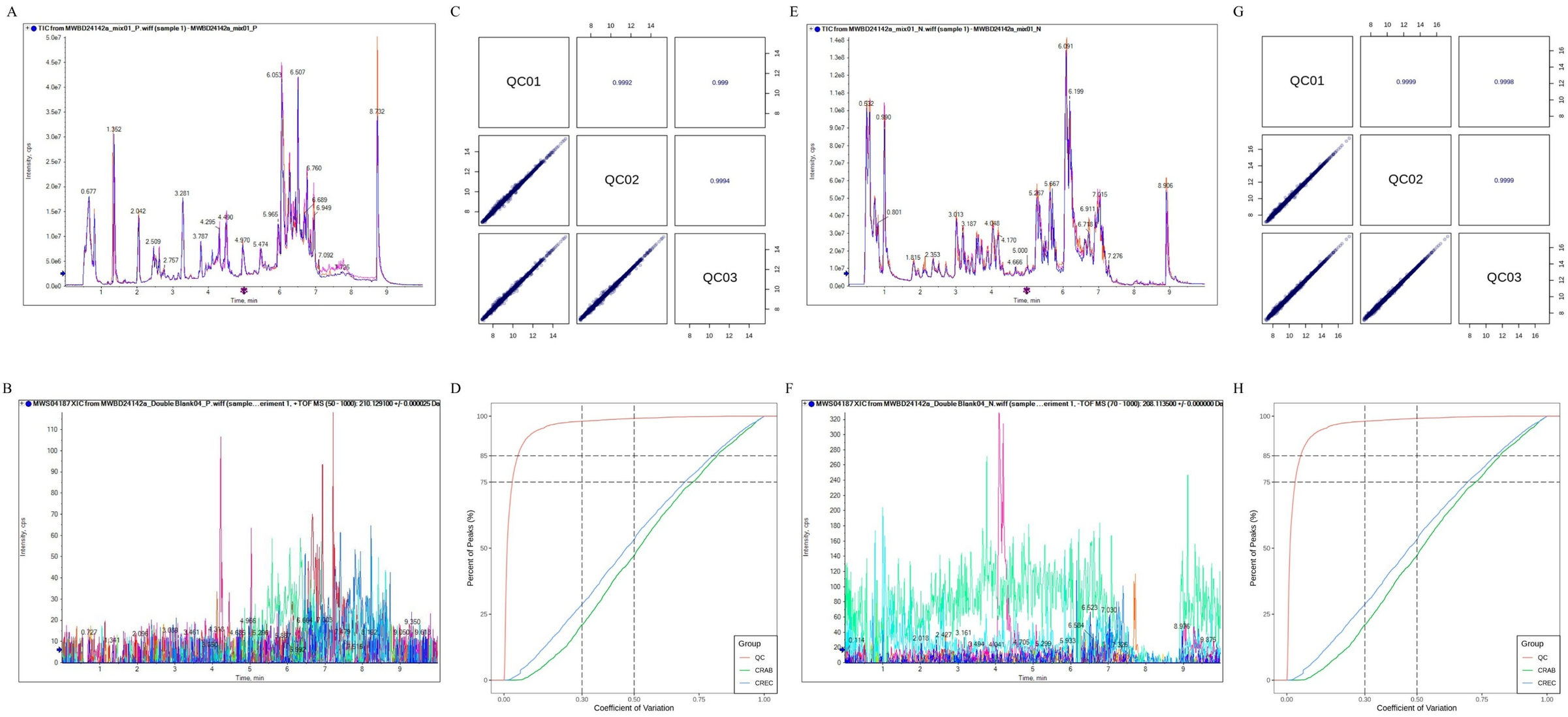

Supplement: S5 Fig — In positive ion mode, (A) Total ion current (TIC) chromatogram. (B) Extracted ion chromatogram (EIC) of the blank sample. (C) Pearson correlation analysis. (D) The distribution of Coefficients of Variation (CV). In negative ion mode, (E) TIC chromatogram. (F) EIC of the blank sample. (G) Pearson correlation analysis. (H) The distribution of CV. (TIF) [file pone.0340895.s005.tif]
