# Supplementary material for: Integrated microbiomics and metabolomics analysis reveals distinct profiles in carbapenem-resistant Acinetobacter baumannii and Escherichia coli infections in Pancreatitis-associated sepsis
Source: PLoS One. 2026 Feb 10;21(2):e0340895. doi: 10.1371/journal.pone.0340895 (PMC12890157; doi:10.1371/journal.pone.0340895)
Supplement: S1 Table — (DOCX) [file pone.0340895.s006.docx]

S1 Table. Baseline demographic and clinical characteristics.

| Characteristic | CREC (n=3) | CRAB (n=8) | Overall (N=11) |
| --- | --- | --- | --- |
| Age, years | 60.0 (58.5 - 60.0) | 75.5 (59.2 - 81.0) | 62.0 (58.5 - 78.0) |
| BMI, kg/m^2 | 21.50 (20.84 - 21.68) | 21.80 (20.73 - 22.81) | 21.76 (20.58 - 22.32) |
| White blood cell count, 10^9/L | 4.78 (4.74 - 7.66) | 12.00 (9.07 - 14.81) | 10.54 (6.68 - 12.25) |
| C-reactive protein, mg/L | 14.3 (12.4 - 83.2) | 55.0 (16.7 - 113.7) | 23.0 (14.0 - 125.8) |
| Procalcitonin, ng/mL | 22.50 (14.87 - 61.25) | 8.16 (3.55 - 9.58) | 8.59 (5.76 - 11.73) |
| Platelet count, 10^9/L | 135 (100 - 136) | 128 (89 - 167) | 129 (88 - 148) |
| Serum creatinine, μmol/L | 76 (60 - 180) | 56 (50 - 84) | 57 (49 - 90) |
| Treatment duration, days | 41 (39 - 68) | 41 (30 - 59) | 41 (34 - 64) |
| Sex - Male, n (%) | 3 (100.0) | 4 (50.0) | 7 (63.6) |
| Sex - Female, n (%) | 0 (0.0) | 4 (50.0) | 4 (36.4) |
| Comorbidity - Enteric fistula, n (%) | 1 (33.3) | 0 (0.0) | 1 (9.1) |
| Comorbidity - Pancreatic fistula, n (%) | 1 (33.3) | 2 (25.0) | 3 (27.3) |
| Comorbidity - Biliary leak, n (%) | 1 (33.3) | 2 (25.0) | 3 (27.3) |
| Comorbidity - None, n (%) | 0 (0.0) | 4 (50.0) | 4 (36.4) |
| Fever present, n (%) | 3 (100.0) | 8 (100.0) | 11 (100.0) |
| Infection site - Abdominal cavity, n (%) | 3 (100.0) | 4 (50.0) | 7 (63.6) |
| Infection site - Pulmonary, n (%) | 0 (0.0) | 4 (50.0) | 4 (36.4) |
| Treatment - Surgical management, n (%) | 3 (100.0) | 4 (50.0) | 7 (63.6) |
| Treatment - Mechanical ventilation, n (%) | 0 (0.0) | 4 (50.0) | 4 (36.4) |

Notes: Continuous variables are reported as median (IQR); categorical variables are n (%).
