# Supplementary material for: Integrated microbiomics and metabolomics analysis reveals distinct profiles in carbapenem-resistant Acinetobacter baumannii and Escherichia coli infections in Pancreatitis-associated sepsis
Source: PLoS One. 2026 Feb 10;21(2):e0340895. doi: 10.1371/journal.pone.0340895 (PMC12890157; doi:10.1371/journal.pone.0340895)
Supplement: S2 Table — (DOCX) [file pone.0340895.s007.docx]

| S2 Table. Minimum Inhibitory Concentration/Antibiotic Susceptibility Result. | | | | | | | | | | | | | |
| --- | --- | --- | --- | --- | --- | --- | --- | --- | --- | --- | --- | --- | --- |
| No | Antibiotics | Patients | | | | | | | | | | | Breakpoint |
|  |  | CREC1 | CREC2 | CREC3 | CRAB1 | CRAB2 | CRAB3 | CRAB4 | CRAB5 | CRAB6 | CRAB7 | CRAB8 |  |
| 1 | Amikacin | R/＞64 | R/＞64 | R/＞64 | R/＞64 | R/＞64 | R/＞64 | R/＞64 | R/＞64 | R/＞64 | R/＞64 | R/＞64 | S≤16,R≥64 |
| 2 | Ceftazidime | R/＞32 | R/＞32 | R/16 | R/＞32 | R/＞32 | R/＞32 | R/＞32 | R/＞32 | R/16 | R/＞32 | R/＞32 | S≤4,R≥16 |
| 3 | Ciprofloxacin | R/＞4 | R/＞4 | R/＞4 | R/＞4 | R/＞4 | R/＞4 | R/＞4 | R/＞4 | R/＞4 | R/＞4 | R/＞4 | S≤0.25,R≥1 |
| 4 | Colistin | S/≤1 | S/≤1 | S/≤1 | S/≤1 | S/≤1 | S/≤1 | S/≤1 | S/≤1 | S/≤1 | S/≤1 | S/≤1 | S≤2,R＞2 |
| 5 | Ceftriaxone | R/＞32 | R/＞32 | R/＞32 | R/＞32 | R/＞32 | R/＞32 | R/＞32 | R/＞32 | R/＞32 | R/＞32 | R/＞32 | S≤1,R≥4 |
| 6 | Cefoperazone | R/＞16 | R/＞16 | R/＞16 | R/＞16 | R/＞16 | R/＞16 | R/＞16 | R/＞16 | R/＞16 | R/＞16 | R/＞16 | S≤2,R≥16 |
| 7 | Gentamicin | R/＞8 | R/＞8 | R/＞8 | R/＞8 | R/＞8 | R/＞8 | R/＞8 | R/＞8 | R/＞8 | R/＞8 | R/＞8 | S≤4,R≥16 |
| 8 | Imipenem | R/＞8 | R/＞8 | S/0.5 | R/＞8 | R/＞8 | R/＞8 | R/＞8 | R/＞8 | R/＞8 | R/＞8 | R/＞8 | S≤1,R≥4 |
| 9 | Levofloxacin | R/＞8 | R/＞8 | R/＞8 | R/＞8 | R/＞8 | R/＞8 | R/＞8 | R/＞8 | R/＞8 | R/＞8 | R/＞8 | S≤0.5,R≥2 |
| 10 | Meropenem | R/＞8 | R/＞8 | S/0.5 | R/＞8 | R/＞8 | R/＞8 | R/＞8 | R/＞8 | R/＞8 | R/＞8 | R/＞8 | S≤1,R≥4 |
| 11 | Minocycline | R/＞16 | I/8 | R/16 | I/8 | R16 | I/8 | S/4 | I/8 | R/16 | I/8 | I/8 | S≤4,R≥16 |
| 12 | Tobramycin | R/＞8 | R/＞8 | R/＞8 | R/＞8 | R/＞8 | R/＞8 | R/＞8 | R/＞8 | R/＞8 | R/＞8 | R/＞8 | S≤4,R≥16 |
| 13 | Ampicillin/Sulbactam | R/＞16/8 | R/＞16/8 | R/＞16/8 | R/＞16/8 | R/＞16/8 | R/＞16/8 | R/＞16/8 | R/＞16/8 | R/＞16/8 | R/＞16/8 | R/＞16/8 | S≤8,R≥32 |
| 14 | Cefoperazone/Sulbactam | R/＞32/8 | R/＞16/8 | R/＞32/8 | R/＞32/8 | R/＞32/8 | R/＞32/8 | R/＞32/8 | R/＞32/8 | R/＞32/8 | R/＞32/8 | R/＞32/8 | S≤16,R≥64 |
| 15 | Trimethoprim/Sulfamethoxazole | R/＞4/76 | S/≤1/19 | R/＞4/76 | R/＞4/76 | R/＞4/76 | R/＞4/76 | R/＞4/76 | R/＞4/76 | R/＞4/76 | R/＞4/76 | R/＞4/76 | S≤2,R≥4 |
| 16 | Piperacillin/Tazobactam | R/＞64/4 | R/＞64/4 | R/＞64/4 | R/＞64/4 | R/＞64/4 | R/＞64/4 | R/＞64/4 | R/＞64/4 | R/＞64/4 | R/＞64/4 | R/＞64/4 | S≤8,R≥32 |
| 17 | Tigecycline | S/2 | S/2 | S/2 | S/1 | S/1 | S/2 | I/4 | S/2 | S/2 | S/2 | S/2 | S≤2,R≥8 |

Note: S, Sensitive; I, intermediary; R, drug resistance; CRAB, Carbapenem-resistant *Acinetobacter baumannii*; CREC, Carbapenem-resistant *Escherichia coli*.
