# Supplementary material for: Integrated microbiomics and metabolomics analysis reveals distinct profiles in carbapenem-resistant Acinetobacter baumannii and Escherichia coli infections in Pancreatitis-associated sepsis
Source: PLoS One. 2026 Feb 10;21(2):e0340895. doi: 10.1371/journal.pone.0340895 (PMC12890157; doi:10.1371/journal.pone.0340895)
Supplement: S3 Table — (DOCX) [file pone.0340895.s008.docx]

S3 Table. LEfSe analysis.

| Taxonomy | Abundace | Group | LDA_score | P-value | CRAB1 | CRAB2 | CRAB3 | CRAB4 | CRAB5 | CRAB6 | CRAB7 | CRAB8 | CREC1 | CREC2 | CREC3 |
| --- | --- | --- | --- | --- | --- | --- | --- | --- | --- | --- | --- | --- | --- | --- | --- |
| k__*Bacteria;* p__*Acidobacteriota* | 4.778597 | CRAB | 4.236458 | 0.520703 | 0 | 0.216998 | 0 | 0.063362 | 0 | 0 | 0.190541 | 0 | 0.001423 | 0.036563 | 0.057068 |
| k__*Bacteria;* p__*Actinobacteria* | 4.772514 | CREC | 4.346529 | 0.682408 | 0.09336 | 0.014051 | 0 | 0.019148 | 0.017851 | 0.047632 | 0.011249 | 0 | 0.015536 | 0.161748 | 0.000385 |
| k__*Bacteria;* p__*Actinobacteriota* | 4.64932 | CREC | 4.198684 | 0.473952 | 0.019614 | 0.057638 | 0 | 0.025451 | 0.017437 | 0.020998 | 0.010402 | 0.011343 | 0.038395 | 0 | 0.093158 |
| k__*Bacteria;* p__*Armatimonadota* | 2.862888 | CRAB | 2.525502 | 0.363722 | 0 | 0.002617 | 0 | 0 | 0 | 0 | 0.003084 | 0 | 0 | 0 | 0 |
| k__*Bacteria;* p__*Bacteroidota* | 4.733262 | CRAB | 4.139683 | 0.307434 | 0.007822 | 0.020313 | 0.00287 | 0.020272 | 0.036976 | 0.152683 | 0.02776 | 0.163043 | 0.046934 | 0.030074 | 0.057276 |
| k__*Bacteria;* p__*Bdellovibrionota* | 3.170856 | CRAB | 2.76542 | 0.363722 | 0 | 0.008973 | 0 | 0 | 0 | 0 | 0.002601 | 0 | 0 | 0 | 0 |
| k__*Bacteria;* p__*Cyanobacteria* | 5.506763 |  |  |  | 0.449261 | 0.066237 | 0.481966 | 0.346375 | 0.557891 | 0.013969 | 0.057637 | 0.299283 | 0.200694 | 0.279052 | 0.472458 |
| k__*Bacteria;* p__*Deinococcota* | 4.149312 | CREC | 3.741702 | 0.363722 | 0 | 0 | 0 | 0 | 0 | 0.019248 | 0 | 0 | 0.042309 | 0 | 0 |
| k__*Bacteria;* p__*Desulfobacterota* | 3.869833 | CRAB | 3.568469 | 0.636328 | 0 | 0.007166 | 0 | 0 | 0.030611 | 0 | 0 | 0.021324 | 0.005396 | 0 | 0 |
| k__*Bacteria;* p__*Elusimicrobiota* | 2.750502 | CRAB | 2.475714 | 0.540291 | 0 | 0.004393 | 0 | 0 | 0 | 0 | 0 | 0 | 0 | 0 | 0 |
| k__*Bacteria;* p__*Firmicutes* | 5.456317 | CREC | 4.578788 | 0.838256 | 0.24577 | 0.002617 | 0.315768 | 0.138414 | 0.170017 | 0.564404 | 0.28371 | 0.39785 | 0.456386 | 0.22243 | 0.174879 |
| k__*Bacteria;* p__*Gemmatimonadota* | 4.281735 | CREC | 3.997431 | 0.603595 | 0 | 0.021653 | 0 | 0 | 0 | 0 | 0.003659 | 0 | 0 | 0.057393 | 0 |
| k__*Bacteria;* p__*Methylomirabilota* | 2.774469 | CRAB | 2.472385 | 0.540291 | 0 | 0.004642 | 0 | 0 | 0 | 0 | 0 | 0 | 0 | 0 | 0 |
| k__*Bacteria;* p__*Myxococcota* | 4.275856 | CREC | 3.969651 | 0.813107 | 0 | 0.036109 | 0 | 0.038029 | 0 | 0 | 0.006774 | 0 | 0 | 0 | 0.055291 |
| k__*Bacteria;* p__*Nitrospirota* | 2.654361 | CRAB | 2.419313 | 0.540291 | 0 | 0.003521 | 0 | 0 | 0 | 0 | 0 | 0 | 0 | 0 | 0 |
| k__*Bacteria;* p__*Planctomycetes* | 3.287317 |  |  |  | 0 | 0.011496 | 0.003699 | 0 | 0 | 0 | 0 | 0 | 0 | 0.001748 | 0 |
| k__*Bacteria;* p__*Planctomycetota* | 2.705086 | CRAB | 2.509733 | 0.544827 | 0 | 0.003957 | 0 | 0 | 0 | 0 | 0 | 0 | 0.000801 | 0 | 0 |
| k__*Bacteria;* p__*Proteobacteria* | 5.387382 | CRAB | 4.63158 | 0.683091 | 0.184172 | 0.441817 | 0.181052 | 0.344273 | 0.169218 | 0.156064 | 0.376637 | 0.078604 | 0.187559 | 0.205333 | 0.065928 |
| k__*Bacteria;* p__*Spirochaetota* | 3.362944 | CRAB | 2.980849 | 0.540291 | 0 | 0 | 0 | 0 | 0 | 0 | 0 | 0.018452 | 0 | 0 | 0 |
| k__*Bacteria;* p__*Thermotogota* | 3.101211 | CRAB | 2.784837 | 0.15332 | 0 | 0 | 0 | 0 | 0 | 0 | 0 | 0.0101 | 0.002283 | 0 | 5.93E-05 |
| k__*Bacteria;* p__*unidentified_Bacteria* | 3.788032 | CRAB | 3.461195 | 0.636328 | 0 | 0.013708 | 0.010001 | 0 | 0 | 0.025002 | 0 | 0 | 0.002283 | 0 | 0 |
| k__*Bacteria;* p__*Verrucomicrobiota* | 3.778276 |  |  |  | 0 | 0.037449 | 0 | 0.004676 | 0 | 0 | 0.004838 | 0 | 0 | 0.005659 | 0 |
| k__*Bacteria;* p__*Acidobacteriota;* c__*Acidobacteriae* | 4.636306 |  |  |  | 0 | 0.156619 | 0 | 0 | 0 | 0 | 0.153346 | 0 | 0 | 0 | 0.057068 |
| k__*Bacteria;* p__*Acidobacteriota;* c__*Blastocatellia* | 3.293363 |  |  |  | 0 | 0.008817 | 0 | 0 | 0 | 0 | 0.005171 | 0 | 0.001423 | 0 | 0 |
| k__*Bacteria;* p__*Acidobacteriota;* c__*Holophagae* | 3.214624 | CRAB | 3.029072 | 0.540291 | 0 | 0.011465 | 0 | 0 | 0 | 0 | 0 | 0 | 0 | 0 | 0 |
| k__*Bacteria;* p__*Acidobacteriota;* c__*Thermoanaerobaculia* | 2.697994 | CRAB | 2.492672 | 0.540291 | 0 | 0.003489 | 0 | 0 | 0 | 0 | 0 | 0 | 0 | 0 | 0 |
| k__*Bacteria;* p__*Acidobacteriota;* c__*unidentified_Acidobacteriota* | 3.683259 | CRAB | 3.371269 | 0.363722 | 0 | 0.020937 | 0 | 0 | 0 | 0 | 0.013426 | 0 | 0 | 0 | 0 |
| k__*Bacteria;* p__*Acidobacteriota;* c__*Vicinamibacteria* | 4.228713 |  |  |  | 0 | 0.010718 | 0 | 0.063362 | 0 | 0 | 0.018597 | 0 | 0 | 0.036563 | 0 |
| k__*Bacteria;* p__*Actinobacteria;* c__*unidentified_Actinobacteria* | 4.908084 | CREC | 4.447796 | 0.682408 | 0.09336 | 0.014051 | 0 | 0.019148 | 0.017851 | 0.027405 | 0.010282 | 0 | 0.015536 | 0.160593 | 0.000385 |
| k__*Bacteria;* p__*Actinobacteriota;* c__*Acidimicrobiia* | 4.255937 | CREC | 4.024207 | 0.813107 | 0 | 0.01536 | 0 | 0.007221 | 0 | 0 | 0.000665 | 0 | 0 | 0 | 0.02726 |
| k__*Bacteria;* p__*Actinobacteriota;* c__*Coriobacteriia* | 3.736152 | CREC | 3.294976 | 0.795158 | 0 | 0 | 0 | 0 | 0 | 0.015956 | 0 | 0.011343 | 0.013431 | 0 | 0 |
| k__*Bacteria;* p__*Actinobacteriota;* c__*Thermoleophilia* | 4.730006 | CREC | 4.368118 | 0.680331 | 0.019614 | 0.036919 | 0 | 0.01823 | 0.017437 | 0.005042 | 0.007016 | 0 | 0.024964 | 0 | 0.065898 |
| k__*Bacteria;* p__*Armatimonadota;* c__*Armatimonadia* | 2.573055 | CRAB | 2.319271 | 0.540291 | 0 | 0.002617 | 0 | 0 | 0 | 0 | 0 | 0 | 0 | 0 | 0 |
| k__*Bacteria;* p__*Armatimonadota;* c__*Fimbriimonadia* | 2.623452 | CRAB | 2.046167 | 0.540291 | 0 | 0 | 0 | 0 | 0 | 0 | 0.003084 | 0 | 0 | 0 | 0 |
| k__*Bacteria;* p__*Bacteroidota;* c__*Bacteroidia* | 4.857927 | CRAB | 4.264225 | 0.414216 | 0.007822 | 0.020313 | 0.00287 | 0.020272 | 0.036976 | 0.152683 | 0.02776 | 0.163043 | 0.046934 | 0.030074 | 0.057276 |
| k__*Bacteria;* p__*Bdellovibrionota;* c__*Oligoflexia* | 3.214078 | CRAB | 2.966635 | 0.363722 | 0 | 0.008973 | 0 | 0 | 0 | 0 | 0.002601 | 0 | 0 | 0 | 0 |
| k__*Bacteria;* p__*Cyanobacteria;* c__*Sericytochromatia* | 1.991199 | CRAB | 1.749295 | 0.540291 | 0 | 0.000685 | 0 | 0 | 0 | 0 | 0 | 0 | 0 | 0 | 0 |
| k__*Bacteria;* p__*Cyanobacteria;* c__*unidentified_Cyanobacteria* | 4.06772 | CREC | 3.70805 | 0.363722 | 0 | 0 | 0 | 0 | 0 | 0 | 0 | 0.005805 | 0.028819 | 0 | 0 |
| k__*Bacteria;* p__*Deinococcota;* c__*Deinococci* | 4.234478 | CREC | 3.920574 | 0.363722 | 0 | 0 | 0 | 0 | 0 | 0.019248 | 0 | 0 | 0.042309 | 0 | 0 |
| k__*Bacteria;* p__*Desulfobacterota;* c__*Desulfovibrionia* | 2.451724 | CRAB | 2.123542 | 0.540291 | 0 | 0 | 0 | 0 | 0 | 0 | 0 | 0.001599 | 0 | 0 | 0 |
| k__*Bacteria;* p__*Desulfobacterota;* c__*Syntrophia* | 3.937254 | CRAB | 3.796088 | 0.540291 | 0 | 0 | 0 | 0 | 0.030611 | 0 | 0 | 0 | 0 | 0 | 0 |
| k__*Bacteria;* p__*Desulfobacterota;* c__*Syntrophorhabdia* | 3.542805 | CRAB | 3.219987 | 0.540291 | 0 | 0 | 0 | 0 | 0 | 0 | 0 | 0.019725 | 0 | 0 | 0 |
| k__*Bacteria;* p__*Firmicutes;* c__*Bacilli* | 5.2994 | CRAB | 4.562938 | 0.683091 | 0.043376 | 0.001464 | 0.148711 | 0.038118 | 0.156281 | 0.222321 | 0.276573 | 0.19817 | 0.232655 | 0.126933 | 0.030697 |
| k__*Bacteria;* p__*Firmicutes;* c__*Clostridia* | 5.353514 | CREC | 4.609684 | 0.683091 | 0.202394 | 0.001153 | 0.167056 | 0.100296 | 0.013736 | 0.265356 | 0.007137 | 0.181051 | 0.212346 | 0.095496 | 0.144182 |
| k__*Bacteria;* p__*Firmicutes;* c__*Negativicutes* | 4.121442 |  |  |  | 0 | 0 | 0 | 0 | 0 | 0.076727 | 0 | 0.018629 | 0.011385 | 0 | 0 |
| k__*Bacteria;* p__*Gemmatimonadota;* c__*Gemmatimonadetes* | 4.424527 | CREC | 4.056301 | 0.603595 | 0 | 0.021653 | 0 | 0 | 0 | 0 | 0.003659 | 0 | 0 | 0.057393 | 0 |
| k__*Bacteria;* p__*Myxococcota;* c__*Myxococcia* | 4.563061 | CREC | 4.386704 | 0.603595 | 0 | 0.008443 | 0 | 0 | 0 | 0 | 0.003175 | 0 | 0 | 0 | 0.055291 |
| k__*Bacteria;* p__*Myxococcota;* c__*unidentified_Myxococcota* | 4.018513 | CRAB | 3.795314 | 0.242692 | 0 | 0.018693 | 0 | 0.038029 | 0 | 0 | 0.003599 | 0 | 0 | 0 | 0 |
| k__*Bacteria;* p__*Nitrospirota;* c__*Nitrospiria* | 2.701854 | CRAB | 2.519771 | 0.540291 | 0 | 0.003521 | 0 | 0 | 0 | 0 | 0 | 0 | 0 | 0 | 0 |
| k__*Bacteria;* p__*Planctomycetes;* c__*unidentified_Planctomycetes* | 3.405542 |  |  |  | 0 | 0.011496 | 0.003699 | 0 | 0 | 0 | 0 | 0 | 0 | 0.001748 | 0 |
| k__*Bacteria;* p__*Planctomycetota;* c__*Phycisphaerae* | 2.75258 | CRAB | 2.52218 | 0.540291 | 0 | 0.003957 | 0 | 0 | 0 | 0 | 0 | 0 | 0 | 0 | 0 |
| k__*Bacteria;* p__*Proteobacteria;* c__*Alphaproteobacteria* | 5.229612 | CRAB | 4.76649 | 0.307434 | 0.030488 | 0.206156 | 0.181052 | 0.152885 | 0.108144 | 0.079544 | 0.101878 | 0.02944 | 0.068756 | 0.111763 | 0.001274 |
| k__*Bacteria;* p__*Proteobacteria;* c__*Gammaproteobacteria* | 5.251577 | CRAB | 4.434491 | 0.683091 | 0.153684 | 0.235661 | 0 | 0.191388 | 0.061073 | 0.076519 | 0.274759 | 0.049165 | 0.118803 | 0.09357 | 0.064654 |
| k__*Bacteria;* p__*Spirochaetota;* c__*Spirochaetia* | 3.513819 | CRAB | 3.188683 | 0.540291 | 0 | 0 | 0 | 0 | 0 | 0 | 0 | 0.018452 | 0 | 0 | 0 |
| k__*Bacteria;* p__*Thermotogota;* c__*Thermotogae* | 3.252085 | CRAB | 2.970365 | 0.15332 | 0 | 0 | 0 | 0 | 0 | 0 | 0 | 0.0101 | 0.002283 | 0 | 5.93E-05 |
| k__*Bacteria;* p__*unidentified_Bacteria;* c__*Anaerolineae* | 2.641077 | CRAB | 2.225767 | 0.540291 | 0 | 0 | 0 | 0 | 0 | 0.003381 | 0 | 0 | 0 | 0 | 0 |
| k__*Bacteria;* p__*unidentified_Bacteria;* c__*Ktedonobacteria* | 3.223964 | CRAB | 3.084394 | 0.544827 | 0 | 0.011714 | 0 | 0 | 0 | 0 | 0 | 0 | 0.002283 | 0 | 0 |
| k__*Bacteria;* p__*unidentified_Bacteria;* c__*unidentified_Bacteria* | 3.741815 | CRAB | 3.389556 | 0.242692 | 0 | 0.001994 | 0.010001 | 0 | 0 | 0.021621 | 0 | 0 | 0 | 0 | 0 |
| k__*Bacteria;* p__*Verrucomicrobiota;* c__*Verrucomicrobiae* | 3.839314 |  |  |  | 0 | 0.037449 | 0 | 0.004676 | 0 | 0 | 0.004838 | 0 | 0 | 0.005659 | 0 |
| k__*Bacteria;* p__*Acidobacteriota;* c__*Acidobacteriae;* o__*Acidobacteriales* | 4.306277 | CRAB | 4.073536 | 0.363722 | 0 | 0.071595 | 0 | 0 | 0 | 0 | 0.053403 | 0 | 0 | 0 | 0 |
| k__*Bacteria;* p__*Acidobacteriota;* c__*Acidobacteriae;* o__*Bryobacterales* | 4.125455 | CRAB | 3.953207 | 0.363722 | 0 | 0.016357 | 0 | 0 | 0 | 0 | 0.071971 | 0 | 0 | 0 | 0 |
| k__*Bacteria;* p__*Acidobacteriota;* c__*Acidobacteriae;* o__*Solibacterales* | 4.600952 | CREC | 4.172067 | 0.363722 | 0 | 0.009721 | 0 | 0 | 0 | 0 | 0 | 0 | 0 | 0 | 0.057068 |
| k__*Bacteria;* p__*Acidobacteriota;* c__*Blastocatellia;* o__*unidentified_Blastocatellia* | 2.854323 | CRAB | 2.563229 | 0.540291 | 0 | 0.004113 | 0 | 0 | 0 | 0 | 0 | 0 | 0 | 0 | 0 |
| k__*Bacteria;* p__*Acidobacteriota;* c__*Thermoanaerobaculia;* o__*Thermoanaerobaculales* | 2.782968 | CRAB | 2.45553 | 0.540291 | 0 | 0.003489 | 0 | 0 | 0 | 0 | 0 | 0 | 0 | 0 | 0 |
| k__*Bacteria;* p__*Acidobacteriota;* c__*unidentified_Acidobacteriota;* o__*unidentified_Acidobacteriota* | 3.748185 | CRAB | 3.497066 | 0.363722 | 0 | 0.020937 | 0 | 0 | 0 | 0 | 0.013426 | 0 | 0 | 0 | 0 |
| k__*Bacteria;* p__*Acidobacteriota;* c__*Vicinamibacteria;* o__*Vicinamibacterales* | 4.222597 | CRAB | 4.025983 | 0.242692 | 0 | 0.010718 | 0 | 0.063362 | 0 | 0 | 0.018597 | 0 | 0 | 0 | 0 |
| k__*Bacteria;* p__*Actinobacteria;* c__*unidentified_Actinobacteria;* o__*Actinomycetales* | 1.967051 | CRAB | 1.822429 | 0.540291 | 0 | 0 | 0 | 0 | 0 | 0 | 0.000635 | 0 | 0 | 0 | 0 |
| k__*Bacteria;* p__*Actinobacteria;* c__*unidentified_Actinobacteria;* o__*Catenulisporales* | 2.076172 | CRAB | 1.794394 | 0.540291 | 0 | 0.000685 | 0 | 0 | 0 | 0 | 0 | 0 | 0 | 0 | 0 |
| k__*Bacteria;* p__*Actinobacteria;* c__*unidentified_Actinobacteria;* o__*Corynebacteriales* | 3.927391 | CRAB | 3.623496 | 0.373257 | 0.017333 | 0 | 0 | 0.017609 | 0 | 0.003945 | 0.003659 | 0 | 0.003083 | 0 | 0 |
| k__*Bacteria;* p__*Actinobacteria;* c__*unidentified_Actinobacteria;* o__*Frankiales* | 2.729377 |  |  |  | 0 | 0.000935 | 0 | 0.001539 | 0 | 0 | 0.000544 | 0 | 0.001127 | 0 | 0 |
| k__*Bacteria;* p__*Actinobacteria;* c__*unidentified_Actinobacteria;* o__*Micrococcales* | 4.631776 | CREC | 4.229067 | 0.753983 | 0.072916 | 0.004237 | 0 | 0 | 0.017851 | 0.018003 | 0.003357 | 0 | 0.007768 | 0.081156 | 0 |
| k__*Bacteria;* p__*Actinobacteria;* c__*unidentified_Actinobacteria;* o__*Micromonosporales* | 2.945368 | CREC | 2.667018 | 0.10247 | 0 | 0 | 0 | 0 | 0 | 0 | 0 | 0 | 0 | 0.001807 | 0 |
| k__*Bacteria;* p__*Actinobacteria;* c__*unidentified_Actinobacteria;* o__*Propionibacteriales* | 4.587597 | CREC | 4.28833 | 0.29619 | 0.003111 | 0.005982 | 0 | 0 | 0 | 0.005457 | 0.000847 | 0 | 0.002698 | 0.076444 | 0.000385 |
| k__*Bacteria;* p__*Actinobacteria;* c__*unidentified_Actinobacteria;* o__*Pseudonocardiales* | 2.257616 | CRAB | 2.074712 | 0.540291 | 0 | 0 | 0 | 0 | 0 | 0 | 0.00124 | 0 | 0 | 0 | 0 |
| k__*Bacteria;* p__*Actinobacteria;* c__*unidentified_Actinobacteria;* o__*Streptomycetales* | 2.762098 | CREC | 2.345666 | 0.544827 | 0 | 0.002212 | 0 | 0 | 0 | 0 | 0 | 0 | 0 | 0.001185 | 0 |
| k__*Bacteria;* p__*Actinobacteria;* c__*unidentified_Actinobacteria;* o__*Streptosporangiales* | 2.55446 | CREC | 2.293658 | 0.10247 | 0 | 0 | 0 | 0 | 0 | 0 | 0 | 0 | 0.00086 | 0 | 0 |
| k__*Bacteria;* p__*Actinobacteriota;* c__*Acidimicrobiia;* o__*unidentified_Acidimicrobiia* | 3.388828 | CRAB | 3.173001 | 0.363722 | 0 | 0.006138 | 0 | 0.007221 | 0 | 0 | 0 | 0 | 0 | 0 | 0 |
| k__*Bacteria;* p__*Actinobacteriota;* c__*Coriobacteriia;* o__*Coriobacteriales* | 3.74816 | CREC | 3.482988 | 0.603595 | 0 | 0 | 0 | 0 | 0 | 0.015956 | 0 | 0.011343 | 0.013431 | 0 | 0 |
| k__*Bacteria;* p__*Actinobacteriota;* c__*Thermoleophilia;* o__*Gaiellales* | 3.985919 | CRAB | 3.734871 | 0.156039 | 0 | 0.030096 | 0 | 0.01823 | 0 | 0.005042 | 0.002087 | 0 | 0 | 0 | 0 |
| k__*Bacteria;* p__*Actinobacteriota;* c__*Thermoleophilia;* o__*Solirubrobacterales* | 4.751891 | CREC | 4.282133 | 0.220845 | 0.019614 | 0.006823 | 0 | 0 | 0 | 0 | 0.004929 | 0 | 0.024964 | 0 | 0.065898 |
| k__*Bacteria;* p__*Armatimonadota;* c__*Armatimonadia;* o__*Armatimonadales* | 2.658029 | CRAB | 2.353073 | 0.540291 | 0 | 0.002617 | 0 | 0 | 0 | 0 | 0 | 0 | 0 | 0 | 0 |
| k__*Bacteria;* p__*Armatimonadota;* c__*Fimbriimonadia;* o__*Fimbriimonadales* | 2.653432 | CRAB | 2.512959 | 0.540291 | 0 | 0 | 0 | 0 | 0 | 0 | 0.003084 | 0 | 0 | 0 | 0 |
| k__*Bacteria;* p__*Bacteroidota;* c__*Bacteroidia;* o__*Bacteroidales* | 4.759608 | CRAB | 4.193178 | 0.473952 | 0.007822 | 0 | 0.00287 | 0.020272 | 0.036976 | 0.106801 | 0.004687 | 0.144118 | 0.046934 | 0 | 0.006163 |
| k__*Bacteria;* p__*Bacteroidota;* c__*Bacteroidia;* o__*Chitinophagales* | 3.624888 | CRAB | 3.388067 | 0.363722 | 0 | 0.009752 | 0 | 0 | 0 | 0 | 0.017267 | 0 | 0 | 0 | 0 |
| k__*Bacteria;* p__*Bacteroidota;* c__*Bacteroidia;* o__*Cytophagales* | 4.702489 | CREC | 4.315819 | 0.097937 | 0 | 0.003708 | 0 | 0 | 0 | 0 | 0.003871 | 0 | 0 | 0.030074 | 0.051113 |
| k__*Bacteria;* p__*Bacteroidota;* c__*Bacteroidia;* o__*Flavobacteriales* | 3.944626 | CRAB | 3.58037 | 0.156039 | 0 | 0.000592 | 0 | 0 | 0 | 0.038882 | 0.001935 | 0.018925 | 0 | 0 | 0 |
| k__*Bacteria;* p__*Bacteroidota;* c__*Bacteroidia;* o__*Sphingobacteriales* | 2.909841 | CRAB | 2.601834 | 0.540291 | 0 | 0.004673 | 0 | 0 | 0 | 0 | 0 | 0 | 0 | 0 | 0 |
| k__*Bacteria;* p__*Cyanobacteria;* c__*Sericytochromatia;* o__*unidentified_Sericytochromatia* | 2.076172 | CRAB | 1.757081 | 0.540291 | 0 | 0.000685 | 0 | 0 | 0 | 0 | 0 | 0 | 0 | 0 | 0 |
| k__*Bacteria;* p__*Cyanobacteria;* c__*unidentified_Cyanobacteria;* o__*Leptolyngbyales* | 3.579201 | CREC | 3.343052 | 0.10247 | 0 | 0 | 0 | 0 | 0 | 0 | 0 | 0 | 0.009102 | 0 | 0 |
| k__*Bacteria;* p__*Cyanobacteria;* c__*unidentified_Cyanobacteria;* o__*Synechococcales* | 3.011587 | CRAB | 2.546893 | 0.540291 | 0 | 0 | 0 | 0 | 0 | 0 | 0 | 0.005805 | 0 | 0 | 0 |
| k__*Bacteria;* p__*Cyanobacteria;* c__*unidentified_Cyanobacteria;* o__*unidentified_Cyanobacteria* | 3.914884 | CREC | 3.654508 | 0.10247 | 0 | 0 | 0 | 0 | 0 | 0 | 0 | 0 | 0.019717 | 0 | 0 |
| k__*Bacteria;* p__*Deinococcota;* c__*Deinococci;* o__*Deinococcales* | 4.246486 | CREC | 3.992186 | 0.363722 | 0 | 0 | 0 | 0 | 0 | 0.019248 | 0 | 0 | 0.042309 | 0 | 0 |
| k__*Bacteria;* p__*Desulfobacterota;* c__*Desulfovibrionia;* o__*Desulfovibrionales* | 2.451724 | CRAB | 1.967152 | 0.540291 | 0 | 0 | 0 | 0 | 0 | 0 | 0 | 0.001599 | 0 | 0 | 0 |
| k__*Bacteria;* p__*Desulfobacterota;* c__*Syntrophia;* o__*Syntrophales* | 3.95473 | CRAB | 3.276575 | 0.540291 | 0 | 0 | 0 | 0 | 0.030611 | 0 | 0 | 0 | 0 | 0 | 0 |
| k__*Bacteria;* p__*Desulfobacterota;* c__*Syntrophorhabdia;* o__*Syntrophorhabdales* | 3.542805 | CRAB | 3.083462 | 0.540291 | 0 | 0 | 0 | 0 | 0 | 0 | 0 | 0.019725 | 0 | 0 | 0 |
| k__*Bacteria;* p__*Firmicutes;* c__*Bacilli;* o__*Bacillales* | 4.834336 | CREC | 4.454629 | 0.464628 | 0 | 0.001464 | 0.022398 | 0 | 0 | 0.088798 | 0.002691 | 0.087638 | 0.123014 | 0.034844 | 0 |
| k__*Bacteria;* p__*Firmicutes;* c__*Bacilli;* o__*Erysipelotrichales* | 4.058186 |  |  |  | 0 | 0 | 0 | 0 | 0 | 0.025121 | 0 | 0.046114 | 0.012986 | 0 | 0 |
| k__*Bacteria;* p__*Firmicutes;* c__*Bacilli;* o__*Lactobacillales* | 5.028112 | CREC | 4.351295 | 0.149291 | 0 | 0 | 0.02154 | 0.038118 | 0 | 0.086158 | 0.273882 | 0.057132 | 0.096656 | 0.092089 | 0.030697 |
| k__*Bacteria;* p__*Firmicutes;* c__*Bacilli;* o__*unidentified_Bacilli* | 4.95203 | CRAB | 4.530715 | 0.095022 | 0.043376 | 0 | 0.104773 | 0 | 0.156281 | 0.022244 | 0 | 0.007286 | 0 | 0 | 0 |
| k__*Bacteria;* p__*Firmicutes;* c__*Clostridia;* o__*Christensenellales* | 3.766004 | CREC | 3.515871 | 0.10247 | 0 | 0 | 0 | 0 | 0 | 0 | 0 | 0 | 0.013994 | 0 | 0 |
| k__*Bacteria;* p__*Firmicutes;* c__*Clostridia;* o__*Clostridiales* | 4.864759 | CRAB | 4.155246 | 0.836787 | 0.036947 | 3.12E-05 | 0.133947 | 0.041344 | 0 | 0.034819 | 0 | 0.083639 | 0.080793 | 0.063378 | 0 |
| k__*Bacteria;* p__*Firmicutes;* c__*Clostridia;* o__*Lachnospirales* | 4.881508 | CRAB | 4.433222 | 0.680331 | 0.124944 | 0.001122 | 0.033109 | 0 | 0 | 0.20328 | 0.00626 | 0.057191 | 0.06253 | 0 | 0.006845 |
| k__*Bacteria;* p__*Firmicutes;* c__*Clostridia;* o__*unidentified_Clostridia* | 5.099947 | CREC | 4.558944 | 0.21962 | 0.025451 | 0 | 0 | 0.058952 | 0.013736 | 0.027256 | 0.000877 | 0.04022 | 0.034037 | 0.032119 | 0.137337 |
| k__*Bacteria;* p__*Firmicutes;* c__*Negativicutes;* o__*unidentified_Negativicutes* | 4.123816 |  |  |  | 0 | 0 | 0 | 0 | 0 | 0.076727 | 0 | 0.018629 | 0.011385 | 0 | 0 |
| k__*Bacteria;* p__*Gemmatimonadota;* c__*Gemmatimonadetes;* o__*Gemmatimonadales* | 4.447168 | CREC | 4.204561 | 0.603595 | 0 | 0.021653 | 0 | 0 | 0 | 0 | 0.003659 | 0 | 0 | 0.057393 | 0 |
| k__*Bacteria;* p__*Myxococcota;* c__*Myxococcia;* o__*Myxococcales* | 4.587207 | CREC | 4.140884 | 0.603595 | 0 | 0.008443 | 0 | 0 | 0 | 0 | 0.003175 | 0 | 0 | 0 | 0.055291 |
| k__*Bacteria;* p__*Myxococcota;* c__*unidentified_Myxococcota;* o__*Haliangiales* | 3.136816 | CRAB | 2.892491 | 0.363722 | 0 | 0.00486 | 0 | 0 | 0 | 0 | 0.003599 | 0 | 0 | 0 | 0 |
| k__*Bacteria;* p__*Myxococcota;* c__*unidentified_Myxococcota;* o__*Polyangiales* | 3.985777 | CRAB | 3.814417 | 0.363722 | 0 | 0.013833 | 0 | 0.038029 | 0 | 0 | 0 | 0 | 0 | 0 | 0 |
| k__*Bacteria;* p__*Nitrospirota;* c__*Nitrospiria;* o__*Nitrospirales* | 2.786828 | CRAB | 2.462227 | 0.540291 | 0 | 0.003521 | 0 | 0 | 0 | 0 | 0 | 0 | 0 | 0 | 0 |
| k__*Bacteria;* p__*Planctomycetes;* c__*unidentified_Planctomycetes;* o__*Gemmatales* | 3.450167 |  |  |  | 0 | 0.010281 | 0.003699 | 0 | 0 | 0 | 0 | 0 | 0 | 0.001748 | 0 |
| k__*Bacteria;* p__*Planctomycetes;* c__*unidentified_Planctomycetes;* o__*Isosphaerales* | 2.324814 | CRAB | 2.019675 | 0.540291 | 0 | 0.001215 | 0 | 0 | 0 | 0 | 0 | 0 | 0 | 0 | 0 |
| k__*Bacteria;* p__*Planctomycetota;* c__*Phycisphaerae;* o__*Tepidisphaerales* | 2.837553 | CRAB | 2.530906 | 0.540291 | 0 | 0.003957 | 0 | 0 | 0 | 0 | 0 | 0 | 0 | 0 | 0 |
| k__*Bacteria;* p__*Proteobacteria;* c__*Alphaproteobacteria;* o__*Caulobacterales* | 4.243989 | CRAB | 4.042879 | 0.373257 | 0.007615 | 0.013553 | 0 | 0.064279 | 0 | 0 | 0.00762 | 0 | 0 | 0 | 8.89E-05 |
| k__*Bacteria;* p__*Proteobacteria;* c__*Alphaproteobacteria;* o__*Micropepsales* | 3.526249 | CRAB | 3.258776 | 0.363722 | 0 | 0.01564 | 0 | 0 | 0 | 0 | 0.004385 | 0 | 0 | 0 | 0 |
| k__*Bacteria;* p__*Proteobacteria;* c__*Alphaproteobacteria;* o__*Rhizobiales* | 4.936135 | CRAB | 4.4106 | 0.216446 | 0 | 0.110571 | 0.115691 | 0.01826 | 0.069333 | 0.014651 | 0.048353 | 0.011047 | 0 | 0.081244 | 0 |
| k__*Bacteria;* p__*Proteobacteria;* c__*Alphaproteobacteria;* o__*Rhodobacterales* | 3.489155 | CRAB | 3.289595 | 0.237166 | 0 | 0 | 0 | 0 | 0 | 0.022541 | 0.000998 | 0 | 0.003795 | 0 | 0.000593 |
| k__*Bacteria;* p__*Proteobacteria;* c__*Alphaproteobacteria;* o__*Rhodospirillales* | 3.488003 | CRAB | 3.308428 | 0.363722 | 0 | 0.003552 | 0 | 0 | 0 | 0 | 0.016844 | 0 | 0 | 0 | 0 |
| k__*Bacteria;* p__*Proteobacteria;* c__*Alphaproteobacteria;* o__*Rickettsiales* | 4.435768 | CRAB | 3.733848 | 0.838256 | 0.022873 | 0.00835 | 0 | 0.022965 | 0.038811 | 0.010796 | 6.05E-05 | 0.018392 | 0.019628 | 0.030519 | 0.000593 |
| k__*Bacteria;* p__*Proteobacteria;* c__*Alphaproteobacteria;* o__*Sphingomonadales* | 4.27647 | CREC | 3.843435 | 0.823849 | 0 | 0.009129 | 0 | 0.047381 | 0 | 0.030311 | 0.000816 | 0 | 0.045333 | 0 | 0 |
| k__*Bacteria;* p__*Proteobacteria;* c__*Alphaproteobacteria;* o__*unidentified_Alphaproteobacteria* | 3.386157 | CRAB | 3.06244 | 0.242692 | 0 | 0.0124 | 0 | 0 | 0 | 0.001246 | 0.000786 | 0 | 0 | 0 | 0 |
| k__*Bacteria;* p__*Proteobacteria;* c__*Gammaproteobacteria;* o__*Burkholderiales* | 5.114456 | CRAB | 4.476195 | 0.540291 | 0.151314 | 0.165966 | 0 | 0.146552 | 0.061073 | 0.040128 | 0.052617 | 0.039983 | 0.094877 | 0.062815 | 0.00157 |
| k__*Bacteria;* p__*Proteobacteria;* c__*Gammaproteobacteria;* o__*Enterobacterales* | 4.528008 | CRAB | 4.278094 | 0.152107 | 0.00237 | 0.001807 | 0 | 0.001391 | 0 | 0.003203 | 0.212374 | 0.006664 | 0.013787 | 0.0024 | 0.013897 |
| k__*Bacteria;* p__*Proteobacteria;* c__*Gammaproteobacteria;* o__*Pseudomonadales* | 4.393986 | CREC | 3.765786 | 0.216446 | 0 | 0.011278 | 0 | 0.043445 | 0 | 0.033188 | 0.005806 | 0.002517 | 0.01014 | 0.028356 | 0.0096 |
| k__*Bacteria;* p__*Proteobacteria;* c__*Gammaproteobacteria;* o__*Steroidobacterales* | 2.882972 | CRAB | 2.633278 | 0.363722 | 0 | 0.003708 | 0 | 0 | 0 | 0 | 0.000816 | 0 | 0 | 0 | 0 |
| k__*Bacteria;* p__*Proteobacteria;* c__*Gammaproteobacteria;* o__*unidentified_Gammaproteobacteria* | 4.442102 | CREC | 4.017569 | 0.363722 | 0 | 0.02103 | 0 | 0 | 0 | 0 | 0 | 0 | 0 | 0 | 0.039586 |
| k__*Bacteria;* p__*Proteobacteria;* c__*Gammaproteobacteria;* o__*Xanthomonadales* | 3.658414 | CRAB | 3.359516 | 0.363722 | 0 | 0.023554 | 0 | 0 | 0 | 0 | 0.003145 | 0 | 0 | 0 | 0 |
| k__*Bacteria;* p__*Spirochaetota;* c__*Spirochaetia;* o__*Spirochaetales* | 3.513819 | CRAB | 2.977859 | 0.540291 | 0 | 0 | 0 | 0 | 0 | 0 | 0 | 0.018452 | 0 | 0 | 0 |
| k__*Bacteria;* p__*Thermotogota;* c__*Thermotogae;* o__*Petrotogales* | 3.252085 | CRAB | 2.957586 | 0.15332 | 0 | 0 | 0 | 0 | 0 | 0 | 0 | 0.0101 | 0.002283 | 0 | 5.93E-05 |
| k__*Bacteria;* p__*unidentified_Bacteria;* c__*Anaerolineae;* o__*Ardenticatenales* | 2.644236 | CRAB | 2.358319 | 0.540291 | 0 | 0 | 0 | 0 | 0 | 0.003381 | 0 | 0 | 0 | 0 | 0 |
| k__*Bacteria;* p__*unidentified_Bacteria;* c__*Ktedonobacteria;* o__*Ktedonobacterales* | 3.308937 | CRAB | 2.986611 | 0.544827 | 0 | 0.011714 | 0 | 0 | 0 | 0 | 0 | 0 | 0.002283 | 0 | 0 |
| k__*Bacteria;* p__*unidentified_Bacteria;* c__*unidentified_Bacteria;* o__*Saccharimonadales* | 3.774933 | CRAB | 3.493147 | 0.242692 | 0 | 0.001994 | 0.010001 | 0 | 0 | 0.021621 | 0 | 0 | 0 | 0 | 0 |
| k__*Bacteria;* p__*Verrucomicrobiota;* c__*Verrucomicrobiae;* o__*Chthoniobacterales* | 3.58592 |  |  |  | 0 | 0.015297 | 0 | 0.004676 | 0 | 0 | 0.002056 | 0 | 0 | 0.005659 | 0 |
| k__*Bacteria;* p__*Verrucomicrobiota;* c__*Verrucomicrobiae;* o__*Methylacidiphilales* | 2.196147 | CRAB | 1.920751 | 0.540291 | 0 | 0.000904 | 0 | 0 | 0 | 0 | 0 | 0 | 0 | 0 | 0 |
| k__*Bacteria;* p__*Verrucomicrobiota;* c__*Verrucomicrobiae;* o__*unidentified_Verrucomicrobiae* | 2.553293 | CRAB | 2.223496 | 0.540291 | 0 | 0.002056 | 0 | 0 | 0 | 0 | 0 | 0 | 0 | 0 | 0 |
| k__*Bacteria;* p__*Acidobacteriota;* c__*Acidobacteriae;* o__*Acidobacteriales;* f__*unidentified_Acidobacteriales* | 3.365541 | CRAB | 3.173533 | 0.540291 | 0 | 0.010531 | 0 | 0 | 0 | 0 | 0 | 0 | 0 | 0 | 0 |
| k__*Bacteria;* p__*Acidobacteriota;* c__*Acidobacteriae;* o__*Bryobacterales;* f__*Bryobacteraceae* | 4.186617 | CRAB | 3.908559 | 0.363722 | 0 | 0.016357 | 0 | 0 | 0 | 0 | 0.071971 | 0 | 0 | 0 | 0 |
| k__*Bacteria;* p__*Acidobacteriota;* c__*Acidobacteriae;* o__*Solibacterales;* f__*Solibacteraceae* | 4.667845 | CREC | 4.295811 | 0.363722 | 0 | 0.009721 | 0 | 0 | 0 | 0 | 0 | 0 | 0 | 0 | 0.057068 |
| k__*Bacteria;* p__*Acidobacteriota;* c__*Blastocatellia;* o__*unidentified_Blastocatellia;* f__*Pyrinomonadaceae* | 2.957198 | CRAB | 2.755853 | 0.540291 | 0 | 0.004113 | 0 | 0 | 0 | 0 | 0 | 0 | 0 | 0 | 0 |
| k__*Bacteria;* p__*Acidobacteriota;* c__*Thermoanaerobaculia;* o__*Thermoanaerobaculales;* f__*Thermoanaerobaculaceae* | 2.885842 | CRAB | 2.648035 | 0.540291 | 0 | 0.003489 | 0 | 0 | 0 | 0 | 0 | 0 | 0 | 0 | 0 |
| k__*Bacteria;* p__*Acidobacteriota;* c__*unidentified_Acidobacteriota;* o__*unidentified_Acidobacteriota;* f__*unidentified_Acidobacteriota* | 3.833003 | CRAB | 3.590746 | 0.363722 | 0 | 0.020937 | 0 | 0 | 0 | 0 | 0.013426 | 0 | 0 | 0 | 0 |
| k__*Bacteria;* p__*Acidobacteriota;* c__*Vicinamibacteria;* o__*Vicinamibacterales;* f__*unidentified_Vicinamibacterales* | 3.030674 | CRAB | 2.707977 | 0.540291 | 0 | 0 | 0 | 0.004913 | 0 | 0 | 0 | 0 | 0 | 0 | 0 |
| k__*Bacteria;* p__*Actinobacteria;* c__*unidentified_Actinobacteria;* o__*Actinomycetales;* f__*Actinomycetaceae* | 2.016198 | CRAB | 1.715993 | 0.540291 | 0 | 0 | 0 | 0 | 0 | 0 | 0.000635 | 0 | 0 | 0 | 0 |
| k__*Bacteria;* p__*Actinobacteria;* c__*unidentified_Actinobacteria;* o__*Catenulisporales;* f__*Catenulisporaceae* | 2.179047 | CRAB | 1.984931 | 0.540291 | 0 | 0.000685 | 0 | 0 | 0 | 0 | 0 | 0 | 0 | 0 | 0 |
| k__*Bacteria;* p__*Actinobacteria;* c__*unidentified_Actinobacteria;* o__*Corynebacteriales;* f__*Corynebacteriaceae* | 3.851502 | CRAB | 3.441079 | 0.373257 | 0.017333 | 0 | 0 | 0.00802 | 0 | 0.003945 | 0.003659 | 0 | 0.003083 | 0 | 0 |
| k__*Bacteria;* p__*Actinobacteria;* c__*unidentified_Actinobacteria;* o__*Corynebacteriales;* f__*Mycobacteriaceae* | 3.321111 | CRAB | 3.010531 | 0.540291 | 0 | 0 | 0 | 0.009589 | 0 | 0 | 0 | 0 | 0 | 0 | 0 |
| k__*Bacteria;* p__*Actinobacteria;* c__*unidentified_Actinobacteria;* o__*Frankiales;* f__*Acidothermaceae* | 2.234564 | CRAB | 1.997386 | 0.540291 | 0 | 0.000779 | 0 | 0 | 0 | 0 | 0 | 0 | 0 | 0 | 0 |
| k__*Bacteria;* p__*Actinobacteria;* c__*unidentified_Actinobacteria;* o__*Frankiales;* f__*Geodermatophilaceae* | 2.66226 | CRAB | 2.37557 | 0.242692 | 0 | 0.000156 | 0 | 0.001539 | 0 | 0 | 0.000544 | 0 | 0 | 0 | 0 |
| k__*Bacteria;* p__*Actinobacteria;* c__*unidentified_Actinobacteria;* o__*Micrococcales;* f__*Cellulomonadaceae* | 3.612853 | CREC | 3.327479 | 0.10247 | 0 | 0 | 0 | 0 | 0 | 0 | 0 | 0 | 0 | 0.00803 | 0 |
| k__*Bacteria;* p__*Actinobacteria;* c__*unidentified_Actinobacteria;* o__*Micrococcales;* f__*Intrasporangiaceae* | 4.252869 | CREC | 3.97736 | 0.603595 | 0 | 0.003084 | 0 | 0 | 0 | 0 | 0.002389 | 0 | 0 | 0.035052 | 0 |
| k__*Bacteria;* p__*Actinobacteria;* c__*unidentified_Actinobacteria;* o__*Micrococcales;* f__*Microbacteriaceae* | 3.638966 | CREC | 3.216706 | 0.237166 | 0 | 0.001153 | 0 | 0 | 0 | 0.010558 | 0 | 0 | 0.007768 | 0.001867 | 0 |
| k__*Bacteria;* p__*Actinobacteria;* c__*unidentified_Actinobacteria;* o__*Micrococcales;* f__*Micrococcaceae* | 4.392925 | CRAB | 4.017369 | 0.823849 | 0.072916 | 0 | 0 | 0 | 0.017851 | 0.007444 | 0.000968 | 0 | 0 | 0.036207 | 0 |
| k__*Bacteria;* p__*Actinobacteria;* c__*unidentified_Actinobacteria;* o__*Micromonosporales;* f__*Micromonosporaceae* | 2.965214 | CREC | 2.665608 | 0.10247 | 0 | 0 | 0 | 0 | 0 | 0 | 0 | 0 | 0 | 0.001807 | 0 |
| k__*Bacteria;* p__*Actinobacteria;* c__*unidentified_Actinobacteria;* o__*Propionibacteriales;* f__*Nocardioidaceae* | 3.10633 | CRAB | 2.772918 | 0.91138 | 0.003052 | 0.001838 | 0 | 0 | 0 | 0 | 0.000786 | 0 | 0 | 2.96E-05 | 0.000267 |
| k__*Bacteria;* p__*Actinobacteria;* c__*unidentified_Actinobacteria;* o__*Propionibacteriales;* f__*Propionibacteriaceae* | 4.605331 | CREC | 4.272843 | 0.094638 | 5.93E-05 | 0.004144 | 0 | 0 | 0 | 0.005457 | 6.05E-05 | 0 | 0.002698 | 0.076415 | 0.000119 |
| k__*Bacteria;* p__*Actinobacteria;* c__*unidentified_Actinobacteria;* o__*Pseudonocardiales;* f__*Pseudonocardiaceae* | 2.306762 | CRAB | 1.983934 | 0.540291 | 0 | 0 | 0 | 0 | 0 | 0 | 0.00124 | 0 | 0 | 0 | 0 |
| k__*Bacteria;* p__*Actinobacteria;* c__*unidentified_Actinobacteria;* o__*Streptomycetales;* f__*Streptomycetaceae* | 2.781944 | CREC | 2.445292 | 0.544827 | 0 | 0.002212 | 0 | 0 | 0 | 0 | 0 | 0 | 0 | 0.001185 | 0 |
| k__*Bacteria;* p__*Actinobacteria;* c__*unidentified_Actinobacteria;* o__*Streptosporangiales;* f__*Nocardiopsaceae* | 2.575766 | CREC | 2.333332 | 0.10247 | 0 | 0 | 0 | 0 | 0 | 0 | 0 | 0 | 0.00086 | 0 | 0 |
| k__*Bacteria;* p__*Actinobacteriota;* c__*Acidimicrobiia;* o__*unidentified_Acidimicrobiia;* f__*Iamiaceae* | 2.470092 | CRAB | 2.272627 | 0.540291 | 0 | 0.00134 | 0 | 0 | 0 | 0 | 0 | 0 | 0 | 0 | 0 |
| k__*Bacteria;* p__*Actinobacteriota;* c__*Acidimicrobiia;* o__*unidentified_Acidimicrobiia;* f__*unidentified_Acidimicrobiia* | 3.420718 | CRAB | 3.16318 | 0.363722 | 0 | 0.004798 | 0 | 0.007221 | 0 | 0 | 0 | 0 | 0 | 0 | 0 |
| k__*Bacteria;* p__*Actinobacteriota;* c__*Coriobacteriia;* o__*Coriobacteriales;* f__*Atopobiaceae* | 3.537143 | CRAB | 3.297098 | 0.363722 | 0 | 0 | 0 | 0 | 0 | 0.010321 | 0 | 0.011343 | 0 | 0 | 0 |
| k__*Bacteria;* p__*Actinobacteriota;* c__*Coriobacteriia;* o__*Coriobacteriales;* f__*Eggerthellaceae* | 3.769466 | CREC | 3.44217 | 0.363722 | 0 | 0 | 0 | 0 | 0 | 0.005635 | 0 | 0 | 0.013431 | 0 | 0 |
| k__*Bacteria;* p__*Actinobacteriota;* c__*Thermoleophilia;* o__*Gaiellales;* f__*Gaiellaceae* | 3.629525 | CRAB | 3.300923 | 0.242692 | 0 | 0.000592 | 0 | 0.01823 | 0 | 0 | 0.000907 | 0 | 0 | 0 | 0 |
| k__*Bacteria;* p__*Actinobacteriota;* c__*Thermoleophilia;* o__*Solirubrobacterales;* f__*Solirubrobacteraceae* | 3.850652 |  |  |  | 0.019614 | 0.006823 | 0 | 0 | 0 | 0 | 0.004929 | 0 | 0.012304 | 0 | 0 |
| k__*Bacteria;* p__*Armatimonadota;* c__*Fimbriimonadia;* o__*Fimbriimonadales;* f__*Fimbriimonadaceae* | 2.702579 | CRAB | 2.374286 | 0.540291 | 0 | 0 | 0 | 0 | 0 | 0 | 0.003084 | 0 | 0 | 0 | 0 |
| k__*Bacteria;* p__*Bacteroidota;* c__*Bacteroidia;* o__*Bacteroidales;* f__*Bacteroidaceae* | 3.933634 | CRAB | 3.659676 | 0.242692 | 0 | 0 | 0.00287 | 0 | 0 | 0.009461 | 0 | 0.034919 | 0 | 0 | 0 |
| k__*Bacteria;* p__*Bacteroidota;* c__*Bacteroidia;* o__*Bacteroidales;* f__*Marinifilaceae* | 3.267344 | CRAB | 3.010658 | 0.540291 | 0 | 0 | 0 | 0 | 0 | 0 | 0 | 0.010188 | 0 | 0 | 0 |
| k__*Bacteria;* p__*Bacteroidota;* c__*Bacteroidia;* o__*Bacteroidales;* f__*Muribaculaceae* | 4.166696 | CRAB | 3.872764 | 0.636328 | 0.007822 | 0 | 0 | 0 | 0 | 0.057211 | 0 | 0.028077 | 0.003528 | 0 | 0 |
| k__*Bacteria;* p__*Bacteroidota;* c__*Bacteroidia;* o__*Bacteroidales;* f__*Porphyromonadaceae* | 3.701079 | CREC | 3.440092 | 0.10247 | 0 | 0 | 0 | 0 | 0 | 0 | 0 | 0 | 0.011474 | 0 | 0 |
| k__*Bacteria;* p__*Bacteroidota;* c__*Bacteroidia;* o__*Bacteroidales;* f__*Prevotellaceae* | 3.961301 | CREC | 3.585744 | 0.435926 | 0 | 0 | 0 | 0.009115 | 0 | 0.023282 | 0 | 0.014927 | 0.020725 | 0 | 8.89E-05 |
| k__*Bacteria;* p__*Bacteroidota;* c__*Bacteroidia;* o__*Bacteroidales;* f__*Rikenellaceae* | 4.407457 | CRAB | 3.875025 | 0.601448 | 0 | 0 | 0 | 0.011157 | 0.036976 | 0.016846 | 0.004687 | 0.044663 | 0.011207 | 0 | 0.006074 |
| k__*Bacteria;* p__*Bacteroidota;* c__*Bacteroidia;* o__*Bacteroidales;* f__*Tannerellaceae* | 3.313985 | CRAB | 3.036132 | 0.540291 | 0 | 0 | 0 | 0 | 0 | 0 | 0 | 0.011343 | 0 | 0 | 0 |
| k__*Bacteria;* p__*Bacteroidota;* c__*Bacteroidia;* o__*Chitinophagales;* f__*Chitinophagaceae* | 3.696447 | CRAB | 3.426024 | 0.363722 | 0 | 0.009752 | 0 | 0 | 0 | 0 | 0.017267 | 0 | 0 | 0 | 0 |
| k__*Bacteria;* p__*Bacteroidota;* c__*Bacteroidia;* o__*Cytophagales;* f__*Hymenobacteraceae* | 4.18635 | CREC | 3.876443 | 0.363722 | 0 | 0.000935 | 0 | 0 | 0 | 0 | 0 | 0 | 0 | 0.030074 | 0 |
| k__*Bacteria;* p__*Bacteroidota;* c__*Bacteroidia;* o__*Cytophagales;* f__*Microscillaceae* | 4.619978 | CREC | 4.248324 | 0.603595 | 0 | 0.002773 | 0 | 0 | 0 | 0 | 0.003871 | 0 | 0 | 0 | 0.051113 |
| k__*Bacteria;* p__*Bacteroidota;* c__*Bacteroidia;* o__*Flavobacteriales;* f__*Flavobacteriaceae* | 3.441363 | CRAB | 3.184343 | 0.156039 | 0 | 0.000592 | 0 | 0 | 0 | 0.005517 | 0.001935 | 0.008678 | 0 | 0 | 0 |
| k__*Bacteria;* p__*Bacteroidota;* c__*Bacteroidia;* o__*Flavobacteriales;* f__*Weeksellaceae* | 3.801806 | CRAB | 3.551783 | 0.363722 | 0 | 0 | 0 | 0 | 0 | 0.033366 | 0 | 0.010248 | 0 | 0 | 0 |
| k__*Bacteria;* p__*Bacteroidota;* c__*Bacteroidia;* o__*Sphingobacteriales;* f__*Sphingobacteriaceae* | 3.012715 | CRAB | 2.787035 | 0.540291 | 0 | 0.004673 | 0 | 0 | 0 | 0 | 0 | 0 | 0 | 0 | 0 |
| k__*Bacteria;* p__*Cyanobacteria;* c__*Sericytochromatia;* o__*unidentified_Sericytochromatia;* f__*unidentified_Sericytochromatia* | 2.179047 | CRAB | 1.951331 | 0.540291 | 0 | 0.000685 | 0 | 0 | 0 | 0 | 0 | 0 | 0 | 0 | 0 |
| k__*Bacteria;* p__*Cyanobacteria;* c__*unidentified_Cyanobacteria;* o__*Leptolyngbyales;* f__*Leptolyngbyaceae* | 3.600507 | CREC | 3.353095 | 0.10247 | 0 | 0 | 0 | 0 | 0 | 0 | 0 | 0 | 0.009102 | 0 | 0 |
| k__*Bacteria;* p__*Cyanobacteria;* c__*unidentified_Cyanobacteria;* o__*Synechococcales;* f__*Cyanobiaceae* | 3.023042 | CRAB | 2.773771 | 0.540291 | 0 | 0 | 0 | 0 | 0 | 0 | 0 | 0.005805 | 0 | 0 | 0 |
| k__*Bacteria;* p__*Cyanobacteria;* c__*unidentified_Cyanobacteria;* o__*unidentified_Cyanobacteria;* f__*Nostocaceae* | 3.93619 | CREC | 3.682113 | 0.10247 | 0 | 0 | 0 | 0 | 0 | 0 | 0 | 0 | 0.019717 | 0 | 0 |
| k__*Bacteria;* p__*Deinococcota;* c__*Deinococci;* o__*Deinococcales;* f__*Deinococcaceae* | 4.267792 | CREC | 3.931258 | 0.363722 | 0 | 0 | 0 | 0 | 0 | 0.019248 | 0 | 0 | 0.042309 | 0 | 0 |
| k__*Bacteria;* p__*Desulfobacterota;* c__*Desulfovibrionia;* o__*Desulfovibrionales;* f__*Desulfovibrionaceae* | 2.46318 | CRAB | 2.196216 | 0.540291 | 0 | 0 | 0 | 0 | 0 | 0 | 0 | 0.001599 | 0 | 0 | 0 |
| k__*Bacteria;* p__*Desulfobacterota;* c__*Syntrophia;* o__*Syntrophales;* f__*Smithellaceae* | 3.996353 | CRAB | 3.56934 | 0.540291 | 0 | 0 | 0 | 0 | 0.030611 | 0 | 0 | 0 | 0 | 0 | 0 |
| k__*Bacteria;* p__*Desulfobacterota;* c__*Syntrophorhabdia;* o__*Syntrophorhabdales;* f__*Syntrophorhabdaceae* | 3.55426 | CRAB | 3.284625 | 0.540291 | 0 | 0 | 0 | 0 | 0 | 0 | 0 | 0.019725 | 0 | 0 | 0 |
| k__*Bacteria;* p__*Firmicutes;* c__*Bacilli;* o__*Bacillales;* f__*Bacillaceae* | 4.855279 | CREC | 4.421375 | 0.316494 | 0 | 0 | 0 | 0 | 0 | 0.088798 | 0.002691 | 0.087638 | 0.123014 | 0.034844 | 0 |
| k__*Bacteria;* p__*Firmicutes;* c__*Bacilli;* o__*Bacillales;* f__*Planococcaceae* | 3.897689 | CRAB | 3.55039 | 0.363722 | 0 | 0.001464 | 0.022398 | 0 | 0 | 0 | 0 | 0 | 0 | 0 | 0 |
| k__*Bacteria;* p__*Firmicutes;* c__*Bacilli;* o__*Erysipelotrichales;* f__*Erysipelotrichaceae* | 4.069869 |  |  |  | 0 | 0 | 0 | 0 | 0 | 0.025121 | 0 | 0.046114 | 0.012156 | 0 | 0 |
| k__*Bacteria;* p__*Firmicutes;* c__*Bacilli;* o__*Lactobacillales;* f__*Aerococcaceae* | 4.390509 | CREC | 3.978394 | 0.10247 | 0 | 0 | 0 | 0 | 0 | 0 | 0 | 0 | 0 | 0 | 0.030134 |
| k__*Bacteria;* p__*Firmicutes;* c__*Bacilli;* o__*Lactobacillales;* f__*Enterococcaceae* | 4.723847 | CRAB | 4.388501 | 0.636328 | 0 | 0 | 0 | 0 | 0 | 0.031794 | 0.273882 | 0.021561 | 0.02369 | 0 | 0 |
| k__*Bacteria;* p__*Firmicutes;* c__*Bacilli;* o__*Lactobacillales;* f__*Lactobacillaceae* | 4.735869 | CREC | 4.25283 | 0.403318 | 0 | 0 | 0.02154 | 0.038118 | 0 | 0.042382 | 0 | 0.022183 | 0.015862 | 0.092089 | 0.000563 |
| k__*Bacteria;* p__*Firmicutes;* c__*Bacilli;* o__*Lactobacillales;* f__*Streptococcaceae* | 4.398025 | CREC | 4.09862 | 0.603595 | 0 | 0 | 0 | 0 | 0 | 0.011982 | 0 | 0.013387 | 0.057104 | 0 | 0 |
| k__*Bacteria;* p__*Firmicutes;* c__*Bacilli;* o__*unidentified_Bacilli;* f__*Staphylococcaceae* | 4.998403 | CRAB | 4.61514 | 0.156039 | 0.043376 | 0 | 0.104773 | 0 | 0.156281 | 0.022244 | 0 | 0 | 0 | 0 | 0 |
| k__*Bacteria;* p__*Firmicutes;* c__*Bacilli;* o__*unidentified_Bacilli;* f__*unidentified_Bacilli* | 3.121721 | CRAB | 2.857185 | 0.540291 | 0 | 0 | 0 | 0 | 0 | 0 | 0 | 0.007286 | 0 | 0 | 0 |
| k__*Bacteria;* p__*Firmicutes;* c__*Clostridia;* o__*Christensenellales;* f__*Christensenellaceae* | 3.78731 | CREC | 3.520207 | 0.10247 | 0 | 0 | 0 | 0 | 0 | 0 | 0 | 0 | 0.013994 | 0 | 0 |
| k__*Bacteria;* p__*Firmicutes;* c__*Clostridia;* o__*Clostridiales;* f__*Clostridiaceae* | 4.920268 | CRAB | 4.215136 | 0.836787 | 0.036947 | 3.12E-05 | 0.133947 | 0.041344 | 0 | 0.034819 | 0 | 0.083639 | 0.080793 | 0.063378 | 0 |
| k__*Bacteria;* p__*Firmicutes;* c__*Clostridia;* o__*Lachnospirales;* f__*Defluviitaleaceae* | 4.049317 | CRAB | 3.70574 | 0.540291 | 0 | 0 | 0.033109 | 0 | 0 | 0 | 0 | 0 | 0 | 0 | 0 |
| k__*Bacteria;* p__*Firmicutes;* c__*Clostridia;* o__*Lachnospirales;* f__*Lachnospiraceae* | 4.8412 | CRAB | 4.385094 | 0.916801 | 0.124944 | 0.001122 | 0 | 0 | 0 | 0.20328 | 0.00626 | 0.057191 | 0.06253 | 0 | 0.006845 |
| k__*Bacteria;* p__*Firmicutes;* c__*Clostridia;* o__*unidentified_Clostridia;* f__*Anaerovoracaceae* | 3.792656 | CRAB | 3.474218 | 0.540291 | 0.025451 | 0 | 0 | 0 | 0 | 0 | 0 | 0 | 0 | 0 | 0 |
| k__*Bacteria;* p__*Firmicutes;* c__*Clostridia;* o__*unidentified_Clostridia;* f__*Oscillospiraceae* | 3.842782 | CRAB | 3.49734 | 0.540291 | 0 | 0 | 0 | 0.031873 | 0 | 0 | 0 | 0 | 0 | 0 | 0 |
| k__*Bacteria;* p__*Firmicutes;* c__*Clostridia;* o__*unidentified_Clostridia;* f__*Peptostreptococcaceae* | 5.08521 | CREC | 4.687365 | 0.435926 | 0 | 0 | 0 | 0.027079 | 0.013736 | 0.027256 | 0 | 0 | 0.022088 | 0 | 0.137337 |
| k__*Bacteria;* p__*Firmicutes;* c__*Clostridia;* o__*unidentified_Clostridia;* f__*Ruminococcaceae* | 3.863686 | CRAB | 3.647773 | 0.544827 | 0 | 0 | 0 | 0 | 0 | 0 | 0 | 0.04022 | 0.011949 | 0 | 0 |
| k__*Bacteria;* p__*Firmicutes;* c__*Clostridia;* o__*unidentified_Clostridia;* f__*unidentified_Clostridia* | 4.214913 | CREC | 3.938324 | 0.363722 | 0 | 0 | 0 | 0 | 0 | 0 | 0.000877 | 0 | 0 | 0.032119 | 0 |
| k__*Bacteria;* p__*Firmicutes;* c__*Negativicutes;* o__*unidentified_Negativicutes;* f__*Selenomonadaceae* | 3.913657 | CRAB | 3.68011 | 0.540291 | 0 | 0 | 0 | 0 | 0 | 0.061126 | 0 | 0 | 0 | 0 | 0 |
| k__*Bacteria;* p__*Firmicutes;* c__*Negativicutes;* o__*unidentified_Negativicutes;* f__*Veillonellaceae* | 3.738469 |  |  |  | 0 | 0 | 0 | 0 | 0 | 0.0156 | 0 | 0.018629 | 0.011385 | 0 | 0 |
| k__*Bacteria;* p__*Gemmatimonadota;* c__*Gemmatimonadetes;* o__*Gemmatimonadales;* f__*Gemmatimonadaceae* | 4.467014 | CREC | 4.166851 | 0.603595 | 0 | 0.021653 | 0 | 0 | 0 | 0 | 0.003659 | 0 | 0 | 0.057393 | 0 |
| k__*Bacteria;* p__*Myxococcota;* c__*Myxococcia;* o__*Myxococcales;* f__*Anaeromyxobacteraceae* | 2.715168 | CRAB | 2.397387 | 0.540291 | 0 | 0 | 0 | 0 | 0 | 0 | 0.003175 | 0 | 0 | 0 | 0 |
| k__*Bacteria;* p__*Myxococcota;* c__*Myxococcia;* o__*Myxococcales;* f__*Myxococcaceae* | 4.6541 | CREC | 4.283851 | 0.363722 | 0 | 0.005857 | 0 | 0 | 0 | 0 | 0 | 0 | 0 | 0 | 0.055291 |
| k__*Bacteria;* p__*Myxococcota;* c__*Myxococcia;* o__*Myxococcales;* f__*Vulgatibacteraceae* | 2.755702 | CRAB | 2.545779 | 0.540291 | 0 | 0.002586 | 0 | 0 | 0 | 0 | 0 | 0 | 0 | 0 | 0 |
| k__*Bacteria;* p__*Myxococcota;* c__*unidentified_Myxococcota;* o__*Haliangiales;* f__*Haliangiaceae* | 3.219872 | CRAB | 2.949361 | 0.363722 | 0 | 0.00486 | 0 | 0 | 0 | 0 | 0.003599 | 0 | 0 | 0 | 0 |
| k__*Bacteria;* p__*Myxococcota;* c__*unidentified_Myxococcota;* o__*Polyangiales;* f__*Polyangiaceae* | 4.055203 | CRAB | 3.772348 | 0.363722 | 0 | 0.013833 | 0 | 0.038029 | 0 | 0 | 0 | 0 | 0 | 0 | 0 |
| k__*Bacteria;* p__*Nitrospirota;* c__*Nitrospiria;* o__*Nitrospirales;* f__*Nitrospiraceae* | 2.889702 | CRAB | 2.704106 | 0.540291 | 0 | 0.003521 | 0 | 0 | 0 | 0 | 0 | 0 | 0 | 0 | 0 |
| k__*Bacteria;* p__*Planctomycetes;* c__*unidentified_Planctomycetes;* o__*Gemmatales;* f__*Gemmataceae* | 3.546143 |  |  |  | 0 | 0.010281 | 0.003699 | 0 | 0 | 0 | 0 | 0 | 0 | 0.001748 | 0 |
| k__*Bacteria;* p__*Planctomycetes;* c__*unidentified_Planctomycetes;* o__*Isosphaerales;* f__*Isosphaeraceae* | 2.427689 | CRAB | 2.176329 | 0.540291 | 0 | 0.001215 | 0 | 0 | 0 | 0 | 0 | 0 | 0 | 0 | 0 |
| k__*Bacteria;* p__*Planctomycetota;* c__*Phycisphaerae;* o__*Tepidisphaerales;* f__*unidentified_Tepidisphaerales* | 2.179047 | CRAB | 1.991345 | 0.540291 | 0 | 0.000685 | 0 | 0 | 0 | 0 | 0 | 0 | 0 | 0 | 0 |
| k__*Bacteria;* p__*Proteobacteria;* c__*Alphaproteobacteria;* o__*Caulobacterales;* f__*Caulobacteraceae* | 4.242475 | CRAB | 3.915774 | 0.636328 | 0 | 0.009939 | 0 | 0.064279 | 0 | 0 | 0.00762 | 0 | 0 | 0 | 8.89E-05 |
| k__*Bacteria;* p__*Proteobacteria;* c__*Alphaproteobacteria;* o__*Caulobacterales;* f__*Hyphomonadaceae* | 3.423637 | CRAB | 3.132005 | 0.363722 | 0.007615 | 0.003614 | 0 | 0 | 0 | 0 | 0 | 0 | 0 | 0 | 0 |
| k__*Bacteria;* p__*Proteobacteria;* c__*Alphaproteobacteria;* o__*Micropepsales;* f__*Micropepsaceae* | 3.619387 | CRAB | 3.43411 | 0.363722 | 0 | 0.01564 | 0 | 0 | 0 | 0 | 0.004385 | 0 | 0 | 0 | 0 |
| k__*Bacteria;* p__*Proteobacteria;* c__*Alphaproteobacteria;* o__*Rhizobiales;* f__*Beijerinckiaceae* | 4.629892 | CRAB | 4.176214 | 0.656179 | 0 | 0.016076 | 0.047105 | 0 | 0.069333 | 0 | 0.004324 | 0 | 0 | 0.022667 | 0 |
| k__*Bacteria;* p__*Proteobacteria;* c__*Alphaproteobacteria;* o__*Rhizobiales;* f__*Devosiaceae* | 4.120898 | CREC | 3.777132 | 0.10247 | 0 | 0 | 0 | 0 | 0 | 0 | 0 | 0 | 0 | 0.025867 | 0 |
| k__*Bacteria;* p__*Proteobacteria;* c__*Alphaproteobacteria;* o__*Rhizobiales;* f__*Hyphomicrobiaceae* | 4.222853 | CREC | 3.922025 | 0.363722 | 0 | 0 | 0 | 0 | 0 | 0 | 0.007379 | 0 | 0 | 0.032711 | 0 |
| k__*Bacteria;* p__*Proteobacteria;* c__*Alphaproteobacteria;* o__*Rhizobiales;* f__*Rhizobiaceae* | 3.344927 | CRAB | 3.072452 | 0.363722 | 0 | 0.002617 | 0 | 0 | 0 | 0 | 0.010009 | 0 | 0 | 0 | 0 |
| k__*Bacteria;* p__*Proteobacteria;* c__*Alphaproteobacteria;* o__*Rhizobiales;* f__*Xanthobacteraceae* | 4.492959 | CRAB | 4.248306 | 0.095022 | 0 | 0.086083 | 0 | 0.01826 | 0 | 0.014651 | 0.025613 | 0.011047 | 0 | 0 | 0 |
| k__*Bacteria;* p__*Proteobacteria;* c__*Alphaproteobacteria;* o__*Rhodobacterales;* f__*Rhodobacteraceae* | 3.503219 | CRAB | 3.246252 | 0.237166 | 0 | 0 | 0 | 0 | 0 | 0.022541 | 0.000998 | 0 | 0.003795 | 0 | 0.000593 |
| k__*Bacteria;* p__*Proteobacteria;* c__*Alphaproteobacteria;* o__*Rhodospirillales;* f__*Rhodospirillaceae* | 3.548482 | CRAB | 3.275202 | 0.363722 | 0 | 0.003552 | 0 | 0 | 0 | 0 | 0.016844 | 0 | 0 | 0 | 0 |
| k__*Bacteria;* p__*Proteobacteria;* c__*Alphaproteobacteria;* o__*Rickettsiales;* f__*Rickettsiaceae* | 3.160681 | CRAB | 2.917014 | 0.540291 | 0 | 0 | 0 | 0 | 0 | 0.010796 | 0 | 0 | 0 | 0 | 0 |
| k__*Bacteria;* p__*Proteobacteria;* c__*Alphaproteobacteria;* o__*Rickettsiales;* f__*unidentified_Rickettsiales* | 2.600052 | CRAB | 2.382682 | 0.540291 | 0 | 0.001807 | 0 | 0 | 0 | 0 | 0 | 0 | 0 | 0 | 0 |
| k__*Bacteria;* p__*Proteobacteria;* c__*Alphaproteobacteria;* o__*Sphingomonadales;* f__*Sphingomonadaceae* | 4.297776 | CREC | 3.903609 | 0.823849 | 0 | 0.009129 | 0 | 0.047381 | 0 | 0.030311 | 0.000816 | 0 | 0.045333 | 0 | 0 |
| k__*Bacteria;* p__*Proteobacteria;* c__*Alphaproteobacteria;* o__*unidentified_Alphaproteobacteria;* f__*Acetobacteraceae* | 2.222829 | CRAB | 2.002342 | 0.540291 | 0 | 0 | 0 | 0 | 0 | 0.001246 | 0 | 0 | 0 | 0 | 0 |
| k__*Bacteria;* p__*Proteobacteria;* c__*Alphaproteobacteria;* o__*unidentified_Alphaproteobacteria;* f__*Azospirillaceae* | 3.126659 | CRAB | 2.938315 | 0.540291 | 0 | 0.006075 | 0 | 0 | 0 | 0 | 0 | 0 | 0 | 0 | 0 |
| k__*Bacteria;* p__*Proteobacteria;* c__*Alphaproteobacteria;* o__*unidentified_Alphaproteobacteria;* f__*Dongiaceae* | 2.108952 | CRAB | 1.775404 | 0.540291 | 0 | 0 | 0 | 0 | 0 | 0 | 0.000786 | 0 | 0 | 0 | 0 |
| k__*Bacteria;* p__*Proteobacteria;* c__*Alphaproteobacteria;* o__*unidentified_Alphaproteobacteria;* f__*Reyranellaceae* | 2.739714 | CRAB | 2.531179 | 0.540291 | 0 | 0.002492 | 0 | 0 | 0 | 0 | 0 | 0 | 0 | 0 | 0 |
| k__*Bacteria;* p__*Proteobacteria;* c__*Alphaproteobacteria;* o__*unidentified_Alphaproteobacteria;* f__*unidentified_Alphaproteobacteria* | 2.926529 | CRAB | 2.672981 | 0.540291 | 0 | 0.003832 | 0 | 0 | 0 | 0 | 0 | 0 | 0 | 0 | 0 |
| k__*Bacteria;* p__*Proteobacteria;* c__*Gammaproteobacteria;* o__*Burkholderiales;* f__*Alcaligenaceae* | 4.551546 | CREC | 4.24422 | 0.01543 | 0 | 0 | 0 | 0 | 0 | 0 | 0 | 0 | 0.037328 | 0.037719 | 0 |
| k__*Bacteria;* p__*Proteobacteria;* c__*Gammaproteobacteria;* o__*Burkholderiales;* f__*Burkholderiaceae* | 3.846924 | CRAB | 3.606727 | 0.540291 | 0 | 0.031903 | 0 | 0 | 0 | 0 | 0 | 0 | 0 | 0 | 0 |
| k__*Bacteria;* p__*Proteobacteria;* c__*Gammaproteobacteria;* o__*Burkholderiales;* f__*Comamonadaceae* | 4.622766 | CRAB | 4.173769 | 0.504266 | 0 | 0.055457 | 0 | 0 | 0.061073 | 0.03995 | 0.028093 | 0 | 0 | 0.025096 | 0 |
| k__*Bacteria;* p__*Proteobacteria;* c__*Gammaproteobacteria;* o__*Burkholderiales;* f__*Nitrosomonadaceae* | 3.955141 | CRAB | 3.691532 | 0.363722 | 0 | 0.039069 | 0 | 0 | 0 | 0 | 0.00251 | 0 | 0 | 0 | 0 |
| k__*Bacteria;* p__*Proteobacteria;* c__*Gammaproteobacteria;* o__*Burkholderiales;* f__*Oxalobacteraceae* | 4.885841 | CRAB | 4.493417 | 0.520703 | 0.151314 | 0.003178 | 0 | 0.146552 | 0 | 0.000178 | 0 | 0.039983 | 0.057549 | 0 | 0 |
| k__*Bacteria;* p__*Proteobacteria;* c__*Gammaproteobacteria;* o__*Burkholderiales;* f__*Rhodocyclaceae* | 2.597068 | CRAB | 2.301994 | 0.540291 | 0 | 0 | 0 | 0 | 0 | 0 | 0.002419 | 0 | 0 | 0 | 0 |
| k__*Bacteria;* p__*Proteobacteria;* c__*Gammaproteobacteria;* o__*Burkholderiales;* f__*Sutterellaceae* | 3.333944 | CRAB | 3.093781 | 0.363722 | 0 | 0.008132 | 0 | 0 | 0 | 0 | 0.002238 | 0 | 0 | 0 | 0 |
| k__*Bacteria;* p__*Proteobacteria;* c__*Gammaproteobacteria;* o__*Enterobacterales;* f__*Aeromonadaceae* | 3.57609 | CREC | 3.203264 | 0.026016 | 0 | 0 | 0 | 0 | 0 | 0.002373 | 0 | 0.001718 | 0.004536 | 0.0024 | 0.000682 |
| k__*Bacteria;* p__*Proteobacteria;* c__*Gammaproteobacteria;* o__*Enterobacterales;* f__*Enterobacteriaceae* | 4.568241 | CRAB | 4.246552 | 0.536564 | 0.00237 | 0.001807 | 0 | 0.001391 | 0 | 0.00083 | 0.212374 | 0.004946 | 0.007501 | 0 | 0.013215 |
| k__*Bacteria;* p__*Proteobacteria;* c__*Gammaproteobacteria;* o__*Pseudomonadales;* f__*Halieaceae* | 2.1711 | CRAB | 1.865068 | 0.540291 | 0 | 0 | 0 | 0 | 0 | 0 | 0.000907 | 0 | 0 | 0 | 0 |
| k__*Bacteria;* p__*Proteobacteria;* c__*Gammaproteobacteria;* o__*Pseudomonadales;* f__*Moraxellaceae* | 4.412103 | CREC | 3.859359 | 0.216446 | 0 | 0.007415 | 0 | 0.043445 | 0 | 0.024112 | 0.003357 | 0.002517 | 0.008035 | 0.028356 | 0.0096 |
| k__*Bacteria;* p__*Proteobacteria;* c__*Gammaproteobacteria;* o__*Pseudomonadales;* f__*Pseudomonadaceae* | 3.315604 |  |  |  | 0 | 0.003863 | 0 | 0 | 0 | 0.009076 | 0 | 0 | 0.002105 | 0 | 0 |
| k__*Bacteria;* p__*Proteobacteria;* c__*Gammaproteobacteria;* o__*Pseudomonadales;* f__*Spongiibacteraceae* | 2.401549 | CRAB | 2.134039 | 0.540291 | 0 | 0 | 0 | 0 | 0 | 0 | 0.001542 | 0 | 0 | 0 | 0 |
| k__*Bacteria;* p__*Proteobacteria;* c__*Gammaproteobacteria;* o__*Steroidobacterales;* f__*Steroidobacteraceae* | 2.977889 | CRAB | 2.740517 | 0.363722 | 0 | 0.003708 | 0 | 0 | 0 | 0 | 0.000816 | 0 | 0 | 0 | 0 |
| k__*Bacteria;* p__*Proteobacteria;* c__*Gammaproteobacteria;* o__*unidentified_Gammaproteobacteria;* f__*unidentified_Gammaproteobacteria* | 4.508995 | CREC | 4.123196 | 0.363722 | 0 | 0.02103 | 0 | 0 | 0 | 0 | 0 | 0 | 0 | 0 | 0.039586 |
| k__*Bacteria;* p__*Proteobacteria;* c__*Gammaproteobacteria;* o__*Xanthomonadales;* f__*Rhodanobacteraceae* | 3.709363 | CRAB | 3.507902 | 0.540291 | 0 | 0.023242 | 0 | 0 | 0 | 0 | 0 | 0 | 0 | 0 | 0 |
| k__*Bacteria;* p__*Proteobacteria;* c__*Gammaproteobacteria;* o__*Xanthomonadales;* f__*Xanthomonadaceae* | 2.765449 | CRAB | 2.475199 | 0.363722 | 0 | 0.000312 | 0 | 0 | 0 | 0 | 0.003145 | 0 | 0 | 0 | 0 |
| k__*Bacteria;* p__*Spirochaetota;* c__*Spirochaetia;* o__*Spirochaetales;* f__*Spirochaetaceae* | 3.525274 | CRAB | 3.279299 | 0.540291 | 0 | 0 | 0 | 0 | 0 | 0 | 0 | 0.018452 | 0 | 0 | 0 |
| k__*Bacteria;* p__*Thermotogota;* c__*Thermotogae;* o__*Petrotogales;* f__*Petrotogaceae* | 3.26354 | CRAB | 3.056225 | 0.15332 | 0 | 0 | 0 | 0 | 0 | 0 | 0 | 0.0101 | 0.002283 | 0 | 5.93E-05 |
| k__*Bacteria;* p__*unidentified_Bacteria;* c__*Anaerolineae;* o__*Ardenticatenales;* f__*Ardenticatenaceae* | 2.656484 | CRAB | 2.434518 | 0.540291 | 0 | 0 | 0 | 0 | 0 | 0.003381 | 0 | 0 | 0 | 0 | 0 |
| k__*Bacteria;* p__*unidentified_Bacteria;* c__*Ktedonobacteria;* o__*Ktedonobacterales;* f__*Ktedonobacteraceae* | 3.411812 | CRAB | 3.224352 | 0.540291 | 0 | 0.011714 | 0 | 0 | 0 | 0 | 0 | 0 | 0 | 0 | 0 |
| k__*Bacteria;* p__*Verrucomicrobiota;* c__*Verrucomicrobiae;* o__*Chthoniobacterales;* f__*Chthoniobacteraceae* | 3.634418 |  |  |  | 0 | 0.013397 | 0 | 0.004676 | 0 | 0 | 0.002056 | 0 | 0 | 0.005659 | 0 |
| k__*Bacteria;* p__*Verrucomicrobiota;* c__*Verrucomicrobiae;* o__*Methylacidiphilales;* f__*Methylacidiphilaceae* | 2.299022 | CRAB | 2.094783 | 0.540291 | 0 | 0.000904 | 0 | 0 | 0 | 0 | 0 | 0 | 0 | 0 | 0 |
| k__*Bacteria;* p__*Verrucomicrobiota;* c__*Verrucomicrobiae;* o__*unidentified_Verrucomicrobiae;* f__*unidentified_Verrucomicrobiae* | 2.656168 | CRAB | 2.448283 | 0.540291 | 0 | 0.002056 | 0 | 0 | 0 | 0 | 0 | 0 | 0 | 0 | 0 |
| k__*Bacteria;* p__*Acidobacteriota;* c__*Acidobacteriae;* o__*Acidobacteriales;* f__*unidentified_Acidobacteriales;* g__*Occallatibacter* | 3.712013 | CRAB | 3.386354 | 0.540291 | 0 | 0.010531 | 0 | 0 | 0 | 0 | 0 | 0 | 0 | 0 | 0 |
| k__*Bacteria;* p__*Acidobacteriota;* c__*Acidobacteriae;* o__*Bryobacterales;* f__*Bryobacteraceae;* g__*Bryobacter* | 4.332661 | CRAB | 3.97854 | 0.363722 | 0 | 0.016357 | 0 | 0 | 0 | 0 | 0.071971 | 0 | 0 | 0 | 0 |
| k__*Bacteria;* p__*Acidobacteriota;* c__*unidentified_Acidobacteriota;* o__*unidentified_Acidobacteriota;* f__*unidentified_Acidobacteriota;* g__*unidentified_Acidobacteriota* | 4.105983 | CRAB | 3.762104 | 0.363722 | 0 | 0.020937 | 0 | 0 | 0 | 0 | 0.013426 | 0 | 0 | 0 | 0 |
| k__*Bacteria;* p__*Acidobacteriota;* c__*Vicinamibacteria;* o__*Vicinamibacterales;* f__*unidentified_Vicinamibacterales;* g__*unidentified_Vicinamibacterales* | 3.158746 | CRAB | 2.955325 | 0.540291 | 0 | 0 | 0 | 0.004913 | 0 | 0 | 0 | 0 | 0 | 0 | 0 |
| k__*Bacteria;* p__*Actinobacteria;* c__*unidentified_Actinobacteria;* o__*Actinomycetales;* f__*Actinomycetaceae;* g__*Mobiluncus* | 2.076233 | CRAB | 1.701883 | 0.540291 | 0 | 0 | 0 | 0 | 0 | 0 | 0.000635 | 0 | 0 | 0 | 0 |
| k__*Bacteria;* p__*Actinobacteria;* c__*unidentified_Actinobacteria;* o__*Catenulisporales;* f__*Catenulisporaceae;* g__*Catenulispora* | 2.525519 | CRAB | 2.192618 | 0.540291 | 0 | 0.000685 | 0 | 0 | 0 | 0 | 0 | 0 | 0 | 0 | 0 |
| k__*Bacteria;* p__*Actinobacteria;* c__*unidentified_Actinobacteria;* o__*Corynebacteriales;* f__*Corynebacteriaceae;* g__*Corynebacterium* | 3.999559 | CRAB | 3.714738 | 0.504266 | 0.017333 | 0 | 0 | 0.00802 | 0 | 0.003945 | 0.003659 | 0 | 0.003083 | 0 | 0 |
| k__*Bacteria;* p__*Actinobacteria;* c__*unidentified_Actinobacteria;* o__*Corynebacteriales;* f__*Mycobacteriaceae;* g__*Mycobacterium* | 3.449183 | CRAB | 3.204925 | 0.540291 | 0 | 0 | 0 | 0.009589 | 0 | 0 | 0 | 0 | 0 | 0 | 0 |
| k__*Bacteria;* p__*Actinobacteria;* c__*unidentified_Actinobacteria;* o__*Frankiales;* f__*Acidothermaceae;* g__*Acidothermus* | 2.581036 | CRAB | 2.226066 | 0.540291 | 0 | 0.000779 | 0 | 0 | 0 | 0 | 0 | 0 | 0 | 0 | 0 |
| k__*Bacteria;* p__*Actinobacteria;* c__*unidentified_Actinobacteria;* o__*Frankiales;* f__*Geodermatophilaceae;* g__*Blastococcus* | 2.251348 | CRAB | 1.884607 | 0.363722 | 0 | 0.000156 | 0 | 0 | 0 | 0 | 0.000544 | 0 | 0 | 0 | 0 |
| k__*Bacteria;* p__*Actinobacteria;* c__*unidentified_Actinobacteria;* o__*Frankiales;* f__*Geodermatophilaceae;* g__*Modestobacter* | 2.654641 | CRAB | 2.426092 | 0.540291 | 0 | 0 | 0 | 0.001539 | 0 | 0 | 0 | 0 | 0 | 0 | 0 |
| k__*Bacteria;* p__*Actinobacteria;* c__*unidentified_Actinobacteria;* o__*Micrococcales;* f__*Cellulomonadaceae;* g__*Cellulomonas* | 3.805921 | CREC | 3.481622 | 0.10247 | 0 | 0 | 0 | 0 | 0 | 0 | 0 | 0 | 0 | 0.00803 | 0 |
| k__*Bacteria;* p__*Actinobacteria;* c__*unidentified_Actinobacteria;* o__*Micrococcales;* f__*Intrasporangiaceae;* g__*Ornithinibacter* | 2.651641 | CRAB | 2.267893 | 0.540291 | 0 | 0 | 0 | 0 | 0 | 0 | 0.002389 | 0 | 0 | 0 | 0 |
| k__*Bacteria;* p__*Actinobacteria;* c__*unidentified_Actinobacteria;* o__*Micrococcales;* f__*Microbacteriaceae;* g__*Agromyces* | 3.820579 | CREC | 3.443982 | 0.01543 | 0 | 0 | 0 | 0 | 0 | 0 | 0 | 0 | 0.007768 | 0.001867 | 0 |
| k__*Bacteria;* p__*Actinobacteria;* c__*unidentified_Actinobacteria;* o__*Micrococcales;* f__*Microbacteriaceae;* g__*Leucobacter* | 3.018107 | CRAB | 2.867735 | 0.540291 | 0 | 0 | 0 | 0 | 0 | 0.004538 | 0 | 0 | 0 | 0 | 0 |
| k__*Bacteria;* p__*Actinobacteria;* c__*unidentified_Actinobacteria;* o__*Micrococcales;* f__*Microbacteriaceae;* g__*Microbacterium* | 3.140912 | CRAB | 2.999176 | 0.540291 | 0 | 0 | 0 | 0 | 0 | 0.006021 | 0 | 0 | 0 | 0 | 0 |
| k__*Bacteria;* p__*Actinobacteria;* c__*unidentified_Actinobacteria;* o__*Micrococcales;* f__*Micrococcaceae;* g__*Arthrobacter* | 4.420616 | CREC | 4.067277 | 0.363722 | 0.025807 | 0 | 0 | 0 | 0 | 0 | 0 | 0 | 0 | 0.033067 | 0 |
| k__*Bacteria;* p__*Actinobacteria;* c__*unidentified_Actinobacteria;* o__*Micrococcales;* f__*Micrococcaceae;* g__*Micrococcus* | 4.258574 |  |  |  | 0.04711 | 0 | 0 | 0 | 0 | 0.007444 | 0 | 0 | 0 | 0.003141 | 0 |
| k__*Bacteria;* p__*Actinobacteria;* c__*unidentified_Actinobacteria;* o__*Micrococcales;* f__*Micrococcaceae;* g__*Pseudarthrobacter* | 3.921395 | CRAB | 3.517707 | 0.540291 | 0 | 0 | 0 | 0 | 0.017851 | 0 | 0 | 0 | 0 | 0 | 0 |
| k__*Bacteria;* p__*Actinobacteria;* c__*unidentified_Actinobacteria;* o__*Propionibacteriales;* f__*Nocardioidaceae;* g__*Nocardioides* | 3.324508 | CRAB | 3.026736 | 0.91138 | 0.003052 | 0.001838 | 0 | 0 | 0 | 0 | 0.000786 | 0 | 0 | 2.96E-05 | 0.000267 |
| k__*Bacteria;* p__*Actinobacteria;* c__*unidentified_Actinobacteria;* o__*Propionibacteriales;* f__*Propionibacteriaceae;* g__*Cutibacterium* | 4.797885 | CREC | 4.462424 | 0.094638 | 5.93E-05 | 0.003053 | 0 | 0 | 0 | 0.005457 | 6.05E-05 | 0 | 0.002698 | 0.076415 | 0.000119 |
| k__*Bacteria;* p__*Actinobacteria;* c__*unidentified_Actinobacteria;* o__*Propionibacteriales;* f__*Propionibacteriaceae;* g__*Microlunatus* | 2.727164 | CRAB | 2.384394 | 0.540291 | 0 | 0.00109 | 0 | 0 | 0 | 0 | 0 | 0 | 0 | 0 | 0 |
| k__*Bacteria;* p__*Actinobacteria;* c__*unidentified_Actinobacteria;* o__*Streptomycetales;* f__*Streptomycetaceae;* g__*Streptomyces* | 3.034355 | CRAB | 2.690319 | 0.540291 | 0 | 0.002212 | 0 | 0 | 0 | 0 | 0 | 0 | 0 | 0 | 0 |
| k__*Bacteria;* p__*Actinobacteria;* c__*unidentified_Actinobacteria;* o__*Streptosporangiales;* f__*Nocardiopsaceae;* g__*Nocardiopsis* | 2.754113 | CREC | 2.363672 | 0.10247 | 0 | 0 | 0 | 0 | 0 | 0 | 0 | 0 | 0.00086 | 0 | 0 |
| k__*Bacteria;* p__*Actinobacteriota;* c__*Acidimicrobiia;* o__*unidentified_Acidimicrobiia;* f__*Iamiaceae;* g__*Iamia* | 2.816565 | CRAB | 2.479663 | 0.540291 | 0 | 0.00134 | 0 | 0 | 0 | 0 | 0 | 0 | 0 | 0 | 0 |
| k__*Bacteria;* p__*Actinobacteriota;* c__*Acidimicrobiia;* o__*unidentified_Acidimicrobiia;* f__*unidentified_Acidimicrobiia;* g__*unidentified_Acidimicrobiia* | 3.649924 | CRAB | 3.38303 | 0.363722 | 0 | 0.004798 | 0 | 0.007221 | 0 | 0 | 0 | 0 | 0 | 0 | 0 |
| k__*Bacteria;* p__*Actinobacteriota;* c__*Coriobacteriia;* o__*Coriobacteriales;* f__*Atopobiaceae;* g__*Olsenella* | 3.374995 | CRAB | 3.285797 | 0.540291 | 0 | 0 | 0 | 0 | 0 | 0.010321 | 0 | 0 | 0 | 0 | 0 |
| k__*Bacteria;* p__*Actinobacteriota;* c__*Coriobacteriia;* o__*Coriobacteriales;* f__*Eggerthellaceae;* g__*Eggerthella* | 3.264843 | CREC | 2.894878 | 0.10247 | 0 | 0 | 0 | 0 | 0 | 0 | 0 | 0 | 0.002787 | 0 | 0 |
| k__*Bacteria;* p__*Actinobacteriota;* c__*Coriobacteriia;* o__*Coriobacteriales;* f__*Eggerthellaceae;* g__*Slackia* | 3.11217 | CRAB | 2.980554 | 0.540291 | 0 | 0 | 0 | 0 | 0 | 0.005635 | 0 | 0 | 0 | 0 | 0 |
| k__*Bacteria;* p__*Actinobacteriota;* c__*Thermoleophilia;* o__*Gaiellales;* f__*Gaiellaceae;* g__*Gaiella* | 3.764045 | CRAB | 3.518061 | 0.242692 | 0 | 0.000592 | 0 | 0.01823 | 0 | 0 | 0.000907 | 0 | 0 | 0 | 0 |
| k__*Bacteria;* p__*Actinobacteriota;* c__*Thermoleophilia;* o__*Solirubrobacterales;* f__*Solirubrobacteraceae;* g__*Conexibacter* | 3.300367 | CRAB | 2.917138 | 0.540291 | 0 | 0.004081 | 0 | 0 | 0 | 0 | 0 | 0 | 0 | 0 | 0 |
| k__*Bacteria;* p__*Actinobacteriota;* c__*Thermoleophilia;* o__*Solirubrobacterales;* f__*Solirubrobacteraceae;* g__*Solirubrobacter* | 3.835019 | CRAB | 3.621561 | 0.540291 | 0.019614 | 0 | 0 | 0 | 0 | 0 | 0 | 0 | 0 | 0 | 0 |
| k__*Bacteria;* p__*Bacteroidota;* c__*Bacteroidia;* o__*Bacteroidales;* f__*Bacteroidaceae;* g__*Bacteroides* | 4.136199 | CRAB | 3.777269 | 0.242692 | 0 | 0 | 0.00287 | 0 | 0 | 0.009461 | 0 | 0.034919 | 0 | 0 | 0 |
| k__*Bacteria;* p__*Bacteroidota;* c__*Bacteroidia;* o__*Bacteroidales;* f__*Marinifilaceae;* g__*Odoribacter* | 3.445835 | CRAB | 3.036362 | 0.540291 | 0 | 0 | 0 | 0 | 0 | 0 | 0 | 0.010188 | 0 | 0 | 0 |
| k__*Bacteria;* p__*Bacteroidota;* c__*Bacteroidia;* o__*Bacteroidales;* f__*Porphyromonadaceae;* g__*Porphyromonas* | 3.879426 | CREC | 3.465126 | 0.10247 | 0 | 0 | 0 | 0 | 0 | 0 | 0 | 0 | 0.011474 | 0 | 0 |
| k__*Bacteria;* p__*Bacteroidota;* c__*Bacteroidia;* o__*Bacteroidales;* f__*Prevotellaceae;* g__*Prevotella* | 3.602602 | CRAB | 3.317864 | 0.363722 | 0 | 0 | 0 | 0.009115 | 0 | 0 | 0 | 0.004857 | 0 | 0 | 0 |
| k__*Bacteria;* p__*Bacteroidota;* c__*Bacteroidia;* o__*Bacteroidales;* f__*Prevotellaceae;* g__*unidentified_Prevotellaceae* | 3.380684 | CRAB | 3.217616 | 0.363722 | 0 | 0 | 0 | 0 | 0 | 0.008868 | 0 | 0.001333 | 0 | 0 | 0 |
| k__*Bacteria;* p__*Bacteroidota;* c__*Bacteroidia;* o__*Bacteroidales;* f__*Rikenellaceae;* g__*Alistipes* | 4.160264 | CREC | 3.825484 | 0.15332 | 0 | 0 | 0 | 0 | 0 | 0 | 0 | 0.044663 | 0.011207 | 0 | 0.006074 |
| k__*Bacteria;* p__*Bacteroidota;* c__*Bacteroidia;* o__*Bacteroidales;* f__*Rikenellaceae;* g__*unidentified_Rikenellaceae* | 3.514979 | CRAB | 3.260599 | 0.540291 | 0 | 0 | 0 | 0.011157 | 0 | 0 | 0 | 0 | 0 | 0 | 0 |
| k__*Bacteria;* p__*Bacteroidota;* c__*Bacteroidia;* o__*Bacteroidales;* f__*Tannerellaceae;* g__*Parabacteroides* | 3.492475 | CRAB | 3.109047 | 0.540291 | 0 | 0 | 0 | 0 | 0 | 0 | 0 | 0.011343 | 0 | 0 | 0 |
| k__*Bacteria;* p__*Bacteroidota;* c__*Bacteroidia;* o__*Chitinophagales;* f__*Chitinophagaceae;* g__*Ferruginibacter* | 3.260519 | CRAB | 2.900499 | 0.540291 | 0 | 0 | 0 | 0 | 0 | 0 | 0.009707 | 0 | 0 | 0 | 0 |
| k__*Bacteria;* p__*Bacteroidota;* c__*Bacteroidia;* o__*Chitinophagales;* f__*Chitinophagaceae;* g__*Puia* | 3.350413 | CRAB | 2.958153 | 0.540291 | 0 | 0.00458 | 0 | 0 | 0 | 0 | 0 | 0 | 0 | 0 | 0 |
| k__*Bacteria;* p__*Bacteroidota;* c__*Bacteroidia;* o__*Cytophagales;* f__*Hymenobacteraceae;* g__*Adhaeribacter* | 2.660217 | CRAB | 2.324612 | 0.540291 | 0 | 0.000935 | 0 | 0 | 0 | 0 | 0 | 0 | 0 | 0 | 0 |
| k__*Bacteria;* p__*Bacteroidota;* c__*Bacteroidia;* o__*Cytophagales;* f__*Hymenobacteraceae;* g__*Nibribacter* | 4.379418 | CREC | 4.056557 | 0.10247 | 0 | 0 | 0 | 0 | 0 | 0 | 0 | 0 | 0 | 0.030074 | 0 |
| k__*Bacteria;* p__*Bacteroidota;* c__*Bacteroidia;* o__*Flavobacteriales;* f__*Flavobacteriaceae;* g__*Flavobacterium* | 3.481515 | CRAB | 3.082201 | 0.242692 | 0 | 0.000592 | 0 | 0 | 0 | 0 | 0.001935 | 0.008678 | 0 | 0 | 0 |
| k__*Bacteria;* p__*Bacteroidota;* c__*Bacteroidia;* o__*Flavobacteriales;* f__*Flavobacteriaceae;* g__*Myroides* | 3.102929 | CRAB | 2.971123 | 0.540291 | 0 | 0 | 0 | 0 | 0 | 0.005517 | 0 | 0 | 0 | 0 | 0 |
| k__*Bacteria;* p__*Bacteroidota;* c__*Bacteroidia;* o__*Flavobacteriales;* f__*Weeksellaceae;* g__*Ornithobacterium* | 4.0201 | CRAB | 3.81546 | 0.363722 | 0 | 0 | 0 | 0 | 0 | 0.033366 | 0 | 0.010248 | 0 | 0 | 0 |
| k__*Bacteria;* p__*Bacteroidota;* c__*Bacteroidia;* o__*Sphingobacteriales;* f__*Sphingobacteriaceae;* g__*Mucilaginibacter* | 3.359187 | CRAB | 3.002655 | 0.540291 | 0 | 0.004673 | 0 | 0 | 0 | 0 | 0 | 0 | 0 | 0 | 0 |
| k__*Bacteria;* p__*Cyanobacteria;* c__*Sericytochromatia;* o__*unidentified_Sericytochromatia;* f__*unidentified_Sericytochromatia;* g__*unidentified_Sericytochromatia* | 2.525519 | CRAB | 2.184542 | 0.540291 | 0 | 0.000685 | 0 | 0 | 0 | 0 | 0 | 0 | 0 | 0 | 0 |
| k__*Bacteria;* p__*Cyanobacteria;* c__*unidentified_Cyanobacteria;* o__*Leptolyngbyales;* f__*Leptolyngbyaceae;* g__*unidentified_Leptolyngbyaceae* | 3.778853 | CREC | 3.401561 | 0.10247 | 0 | 0 | 0 | 0 | 0 | 0 | 0 | 0 | 0.009102 | 0 | 0 |
| k__*Bacteria;* p__*Cyanobacteria;* c__*unidentified_Cyanobacteria;* o__*unidentified_Cyanobacteria;* f__*Nostocaceae;* g__*unidentified_Nostocaceae* | 4.114537 | CREC | 3.708295 | 0.10247 | 0 | 0 | 0 | 0 | 0 | 0 | 0 | 0 | 0.019717 | 0 | 0 |
| k__*Bacteria;* p__*Deinococcota;* c__*Deinococci;* o__*Deinococcales;* f__*Deinococcaceae;* g__*Deinococcus* | 4.446139 | CREC | 4.015689 | 0.363722 | 0 | 0 | 0 | 0 | 0 | 0.019248 | 0 | 0 | 0.042309 | 0 | 0 |
| k__*Bacteria;* p__*Desulfobacterota;* c__*Syntrophia;* o__*Syntrophales;* f__*Smithellaceae;* g__*Smithella* | 4.155599 | CRAB | 3.741087 | 0.540291 | 0 | 0 | 0 | 0 | 0.030611 | 0 | 0 | 0 | 0 | 0 | 0 |
| k__*Bacteria;* p__*Desulfobacterota;* c__*Syntrophorhabdia;* o__*Syntrophorhabdales;* f__*Syntrophorhabdaceae;* g__*Syntrophorhabdus* | 3.732751 | CRAB | 3.335151 | 0.540291 | 0 | 0 | 0 | 0 | 0 | 0 | 0 | 0.019725 | 0 | 0 | 0 |
| k__*Bacteria;* p__*Firmicutes;* c__*Bacilli;* o__*Bacillales;* f__*Bacillaceae;* g__*Bacillus* | 4.909659 | CREC | 4.522511 | 0.603595 | 0 | 0 | 0 | 0 | 0 | 0.088798 | 0 | 0.072385 | 0.123014 | 0 | 0 |
| k__*Bacteria;* p__*Firmicutes;* c__*Bacilli;* o__*Bacillales;* f__*Bacillaceae;* g__*Sinibacillus* | 2.708617 | CRAB | 2.323852 | 0.540291 | 0 | 0 | 0 | 0 | 0 | 0 | 0 | 0.001866 | 0 | 0 | 0 |
| k__*Bacteria;* p__*Firmicutes;* c__*Bacilli;* o__*Bacillales;* f__*Planococcaceae;* g__*Sporosarcina* | 4.200737 | CRAB | 3.856394 | 0.363722 | 0 | 0.001464 | 0.022398 | 0 | 0 | 0 | 0 | 0 | 0 | 0 | 0 |
| k__*Bacteria;* p__*Firmicutes;* c__*Bacilli;* o__*Erysipelotrichales;* f__*Erysipelotrichaceae;* g__*Allobaculum* | 2.999285 | CREC | 2.583395 | 0.10247 | 0 | 0 | 0 | 0 | 0 | 0 | 0 | 0 | 0.001512 | 0 | 0 |
| k__*Bacteria;* p__*Firmicutes;* c__*Bacilli;* o__*Erysipelotrichales;* f__*Erysipelotrichaceae;* g__*Dubosiella* | 4.027955 | CRAB | 3.793295 | 0.363722 | 0 | 0 | 0 | 0 | 0 | 0.025121 | 0 | 0.017859 | 0 | 0 | 0 |
| k__*Bacteria;* p__*Firmicutes;* c__*Bacilli;* o__*Erysipelotrichales;* f__*Erysipelotrichaceae;* g__*Turicibacter* | 3.612568 | CRAB | 3.23821 | 0.540291 | 0 | 0 | 0 | 0 | 0 | 0 | 0 | 0.014957 | 0 | 0 | 0 |
| k__*Bacteria;* p__*Firmicutes;* c__*Bacilli;* o__*Lactobacillales;* f__*Aerococcaceae;* g__*Facklamia* | 4.544594 | CREC | 4.310779 | 0.10247 | 0 | 0 | 0 | 0 | 0 | 0 | 0 | 0 | 0 | 0 | 0.030134 |
| k__*Bacteria;* p__*Firmicutes;* c__*Bacilli;* o__*Lactobacillales;* f__*Enterococcaceae;* g__*Enterococcus* | 4.810344 | CRAB | 4.434919 | 0.636328 | 0 | 0 | 0 | 0 | 0 | 0.031794 | 0.273882 | 0.021561 | 0.008746 | 0 | 0 |
| k__*Bacteria;* p__*Firmicutes;* c__*Bacilli;* o__*Lactobacillales;* f__*Enterococcaceae;* g__*Melissococcus* | 3.994146 | CREC | 3.611336 | 0.10247 | 0 | 0 | 0 | 0 | 0 | 0 | 0 | 0 | 0.014943 | 0 | 0 |
| k__*Bacteria;* p__*Firmicutes;* c__*Bacilli;* o__*Lactobacillales;* f__*Lactobacillaceae;* g__*Lacticaseibacillus* | 3.17386 | CRAB | 3.047118 | 0.540291 | 0 | 0 | 0 | 0 | 0 | 0.006495 | 0 | 0 | 0 | 0 | 0 |
| k__*Bacteria;* p__*Firmicutes;* c__*Bacilli;* o__*Lactobacillales;* f__*Lactobacillaceae;* g__*Lactobacillus* | 4.421046 |  |  |  | 0 | 0 | 0.02154 | 0.026043 | 0 | 0.017943 | 0 | 8.89E-05 | 0.006137 | 0 | 0.000563 |
| k__*Bacteria;* p__*Firmicutes;* c__*Bacilli;* o__*Lactobacillales;* f__*Lactobacillaceae;* g__*Ligilactobacillus* | 4.901874 | CREC | 4.573585 | 0.316494 | 0 | 0 | 0 | 0.000474 | 0 | 0.017943 | 0 | 0.022095 | 0.009725 | 0.092089 | 0 |
| k__*Bacteria;* p__*Firmicutes;* c__*Bacilli;* o__*Lactobacillales;* f__*Lactobacillaceae;* g__*Weissella* | 3.531924 | CRAB | 3.27947 | 0.540291 | 0 | 0 | 0 | 0.011601 | 0 | 0 | 0 | 0 | 0 | 0 | 0 |
| k__*Bacteria;* p__*Firmicutes;* c__*Bacilli;* o__*Lactobacillales;* f__*Streptococcaceae;* g__*Streptococcus* | 4.576371 | CREC | 4.18145 | 0.603595 | 0 | 0 | 0 | 0 | 0 | 0.011982 | 0 | 0.013387 | 0.057104 | 0 | 0 |
| k__*Bacteria;* p__*Firmicutes;* c__*Bacilli;* o__*unidentified_Bacilli;* f__*Staphylococcaceae;* g__*Staphylococcus* | 5.215368 | CRAB | 4.854376 | 0.156039 | 0.043376 | 0 | 0.104773 | 0 | 0.156281 | 0.022244 | 0 | 0 | 0 | 0 | 0 |
| k__*Bacteria;* p__*Firmicutes;* c__*Bacilli;* o__*unidentified_Bacilli;* f__*unidentified_Bacilli;* g__*Aneurinibacillus* | 3.300212 | CRAB | 2.863668 | 0.540291 | 0 | 0 | 0 | 0 | 0 | 0 | 0 | 0.007286 | 0 | 0 | 0 |
| k__*Bacteria;* p__*Firmicutes;* c__*Clostridia;* o__*Clostridiales;* f__*Clostridiaceae;* g__*unidentified_Clostridiaceae* | 3.534984 | CRAB | 3.422204 | 0.540291 | 0 | 0 | 0 | 0 | 0 | 0.014918 | 0 | 0 | 0 | 0 | 0 |
| k__*Bacteria;* p__*Firmicutes;* c__*Clostridia;* o__*Lachnospirales;* f__*Defluviitaleaceae;* g__*Defluviitalea* | 4.350416 | CRAB | 4.000136 | 0.540291 | 0 | 0 | 0.033109 | 0 | 0 | 0 | 0 | 0 | 0 | 0 | 0 |
| k__*Bacteria;* p__*Firmicutes;* c__*Clostridia;* o__*Lachnospirales;* f__*Lachnospiraceae;* g__*Anaerostipes* | 2.155635 | CRAB | 2.032424 | 0.540291 | 0 | 0 | 0 | 0 | 0 | 0.000623 | 0 | 0 | 0 | 0 | 0 |
| k__*Bacteria;* p__*Firmicutes;* c__*Clostridia;* o__*Lachnospirales;* f__*Lachnospiraceae;* g__*Blautia* | 4.17367 | CREC | 3.802551 | 0.603595 | 0 | 0 | 0 | 0 | 0 | 0.002373 | 0.00626 | 0 | 0.022593 | 0 | 0 |
| k__*Bacteria;* p__*Firmicutes;* c__*Clostridia;* o__*Lachnospirales;* f__*Lachnospiraceae;* g__*Coprococcus* | 3.499882 | CRAB | 3.264644 | 0.540291 | 0.009066 | 0 | 0 | 0 | 0 | 0 | 0 | 0 | 0 | 0 | 0 |
| k__*Bacteria;* p__*Firmicutes;* c__*Clostridia;* o__*Lachnospirales;* f__*Lachnospiraceae;* g__*Lachnoclostridium* | 3.723278 | CRAB | 3.591295 | 0.540291 | 0 | 0 | 0 | 0 | 0 | 0.023015 | 0 | 0 | 0 | 0 | 0 |
| k__*Bacteria;* p__*Firmicutes;* c__*Clostridia;* o__*Lachnospirales;* f__*Lachnospiraceae;* g__*Roseburia* | 4.044355 | CRAB | 3.759001 | 0.544827 | 0.031762 | 0 | 0 | 0 | 0 | 0 | 0 | 0 | 0 | 0 | 0.006845 |
| k__*Bacteria;* p__*Firmicutes;* c__*Clostridia;* o__*Lachnospirales;* f__*Lachnospiraceae;* g__*unidentified_Lachnospiraceae* | 3.514441 | CRAB | 3.265285 | 0.363722 | 0 | 0 | 0 | 0 | 0 | 0.005576 | 0 | 0.007256 | 0 | 0 | 0 |
| k__*Bacteria;* p__*Firmicutes;* c__*Clostridia;* o__*unidentified_Clostridia;* f__*Peptostreptococcaceae;* g__*Filifactor* | 3.796731 | CRAB | 3.667125 | 0.540291 | 0 | 0 | 0 | 0 | 0 | 0.027256 | 0 | 0 | 0 | 0 | 0 |
| k__*Bacteria;* p__*Firmicutes;* c__*Clostridia;* o__*unidentified_Clostridia;* f__*Peptostreptococcaceae;* g__*Peptoclostridium* | 5.210869 | CREC | 4.958787 | 0.051518 | 0 | 0 | 0 | 0 | 0.001599 | 0 | 0 | 0 | 0.00424 | 0 | 0.137337 |
| k__*Bacteria;* p__*Firmicutes;* c__*Clostridia;* o__*unidentified_Clostridia;* f__*Peptostreptococcaceae;* g__*Romboutsia* | 4.071312 | CREC | 3.624623 | 0.10247 | 0 | 0 | 0 | 0 | 0 | 0 | 0 | 0 | 0.017849 | 0 | 0 |
| k__*Bacteria;* p__*Firmicutes;* c__*Clostridia;* o__*unidentified_Clostridia;* f__*Ruminococcaceae;* g__*Paludicola* | 4.009302 | CRAB | 3.62179 | 0.540291 | 0 | 0 | 0 | 0 | 0 | 0 | 0 | 0.037288 | 0 | 0 | 0 |
| k__*Bacteria;* p__*Firmicutes;* c__*Clostridia;* o__*unidentified_Clostridia;* f__*unidentified_Clostridia;* g__*Anaerococcus* | 4.407981 | CREC | 4.105614 | 0.10247 | 0 | 0 | 0 | 0 | 0 | 0 | 0 | 0 | 0 | 0.032119 | 0 |
| k__*Bacteria;* p__*Firmicutes;* c__*Clostridia;* o__*unidentified_Clostridia;* f__*unidentified_Clostridia;* g__*Finegoldia* | 2.216412 | CRAB | 1.864728 | 0.540291 | 0 | 0 | 0 | 0 | 0 | 0 | 0.000877 | 0 | 0 | 0 | 0 |
| k__*Bacteria;* p__*Firmicutes;* c__*Negativicutes;* o__*unidentified_Negativicutes;* f__*Selenomonadaceae;* g__*Selenomonas* | 3.85708 | CRAB | 3.711628 | 0.540291 | 0 | 0 | 0 | 0 | 0 | 0.03132 | 0 | 0 | 0 | 0 | 0 |
| k__*Bacteria;* p__*Firmicutes;* c__*Negativicutes;* o__*unidentified_Negativicutes;* f__*Veillonellaceae;* g__*Dialister* | 3.707927 | CRAB | 3.358723 | 0.540291 | 0 | 0 | 0 | 0 | 0 | 0 | 0 | 0.018629 | 0 | 0 | 0 |
| k__*Bacteria;* p__*Firmicutes;* c__*Negativicutes;* o__*unidentified_Negativicutes;* f__*Veillonellaceae;* g__*Megasphaera* | 3.876046 | CREC | 3.55706 | 0.544827 | 0 | 0 | 0 | 0 | 0 | 0.0156 | 0 | 0 | 0.011385 | 0 | 0 |
| k__*Bacteria;* p__*Gemmatimonadota;* c__*Gemmatimonadetes;* o__*Gemmatimonadales;* f__*Gemmatimonadaceae;* g__*Gemmatimonas* | 4.087282 | CREC | 3.789578 | 0.363722 | 0 | 0.003147 | 0 | 0 | 0 | 0 | 0 | 0 | 0 | 0.015348 | 0 |
| k__*Bacteria;* p__*Gemmatimonadota;* c__*Gemmatimonadetes;* o__*Gemmatimonadales;* f__*Gemmatimonadaceae;* g__*unidentified_Gemmatimonadaceae* | 3.350413 | CRAB | 3.013347 | 0.540291 | 0 | 0.00458 | 0 | 0 | 0 | 0 | 0 | 0 | 0 | 0 | 0 |
| k__*Bacteria;* p__*Myxococcota;* c__*Myxococcia;* o__*Myxococcales;* f__*Anaeromyxobacteraceae;* g__*Anaeromyxobacter* | 2.775203 | CRAB | 2.3758 | 0.540291 | 0 | 0 | 0 | 0 | 0 | 0 | 0.003175 | 0 | 0 | 0 | 0 |
| k__*Bacteria;* p__*Myxococcota;* c__*Myxococcia;* o__*Myxococcales;* f__*Myxococcaceae;* g__*unidentified_Myxococcaceae* | 4.808184 | CREC | 4.560699 | 0.10247 | 0 | 0 | 0 | 0 | 0 | 0 | 0 | 0 | 0 | 0 | 0.055291 |
| k__*Bacteria;* p__*Myxococcota;* c__*Myxococcia;* o__*Myxococcales;* f__*Vulgatibacteraceae;* g__*Vulgatibacter* | 3.102174 | CRAB | 2.799272 | 0.540291 | 0 | 0.002586 | 0 | 0 | 0 | 0 | 0 | 0 | 0 | 0 | 0 |
| k__*Bacteria;* p__*Myxococcota;* c__*unidentified_Myxococcota;* o__*Haliangiales;* f__*Haliangiaceae;* g__*Haliangium* | 3.484791 | CRAB | 3.07094 | 0.363722 | 0 | 0.00486 | 0 | 0 | 0 | 0 | 0.003599 | 0 | 0 | 0 | 0 |
| k__*Bacteria;* p__*Myxococcota;* c__*unidentified_Myxococcota;* o__*Polyangiales;* f__*Polyangiaceae;* g__*Pajaroellobacter* | 3.830479 | CRAB | 3.417685 | 0.540291 | 0 | 0.013833 | 0 | 0 | 0 | 0 | 0 | 0 | 0 | 0 | 0 |
| k__*Bacteria;* p__*Myxococcota;* c__*unidentified_Myxococcota;* o__*Polyangiales;* f__*Polyangiaceae;* g__*Sorangium* | 4.047541 | CRAB | 3.826943 | 0.540291 | 0 | 0 | 0 | 0.038029 | 0 | 0 | 0 | 0 | 0 | 0 | 0 |
| k__*Bacteria;* p__*Nitrospirota;* c__*Nitrospiria;* o__*Nitrospirales;* f__*Nitrospiraceae;* g__*Nitrospira* | 3.236175 | CRAB | 2.876734 | 0.540291 | 0 | 0.003521 | 0 | 0 | 0 | 0 | 0 | 0 | 0 | 0 | 0 |
| k__*Bacteria;* p__*Planctomycetota;* c__*Phycisphaerae;* o__*Tepidisphaerales;* f__*unidentified_Tepidisphaerales;* g__*unidentified_Tepidisphaerales* | 2.525519 | CRAB | 2.141437 | 0.540291 | 0 | 0.000685 | 0 | 0 | 0 | 0 | 0 | 0 | 0 | 0 | 0 |
| k__*Bacteria;* p__*Proteobacteria;* c__*Alphaproteobacteria;* o__*Caulobacterales;* f__*Caulobacteraceae;* g__*Brevundimonas* | 4.081168 | CRAB | 3.808226 | 0.636328 | 0 | 0.000997 | 0 | 0.038828 | 0 | 0 | 0.000937 | 0 | 0 | 0 | 8.89E-05 |
| k__*Bacteria;* p__*Proteobacteria;* c__*Alphaproteobacteria;* o__*Caulobacterales;* f__*Caulobacteraceae;* g__*Phenylobacterium* | 3.873136 | CRAB | 3.623688 | 0.540291 | 0 | 0 | 0 | 0.025451 | 0 | 0 | 0 | 0 | 0 | 0 | 0 |
| k__*Bacteria;* p__*Proteobacteria;* c__*Alphaproteobacteria;* o__*Caulobacterales;* f__*Caulobacteraceae;* g__*unidentified_Caulobacteraceae* | 2.961247 | CRAB | 2.646193 | 0.540291 | 0 | 0.001869 | 0 | 0 | 0 | 0 | 0 | 0 | 0 | 0 | 0 |
| k__*Bacteria;* p__*Proteobacteria;* c__*Alphaproteobacteria;* o__*Caulobacterales;* f__*Hyphomonadaceae;* g__*Hirschia* | 3.424094 | CRAB | 3.218733 | 0.540291 | 0.007615 | 0 | 0 | 0 | 0 | 0 | 0 | 0 | 0 | 0 | 0 |
| k__*Bacteria;* p__*Proteobacteria;* c__*Alphaproteobacteria;* o__*Caulobacterales;* f__*Hyphomonadaceae;* g__*unidentified_Hyphomonadaceae* | 3.247554 | CRAB | 2.835787 | 0.540291 | 0 | 0.003614 | 0 | 0 | 0 | 0 | 0 | 0 | 0 | 0 | 0 |
| k__*Bacteria;* p__*Proteobacteria;* c__*Alphaproteobacteria;* o__*Micropepsales;* f__*Micropepsaceae;* g__*unidentified_Micropepsaceae* | 3.656115 | CRAB | 3.295413 | 0.363722 | 0 | 0.008412 | 0 | 0 | 0 | 0 | 0.002208 | 0 | 0 | 0 | 0 |
| k__*Bacteria;* p__*Proteobacteria;* c__*Alphaproteobacteria;* o__*Rhizobiales;* f__*Beijerinckiaceae;* g__*Roseiarcus* | 3.833267 | CRAB | 3.486105 | 0.363722 | 0 | 0.012774 | 0 | 0 | 0 | 0 | 0.002994 | 0 | 0 | 0 | 0 |
| k__*Bacteria;* p__*Proteobacteria;* c__*Alphaproteobacteria;* o__*Rhizobiales;* f__*Hyphomicrobiaceae;* g__*Hyphomicrobium* | 4.415921 | CREC | 4.12923 | 0.363722 | 0 | 0 | 0 | 0 | 0 | 0 | 0.007379 | 0 | 0 | 0.032711 | 0 |
| k__*Bacteria;* p__*Proteobacteria;* c__*Alphaproteobacteria;* o__*Rhizobiales;* f__*Xanthobacteraceae;* g__*Bradyrhizobium* | 3.80527 | CRAB | 3.420294 | 0.363722 | 0 | 0.009097 | 0 | 0 | 0 | 0 | 0.010312 | 0 | 0 | 0 | 0 |
| k__*Bacteria;* p__*Proteobacteria;* c__*Alphaproteobacteria;* o__*Rhizobiales;* f__*Xanthobacteraceae;* g__*Pseudorhodoplanes* | 3.728923 | CRAB | 3.533797 | 0.540291 | 0 | 0 | 0 | 0.01826 | 0 | 0 | 0 | 0 | 0 | 0 | 0 |
| k__*Bacteria;* p__*Proteobacteria;* c__*Alphaproteobacteria;* o__*Rhodobacterales;* f__*Rhodobacteraceae;* g__*Rhodobacter* | 2.272528 | CRAB | 1.936349 | 0.540291 | 0 | 0 | 0 | 0 | 0 | 0 | 0.000998 | 0 | 0 | 0 | 0 |
| k__*Bacteria;* p__*Proteobacteria;* c__*Alphaproteobacteria;* o__*Rhodobacterales;* f__*Rhodobacteraceae;* g__*Rubellimicrobium* | 3.398925 | CREC | 3.050491 | 0.10247 | 0 | 0 | 0 | 0 | 0 | 0 | 0 | 0 | 0.003795 | 0 | 0 |
| k__*Bacteria;* p__*Proteobacteria;* c__*Alphaproteobacteria;* o__*Rhodospirillales;* f__*Rhodospirillaceae;* g__*unidentified_Rhodospirillaceae* | 3.690119 | CRAB | 3.326151 | 0.363722 | 0 | 0.003552 | 0 | 0 | 0 | 0 | 0.016844 | 0 | 0 | 0 | 0 |
| k__*Bacteria;* p__*Proteobacteria;* c__*Alphaproteobacteria;* o__*Rickettsiales;* f__*unidentified_Rickettsiales;* g__*unidentified_Rickettsiales* | 2.946524 | CRAB | 2.575778 | 0.540291 | 0 | 0.001807 | 0 | 0 | 0 | 0 | 0 | 0 | 0 | 0 | 0 |
| k__*Bacteria;* p__*Proteobacteria;* c__*Alphaproteobacteria;* o__*Sphingomonadales;* f__*Sphingomonadaceae;* g__*Novosphingobium* | 4.225708 | CREC | 3.911413 | 0.795158 | 0 | 0.00162 | 0 | 0.029654 | 0 | 0 | 0 | 0 | 0.025468 | 0 | 0 |
| k__*Bacteria;* p__*Proteobacteria;* c__*Alphaproteobacteria;* o__*Sphingomonadales;* f__*Sphingomonadaceae;* g__*Sphingobium* | 3.796865 | CREC | 3.507727 | 0.544827 | 0 | 0 | 0 | 0 | 0 | 0.017558 | 0 | 0 | 0.009488 | 0 | 0 |
| k__*Bacteria;* p__*Proteobacteria;* c__*Alphaproteobacteria;* o__*Sphingomonadales;* f__*Sphingomonadaceae;* g__*Sphingomonas* | 3.95558 | CRAB | 3.662172 | 0.242692 | 0 | 0.007508 | 0 | 0.017727 | 0 | 0 | 0.000816 | 0 | 0 | 0 | 0 |
| k__*Bacteria;* p__*Proteobacteria;* c__*Alphaproteobacteria;* o__*unidentified_Alphaproteobacteria;* f__*Acetobacteraceae;* g__*Acidiphilium* | 2.456665 | CRAB | 2.299931 | 0.540291 | 0 | 0 | 0 | 0 | 0 | 0.001246 | 0 | 0 | 0 | 0 | 0 |
| k__*Bacteria;* p__*Proteobacteria;* c__*Alphaproteobacteria;* o__*unidentified_Alphaproteobacteria;* f__*Dongiaceae;* g__*Dongia* | 2.168987 | CRAB | 1.829886 | 0.540291 | 0 | 0 | 0 | 0 | 0 | 0 | 0.000786 | 0 | 0 | 0 | 0 |
| k__*Bacteria;* p__*Proteobacteria;* c__*Alphaproteobacteria;* o__*unidentified_Alphaproteobacteria;* f__*Reyranellaceae;* g__*Reyranella* | 3.086186 | CRAB | 2.758627 | 0.540291 | 0 | 0.002492 | 0 | 0 | 0 | 0 | 0 | 0 | 0 | 0 | 0 |
| k__*Bacteria;* p__*Proteobacteria;* c__*Alphaproteobacteria;* o__*unidentified_Alphaproteobacteria;* f__*unidentified_Alphaproteobacteria;* g__*unidentified_Alphaproteobacteria* | 3.273001 | CRAB | 2.900421 | 0.540291 | 0 | 0.003832 | 0 | 0 | 0 | 0 | 0 | 0 | 0 | 0 | 0 |
| k__*Bacteria;* p__*Proteobacteria;* c__*Gammaproteobacteria;* o__*Burkholderiales;* f__*Alcaligenaceae;* g__*Alcaligenes* | 4.47778 | CREC | 4.194106 | 0.10247 | 0 | 0 | 0 | 0 | 0 | 0 | 0 | 0 | 0 | 0.037719 | 0 |
| k__*Bacteria;* p__*Proteobacteria;* c__*Gammaproteobacteria;* o__*Burkholderiales;* f__*Burkholderiaceae;* g__*Cupriavidus* | 2.946524 | CRAB | 2.631719 | 0.540291 | 0 | 0.001807 | 0 | 0 | 0 | 0 | 0 | 0 | 0 | 0 | 0 |
| k__*Bacteria;* p__*Proteobacteria;* c__*Gammaproteobacteria;* o__*Burkholderiales;* f__*Burkholderiaceae;* g__*unidentified_Burkholderiaceae* | 2.845854 | CRAB | 2.517528 | 0.540291 | 0 | 0.001433 | 0 | 0 | 0 | 0 | 0 | 0 | 0 | 0 | 0 |
| k__*Bacteria;* p__*Proteobacteria;* c__*Gammaproteobacteria;* o__*Burkholderiales;* f__*Comamonadaceae;* g__*Acidovorax* | 4.539322 | CRAB | 4.179288 | 0.363722 | 0 | 0 | 0 | 0 | 0.061073 | 0.026426 | 0 | 0 | 0 | 0 | 0 |
| k__*Bacteria;* p__*Proteobacteria;* c__*Gammaproteobacteria;* o__*Burkholderiales;* f__*Comamonadaceae;* g__*Comamonas* | 2.185378 | CRAB | 1.820923 | 0.540291 | 0 | 0 | 0 | 0 | 0 | 0 | 0.000816 | 0 | 0 | 0 | 0 |
| k__*Bacteria;* p__*Proteobacteria;* c__*Gammaproteobacteria;* o__*Burkholderiales;* f__*Comamonadaceae;* g__*Ottowia* | 4.300835 | CREC | 3.953577 | 0.10247 | 0 | 0 | 0 | 0 | 0 | 0 | 0 | 0 | 0 | 0.025096 | 0 |
| k__*Bacteria;* p__*Proteobacteria;* c__*Gammaproteobacteria;* o__*Burkholderiales;* f__*Comamonadaceae;* g__*Rhizobacter* | 3.418625 | CRAB | 3.061745 | 0.540291 | 0 | 0.005359 | 0 | 0 | 0 | 0 | 0 | 0 | 0 | 0 | 0 |
| k__*Bacteria;* p__*Proteobacteria;* c__*Gammaproteobacteria;* o__*Burkholderiales;* f__*Comamonadaceae;* g__*Variovorax* | 4.014609 | CRAB | 3.66219 | 0.363722 | 0 | 0.018724 | 0 | 0 | 0 | 0 | 0.00629 | 0 | 0 | 0 | 0 |
| k__*Bacteria;* p__*Proteobacteria;* c__*Gammaproteobacteria;* o__*Burkholderiales;* f__*Oxalobacteraceae;* g__*Massilia* | 4.979099 | CRAB | 4.635462 | 0.520703 | 0.151314 | 0.001277 | 0 | 0.105386 | 0 | 0.000178 | 0 | 0.039983 | 0.057549 | 0 | 0 |
| k__*Bacteria;* p__*Proteobacteria;* c__*Gammaproteobacteria;* o__*Burkholderiales;* f__*Rhodocyclaceae;* g__*Dechloromonas* | 2.387482 | CRAB | 2.039977 | 0.540291 | 0 | 0 | 0 | 0 | 0 | 0 | 0.0013 | 0 | 0 | 0 | 0 |
| k__*Bacteria;* p__*Proteobacteria;* c__*Gammaproteobacteria;* o__*Enterobacterales;* f__*Aeromonadaceae;* g__*Aeromonas* | 3.755825 | CREC | 3.328116 | 0.026016 | 0 | 0 | 0 | 0 | 0 | 0.002373 | 0 | 0.001718 | 0.004536 | 0.0024 | 0.000682 |
| k__*Bacteria;* p__*Proteobacteria;* c__*Gammaproteobacteria;* o__*Enterobacterales;* f__*Enterobacteriaceae;* g__*Enterobacter* | 2.938063 | CRAB | 2.545889 | 0.795158 | 0.00237 | 0 | 0 | 0 | 0 | 0 | 0 | 0.000148 | 0.000949 | 0 | 0 |
| k__*Bacteria;* p__*Proteobacteria;* c__*Gammaproteobacteria;* o__*Enterobacterales;* f__*Enterobacteriaceae;* g__*Klebsiella* | 4.600849 | CRAB | 4.235121 | 0.363722 | 0 | 0 | 0 | 0 | 0 | 0 | 0.210409 | 0.001451 | 0 | 0 | 0 |
| k__*Bacteria;* p__*Proteobacteria;* c__*Gammaproteobacteria;* o__*Enterobacterales;* f__*Enterobacteriaceae;* g__*Raoultella* | 2.426112 | CRAB | 2.069865 | 0.540291 | 0 | 0 | 0 | 0 | 0 | 0 | 0.001421 | 0 | 0 | 0 | 0 |
| k__*Bacteria;* p__*Proteobacteria;* c__*Gammaproteobacteria;* o__*Enterobacterales;* f__*Enterobacteriaceae;* g__*Salmonella* | 3.130564 | CREC | 2.742892 | 0.363722 | 0 | 0 | 0 | 0.001391 | 0 | 0 | 0 | 0 | 0.002046 | 0 | 0 |
| k__*Bacteria;* p__*Proteobacteria;* c__*Gammaproteobacteria;* o__*Enterobacterales;* f__*Enterobacteriaceae;* g__*unidentified_Enterobacteriaceae* | 2.740395 | CRAB | 2.288017 | 0.656179 | 0 | 9.35E-05 | 0 | 0 | 0 | 0.000267 | 0.000544 | 0.001244 | 0.000356 | 0 | 0 |
| k__*Bacteria;* p__*Proteobacteria;* c__*Gammaproteobacteria;* o__*Pseudomonadales;* f__*Halieaceae;* g__*Halioglobus* | 2.231135 | CRAB | 1.868593 | 0.540291 | 0 | 0 | 0 | 0 | 0 | 0 | 0.000907 | 0 | 0 | 0 | 0 |
| k__*Bacteria;* p__*Proteobacteria;* c__*Gammaproteobacteria;* o__*Pseudomonadales;* f__*Moraxellaceae;* g__*Acinetobacter* | 4.253177 | CRAB | 3.997935 | 0.676053 | 0 | 0.001059 | 0 | 0.043445 | 0 | 0.018507 | 0.002117 | 0 | 0.000178 | 0.002252 | 5.93E-05 |
| k__*Bacteria;* p__*Proteobacteria;* c__*Gammaproteobacteria;* o__*Pseudomonadales;* f__*Moraxellaceae;* g__*Alkanindiges* | 2.665788 | CRAB | 2.383813 | 0.363722 | 0 | 0.000654 | 0 | 0 | 0 | 0.000623 | 0 | 0 | 0 | 0 | 0 |
| k__*Bacteria;* p__*Proteobacteria;* c__*Gammaproteobacteria;* o__*Pseudomonadales;* f__*Moraxellaceae;* g__*Enhydrobacter* | 4.56146 | CREC | 4.252337 | 0.010198 | 0 | 0 | 0 | 0 | 0 | 0.004983 | 0.00124 | 0.002517 | 0.006879 | 0.026104 | 0.009541 |
| k__*Bacteria;* p__*Proteobacteria;* c__*Gammaproteobacteria;* o__*Pseudomonadales;* f__*Moraxellaceae;* g__*Psychrobacter* | 2.810229 | CREC | 2.423288 | 0.10247 | 0 | 0 | 0 | 0 | 0 | 0 | 0 | 0 | 0.000978 | 0 | 0 |
| k__*Bacteria;* p__*Proteobacteria;* c__*Gammaproteobacteria;* o__*Pseudomonadales;* f__*Pseudomonadaceae;* g__*Pseudomonas* | 3.59938 |  |  |  | 0 | 0.003863 | 0 | 0 | 0 | 0.009076 | 0 | 0 | 0.002105 | 0 | 0 |
| k__*Bacteria;* p__*Proteobacteria;* c__*Gammaproteobacteria;* o__*Pseudomonadales;* f__*Spongiibacteraceae;* g__*unidentified_Spongiibacteraceae* | 2.134225 | CRAB | 1.78346 | 0.540291 | 0 | 0 | 0 | 0 | 0 | 0 | 0.000726 | 0 | 0 | 0 | 0 |
| k__*Bacteria;* p__*Proteobacteria;* c__*Gammaproteobacteria;* o__*Steroidobacterales;* f__*Steroidobacteraceae;* g__*Steroidobacter* | 3.258643 | CRAB | 2.92554 | 0.540291 | 0 | 0.003708 | 0 | 0 | 0 | 0 | 0 | 0 | 0 | 0 | 0 |
| k__*Bacteria;* p__*Proteobacteria;* c__*Gammaproteobacteria;* o__*unidentified_Gammaproteobacteria;* f__*unidentified_Gammaproteobacteria;* g__*Acidibacter* | 4.663079 | CREC | 4.37291 | 0.363722 | 0 | 0.02103 | 0 | 0 | 0 | 0 | 0 | 0 | 0 | 0 | 0.039586 |
| k__*Bacteria;* p__*Proteobacteria;* c__*Gammaproteobacteria;* o__*Xanthomonadales;* f__*Rhodanobacteraceae;* g__*Dyella* | 3.443168 | CRAB | 3.116761 | 0.540291 | 0 | 0.00567 | 0 | 0 | 0 | 0 | 0 | 0 | 0 | 0 | 0 |
| k__*Bacteria;* p__*Proteobacteria;* c__*Gammaproteobacteria;* o__*Xanthomonadales;* f__*Rhodanobacteraceae;* g__*Rhodanobacter* | 3.802189 | CRAB | 3.463243 | 0.540291 | 0 | 0.012961 | 0 | 0 | 0 | 0 | 0 | 0 | 0 | 0 | 0 |
| k__*Bacteria;* p__*Proteobacteria;* c__*Gammaproteobacteria;* o__*Xanthomonadales;* f__*Xanthomonadaceae;* g__*Arenimonas* | 2.285493 | CRAB | 1.962914 | 0.540291 | 0 | 0 | 0 | 0 | 0 | 0 | 0.001028 | 0 | 0 | 0 | 0 |
| k__*Bacteria;* p__*Proteobacteria;* c__*Gammaproteobacteria;* o__*Xanthomonadales;* f__*Xanthomonadaceae;* g__*Stenotrophomonas* | 2.740152 | CRAB | 2.401304 | 0.363722 | 0 | 0.000312 | 0 | 0 | 0 | 0 | 0.002117 | 0 | 0 | 0 | 0 |
| k__*Bacteria;* p__*Spirochaetota;* c__*Spirochaetia;* o__*Spirochaetales;* f__*Spirochaetaceae;* g__*Treponema* | 3.703765 | CRAB | 3.342624 | 0.540291 | 0 | 0 | 0 | 0 | 0 | 0 | 0 | 0.018452 | 0 | 0 | 0 |
| k__*Bacteria;* p__*Thermotogota;* c__*Thermotogae;* o__*Petrotogales;* f__*Petrotogaceae;* g__*Defluviitoga* | 3.442031 | CRAB | 3.079636 | 0.15332 | 0 | 0 | 0 | 0 | 0 | 0 | 0 | 0.0101 | 0.002283 | 0 | 5.93E-05 |
| k__*Bacteria;* p__*Verrucomicrobiota;* c__*Verrucomicrobiae;* o__*Chthoniobacterales;* f__*Chthoniobacteraceae;* g__*Chthoniobacter* | 3.069587 | CRAB | 2.72899 | 0.540291 | 0 | 0.002399 | 0 | 0 | 0 | 0 | 0 | 0 | 0 | 0 | 0 |
| k__*Bacteria;* p__*Verrucomicrobiota;* c__*Verrucomicrobiae;* o__*Methylacidiphilales;* f__*Methylacidiphilaceae;* g__*unidentified_Methylacidiphilaceae* | 2.645494 | CRAB | 2.274487 | 0.540291 | 0 | 0.000904 | 0 | 0 | 0 | 0 | 0 | 0 | 0 | 0 | 0 |
| k__*Bacteria;* p__*Verrucomicrobiota;* c__*Verrucomicrobiae;* o__*unidentified_Verrucomicrobiae;* f__*unidentified_Verrucomicrobiae;* g__*Pedosphaera* | 3.00264 | CRAB | 2.662907 | 0.540291 | 0 | 0.002056 | 0 | 0 | 0 | 0 | 0 | 0 | 0 | 0 | 0 |
| k__*Bacteria;* p__*Acidobacteriota;* c__*Acidobacteriae;* o__*Bryobacterales;* f__*Bryobacteraceae;* g__*Bryobacter;* s__*metagenome* | 3.453731 | CRAB | 3.181075 | 0.540291 | 0 | 0 | 0 | 0 | 0 | 0 | 0.012005 | 0 | 0 | 0 | 0 |
| k__*Bacteria;* p__*Acidobacteriota;* c__*unidentified_Acidobacteriota;* o__*unidentified_Acidobacteriota;* f__*unidentified_Acidobacteriota;* g__*unidentified_Acidobacteriota;* s__*metagenome* | 4.603873 | CRAB | 4.357202 | 0.363722 | 0 | 0.020937 | 0 | 0 | 0 | 0 | 0.013426 | 0 | 0 | 0 | 0 |
| k__*Bacteria;* p__*Acidobacteriota;* c__*Vicinamibacteria;* o__*Vicinamibacterales;* f__*unidentified_Vicinamibacterales;* g__*unidentified_Vicinamibacterales;* s__*metagenome* | 3.629311 | CRAB | 3.208814 | 0.540291 | 0 | 0 | 0 | 0.004913 | 0 | 0 | 0 | 0 | 0 | 0 | 0 |
| k__*Bacteria;* p__*Actinobacteria;* c__*unidentified_Actinobacteria;* o__*Catenulisporales;* f__*Catenulisporaceae;* g__*Catenulispora;* s__*Catenulispora_rubra* | 3.083116 | CRAB | 2.848455 | 0.540291 | 0 | 0.000685 | 0 | 0 | 0 | 0 | 0 | 0 | 0 | 0 | 0 |
| k__*Bacteria;* p__*Actinobacteria;* c__*unidentified_Actinobacteria;* o__*Corynebacteriales;* f__*Corynebacteriaceae;* g__*Corynebacterium;* s__*Corynebacterium_tuberculostearicum* | 4.297422 | CRAB | 4.049434 | 0.540291 | 0.0152 | 0 | 0 | 0 | 0 | 0 | 0 | 0 | 0 | 0 | 0 |
| k__*Bacteria;* p__*Actinobacteria;* c__*unidentified_Actinobacteria;* o__*Micrococcales;* f__*Micrococcaceae;* g__*Arthrobacter;* s__*Arthrobacter_agilis* | 4.302117 | CREC | 3.980989 | 0.10247 | 0 | 0 | 0 | 0 | 0 | 0 | 0 | 0 | 0 | 0.013511 | 0 |
| k__*Bacteria;* p__*Actinobacteria;* c__*unidentified_Actinobacteria;* o__*Micrococcales;* f__*Micrococcaceae;* g__*Arthrobacter;* s__*Arthrobacter_crystallopoietes* | 4.527322 | CRAB | 4.316407 | 0.540291 | 0.025807 | 0 | 0 | 0 | 0 | 0 | 0 | 0 | 0 | 0 | 0 |
| k__*Bacteria;* p__*Actinobacteria;* c__*unidentified_Actinobacteria;* o__*Micrococcales;* f__*Micrococcaceae;* g__*Micrococcus;* s__*Micrococcus_luteus* | 4.788701 | CRAB | 4.54901 | 0.544827 | 0.04711 | 0 | 0 | 0 | 0 | 0 | 0 | 0 | 0 | 0.003141 | 0 |
| k__*Bacteria;* p__*Actinobacteria;* c__*unidentified_Actinobacteria;* o__*Propionibacteriales;* f__*Nocardioidaceae;* g__*Nocardioides;* s__*Nocardioides_sp* | 3.511545 | CRAB | 3.271655 | 0.544827 | 0 | 0.001838 | 0 | 0 | 0 | 0 | 0 | 0 | 0 | 0 | 0.000267 |
| k__*Bacteria;* p__*Actinobacteria;* c__*unidentified_Actinobacteria;* o__*Propionibacteriales;* f__*Propionibacteriaceae;* g__*Cutibacterium;* s__*Cutibacterium_acnes* | 5.079093 | CREC | 4.735141 | 0.094638 | 5.93E-05 | 0.003053 | 0 | 0 | 0 | 0.005457 | 6.05E-05 | 0 | 0.002698 | 0.076415 | 0.000119 |
| k__*Bacteria;* p__*Actinobacteriota;* c__*Acidimicrobiia;* o__*unidentified_Acidimicrobiia;* f__*unidentified_Acidimicrobiia;* g__*unidentified_Acidimicrobiia;* s__*metagenome* | 4.1684 | CRAB | 3.825314 | 0.363722 | 0 | 0.004798 | 0 | 0.007221 | 0 | 0 | 0 | 0 | 0 | 0 | 0 |
| k__*Bacteria;* p__*Actinobacteriota;* c__*Coriobacteriia;* o__*Coriobacteriales;* f__*Eggerthellaceae;* g__*Slackia;* s__*Slackia_sp_NATTS* | 3.506902 | CRAB | 3.223124 | 0.540291 | 0 | 0 | 0 | 0 | 0 | 0.005635 | 0 | 0 | 0 | 0 | 0 |
| k__*Bacteria;* p__*Bacteroidota;* c__*Bacteroidia;* o__*Bacteroidales;* f__*Bacteroidaceae;* g__*Bacteroides;* s__*Bacteroides_coprocola* | 3.863574 | CRAB | 3.60051 | 0.540291 | 0 | 0 | 0.00287 | 0 | 0 | 0 | 0 | 0 | 0 | 0 | 0 |
| k__*Bacteria;* p__*Bacteroidota;* c__*Bacteroidia;* o__*Bacteroidales;* f__*Bacteroidaceae;* g__*Bacteroides;* s__*Bacteroides_fragilis* | 4.362149 | CRAB | 3.932407 | 0.540291 | 0 | 0 | 0 | 0 | 0 | 0 | 0 | 0.031306 | 0 | 0 | 0 |
| k__*Bacteria;* p__*Bacteroidota;* c__*Bacteroidia;* o__*Bacteroidales;* f__*Porphyromonadaceae;* g__*Porphyromonas;* s__*Porphyromonas_sp_canine_oral_taxon_366* | 4.055574 | CREC | 3.752233 | 0.10247 | 0 | 0 | 0 | 0 | 0 | 0 | 0 | 0 | 0.005129 | 0 | 0 |
| k__*Bacteria;* p__*Bacteroidota;* c__*Bacteroidia;* o__*Bacteroidales;* f__*Prevotellaceae;* g__*unidentified_Prevotellaceae;* s__*metagenome* | 3.780769 | CRAB | 3.517223 | 0.363722 | 0 | 0 | 0 | 0 | 0 | 0.008868 | 0 | 0.001333 | 0 | 0 | 0 |
| k__*Bacteria;* p__*Bacteroidota;* c__*Bacteroidia;* o__*Bacteroidales;* f__*Rikenellaceae;* g__*unidentified_Rikenellaceae;* s__*metagenome* | 3.985544 | CRAB | 3.566602 | 0.540291 | 0 | 0 | 0 | 0.011157 | 0 | 0 | 0 | 0 | 0 | 0 | 0 |
| k__*Bacteria;* p__*Bacteroidota;* c__*Bacteroidia;* o__*Bacteroidales;* f__*Tannerellaceae;* g__*Parabacteroides;* s__*Parabacteroides_sp_CT06* | 3.921273 | CRAB | 3.524495 | 0.540291 | 0 | 0 | 0 | 0 | 0 | 0 | 0 | 0.011343 | 0 | 0 | 0 |
| k__*Bacteria;* p__*Bacteroidota;* c__*Bacteroidia;* o__*Flavobacteriales;* f__*Flavobacteriaceae;* g__*Myroides;* s__*Myroides_phaeus* | 3.497661 | CRAB | 3.245242 | 0.540291 | 0 | 0 | 0 | 0 | 0 | 0.005517 | 0 | 0 | 0 | 0 | 0 |
| k__*Bacteria;* p__*Cyanobacteria;* c__*Sericytochromatia;* o__*unidentified_Sericytochromatia;* f__*unidentified_Sericytochromatia;* g__*unidentified_Sericytochromatia;* s__*metagenome* | 3.083116 | CRAB | 2.811461 | 0.540291 | 0 | 0.000685 | 0 | 0 | 0 | 0 | 0 | 0 | 0 | 0 | 0 |
| k__*Bacteria;* p__*Cyanobacteria;* c__*unidentified_Cyanobacteria;* o__*Leptolyngbyales;* f__*Leptolyngbyaceae;* g__*unidentified_Leptolyngbyaceae;* s__*Phormidium_sp_IAM_M_99* | 4.304667 | CREC | 4.016472 | 0.10247 | 0 | 0 | 0 | 0 | 0 | 0 | 0 | 0 | 0.009102 | 0 | 0 |
| k__*Bacteria;* p__*Cyanobacteria;* c__*unidentified_Cyanobacteria;* o__*unidentified_Cyanobacteria;* f__*Nostocaceae;* g__*unidentified_Nostocaceae;* s__*Calothrix_sp_PCC_7103* | 4.64035 | CREC | 4.391054 | 0.10247 | 0 | 0 | 0 | 0 | 0 | 0 | 0 | 0 | 0.019717 | 0 | 0 |
| k__*Bacteria;* p__*Deinococcota;* c__*Deinococci;* o__*Deinococcales;* f__*Deinococcaceae;* g__*Deinococcus;* s__*Deinococcus_sp* | 4.581704 | CREC | 4.325949 | 0.10247 | 0 | 0 | 0 | 0 | 0 | 0 | 0 | 0 | 0.017226 | 0 | 0 |
| k__*Bacteria;* p__*Firmicutes;* c__*Bacilli;* o__*Bacillales;* f__*Bacillaceae;* g__*Bacillus;* s__*Bacillus_anthracis* | 4.494039 | CRAB | 4.179011 | 0.795158 | 0 | 0 | 0 | 0 | 0 | 0.045615 | 0 | 0.007049 | 0.008065 | 0 | 0 |
| k__*Bacteria;* p__*Firmicutes;* c__*Bacilli;* o__*Bacillales;* f__*Bacillaceae;* g__*Bacillus;* s__*Bacillus_aryabhattai* | 4.219458 | CRAB | 3.804368 | 0.540291 | 0 | 0 | 0 | 0 | 0 | 0 | 0 | 0.022539 | 0 | 0 | 0 |
| k__*Bacteria;* p__*Firmicutes;* c__*Bacilli;* o__*Bacillales;* f__*Planococcaceae;* g__*Sporosarcina;* s__*Sporosarcina_psychrophila* | 3.412791 | CRAB | 3.162128 | 0.540291 | 0 | 0.001464 | 0 | 0 | 0 | 0 | 0 | 0 | 0 | 0 | 0 |
| k__*Bacteria;* p__*Firmicutes;* c__*Bacilli;* o__*Lactobacillales;* f__*Enterococcaceae;* g__*Enterococcus;* s__*Enterococcus_faecalis* | 4.115765 | CRAB | 3.883776 | 0.540291 | 0 | 0 | 0 | 0 | 0 | 0.022896 | 0 | 0 | 0 | 0 | 0 |
| k__*Bacteria;* p__*Firmicutes;* c__*Bacilli;* o__*Lactobacillales;* f__*Enterococcaceae;* g__*Enterococcus;* s__*Enterococcus_faecium* | 4.933395 | CRAB | 4.644247 | 0.242692 | 0 | 0 | 0 | 0 | 0 | 0.008898 | 0.273882 | 0.021561 | 0 | 0 | 0 |
| k__*Bacteria;* p__*Firmicutes;* c__*Bacilli;* o__*Lactobacillales;* f__*Enterococcaceae;* g__*Melissococcus;* s__*Melissococcus_plutonius* | 4.519959 | CREC | 4.264412 | 0.10247 | 0 | 0 | 0 | 0 | 0 | 0 | 0 | 0 | 0.014943 | 0 | 0 |
| k__*Bacteria;* p__*Firmicutes;* c__*Bacilli;* o__*Lactobacillales;* f__*Lactobacillaceae;* g__*Lacticaseibacillus;* s__*Lactobacillus_paracasei* | 3.568592 | CRAB | 3.306545 | 0.540291 | 0 | 0 | 0 | 0 | 0 | 0.006495 | 0 | 0 | 0 | 0 | 0 |
| k__*Bacteria;* p__*Firmicutes;* c__*Bacilli;* o__*Lactobacillales;* f__*Lactobacillaceae;* g__*Lactobacillus;* s__*Lactobacillus_homohiochii* | 4.133499 | CREC | 3.771977 | 0.363722 | 0 | 0 | 0 | 0 | 0 | 0.004983 | 0 | 0 | 0.006137 | 0 | 0 |
| k__*Bacteria;* p__*Firmicutes;* c__*Bacilli;* o__*Lactobacillales;* f__*Lactobacillaceae;* g__*Lactobacillus;* s__*Lactobacillus_johnsonii* | 4.739654 |  |  |  | 0 | 0 | 0.02154 | 2.96E-05 | 0 | 0 | 0 | 8.89E-05 | 0 | 0 | 0.000563 |
| k__*Bacteria;* p__*Firmicutes;* c__*Bacilli;* o__*Lactobacillales;* f__*Lactobacillaceae;* g__*Ligilactobacillus;* s__*Lactobacillus_aviarius* | 4.686907 | CREC | 4.395379 | 0.10247 | 0 | 0 | 0 | 0 | 0 | 0 | 0 | 0 | 0 | 0.03277 | 0 |
| k__*Bacteria;* p__*Firmicutes;* c__*Bacilli;* o__*Lactobacillales;* f__*Lactobacillaceae;* g__*Ligilactobacillus;* s__*Lactobacillus_murinus* | 4.333402 | CREC | 4.017897 | 0.795158 | 0 | 0 | 0 | 0.000474 | 0 | 0 | 0 | 0.022095 | 0.009725 | 0 | 0 |
| k__*Bacteria;* p__*Firmicutes;* c__*Bacilli;* o__*Lactobacillales;* f__*Lactobacillaceae;* g__*Ligilactobacillus;* s__*Lactobacillus_salivarius* | 4.009903 | CRAB | 3.752249 | 0.540291 | 0 | 0 | 0 | 0 | 0 | 0.017943 | 0 | 0 | 0 | 0 | 0 |
| k__*Bacteria;* p__*Firmicutes;* c__*Bacilli;* o__*Lactobacillales;* f__*Lactobacillaceae;* g__*Weissella;* s__*Weissella_cibaria* | 4.002489 | CRAB | 3.611178 | 0.540291 | 0 | 0 | 0 | 0.011601 | 0 | 0 | 0 | 0 | 0 | 0 | 0 |
| k__*Bacteria;* p__*Firmicutes;* c__*Bacilli;* o__*Lactobacillales;* f__*Streptococcaceae;* g__*Streptococcus;* s__*Streptococcus_equinus* | 4.710179 | CREC | 4.429922 | 0.363722 | 0 | 0 | 0 | 0 | 0 | 0 | 0 | 0.013387 | 0.023156 | 0 | 0 |
| k__*Bacteria;* p__*Firmicutes;* c__*Bacilli;* o__*Lactobacillales;* f__*Streptococcaceae;* g__*Streptococcus;* s__*Streptococcus_pneumoniae* | 3.834529 | CRAB | 3.584526 | 0.540291 | 0 | 0 | 0 | 0 | 0 | 0.011982 | 0 | 0 | 0 | 0 | 0 |
| k__*Bacteria;* p__*Firmicutes;* c__*Bacilli;* o__*unidentified_Bacilli;* f__*Staphylococcaceae;* g__*Staphylococcus;* s__*Staphylococcus_epidermidis* | 4.798488 | CRAB | 4.504291 | 0.540291 | 0 | 0 | 0.024706 | 0 | 0 | 0 | 0 | 0 | 0 | 0 | 0 |
| k__*Bacteria;* p__*Firmicutes;* c__*Bacilli;* o__*unidentified_Bacilli;* f__*unidentified_Bacilli;* g__*Aneurinibacillus;* s__*Aneurinibacillus_soli* | 3.729009 | CRAB | 3.296522 | 0.540291 | 0 | 0 | 0 | 0 | 0 | 0 | 0 | 0.007286 | 0 | 0 | 0 |
| k__*Bacteria;* p__*Firmicutes;* c__*Clostridia;* o__*Clostridiales;* f__*Clostridiaceae;* g__*unidentified_Clostridiaceae;* s__*metagenome* | 3.929716 | CRAB | 3.679219 | 0.540291 | 0 | 0 | 0 | 0 | 0 | 0.014918 | 0 | 0 | 0 | 0 | 0 |
| k__*Bacteria;* p__*Firmicutes;* c__*Clostridia;* o__*Lachnospirales;* f__*Lachnospiraceae;* g__*Anaerostipes;* s__*Anaerostipes_hadrus* | 2.550367 | CRAB | 2.345063 | 0.540291 | 0 | 0 | 0 | 0 | 0 | 0.000623 | 0 | 0 | 0 | 0 | 0 |
| k__*Bacteria;* p__*Firmicutes;* c__*Clostridia;* o__*Lachnospirales;* f__*Lachnospiraceae;* g__*unidentified_Lachnospiraceae;* s__*Clostridiales_bacterium_CIEAF_020* | 3.72724 | CRAB | 3.310524 | 0.540291 | 0 | 0 | 0 | 0 | 0 | 0 | 0 | 0.007256 | 0 | 0 | 0 |
| k__*Bacteria;* p__*Firmicutes;* c__*Clostridia;* o__*Lachnospirales;* f__*Lachnospiraceae;* g__*unidentified_Lachnospiraceae;* s__*Lachnospiraceae_bacterium_GAM79* | 3.502306 | CRAB | 3.273037 | 0.540291 | 0 | 0 | 0 | 0 | 0 | 0.005576 | 0 | 0 | 0 | 0 | 0 |
| k__*Bacteria;* p__*Firmicutes;* c__*Clostridia;* o__*unidentified_Clostridia;* f__*Peptostreptococcaceae;* g__*Filifactor;* s__*Filifactor_sp_canine_oral_taxon_064* | 4.191464 | CRAB | 3.970873 | 0.540291 | 0 | 0 | 0 | 0 | 0 | 0.027256 | 0 | 0 | 0 | 0 | 0 |
| k__*Bacteria;* p__*Firmicutes;* c__*Clostridia;* o__*unidentified_Clostridia;* f__*unidentified_Clostridia;* g__*Finegoldia;* s__*Finegoldia_magna* | 2.317338 | CRAB | 2.0615 | 0.540291 | 0 | 0 | 0 | 0 | 0 | 0 | 0.000877 | 0 | 0 | 0 | 0 |
| k__*Bacteria;* p__*Firmicutes;* c__*Negativicutes;* o__*unidentified_Negativicutes;* f__*Veillonellaceae;* g__*Dialister;* s__*gut_metagenome* | 4.136724 | CRAB | 3.743988 | 0.540291 | 0 | 0 | 0 | 0 | 0 | 0 | 0 | 0.018629 | 0 | 0 | 0 |
| k__*Bacteria;* p__*Gemmatimonadota;* c__*Gemmatimonadetes;* o__*Gemmatimonadales;* f__*Gemmatimonadaceae;* g__*unidentified_Gemmatimonadaceae;* s__*metagenome* | 3.90801 | CRAB | 3.635112 | 0.540291 | 0 | 0.00458 | 0 | 0 | 0 | 0 | 0 | 0 | 0 | 0 | 0 |
| k__*Bacteria;* p__*Myxococcota;* c__*Myxococcia;* o__*Myxococcales;* f__*Myxococcaceae;* g__*unidentified_Myxococcaceae;* s__*metagenome* | 5.446851 | CREC | 5.084784 | 0.10247 | 0 | 0 | 0 | 0 | 0 | 0 | 0 | 0 | 0 | 0 | 0.055291 |
| k__*Bacteria;* p__*Myxococcota;* c__*unidentified_Myxococcota;* o__*Polyangiales;* f__*Polyangiaceae;* g__*Sorangium;* s__*Sorangium_cellulosum* | 4.518106 | CRAB | 4.053587 | 0.540291 | 0 | 0 | 0 | 0.038029 | 0 | 0 | 0 | 0 | 0 | 0 | 0 |
| k__*Bacteria;* p__*Nitrospirota;* c__*Nitrospiria;* o__*Nitrospirales;* f__*Nitrospiraceae;* g__*Nitrospira;* s__*metagenome* | 3.504121 | CRAB | 3.243265 | 0.540291 | 0 | 0.001807 | 0 | 0 | 0 | 0 | 0 | 0 | 0 | 0 | 0 |
| k__*Bacteria;* p__*Planctomycetota;* c__*Phycisphaerae;* o__*Tepidisphaerales;* f__*unidentified_Tepidisphaerales;* g__*unidentified_Tepidisphaerales;* s__*metagenome* | 3.083116 | CRAB | 2.838369 | 0.540291 | 0 | 0.000685 | 0 | 0 | 0 | 0 | 0 | 0 | 0 | 0 | 0 |
| k__*Bacteria;* p__*Proteobacteria;* c__*Alphaproteobacteria;* o__*Caulobacterales;* f__*Caulobacteraceae;* g__*Brevundimonas;* s__*Brevundimonas_intermedia* | 4.551999 | CRAB | 4.108398 | 0.636328 | 0 | 0.000997 | 0 | 0.038828 | 0 | 0 | 0.000937 | 0 | 0 | 0 | 8.89E-05 |
| k__*Bacteria;* p__*Proteobacteria;* c__*Alphaproteobacteria;* o__*Caulobacterales;* f__*Caulobacteraceae;* g__*unidentified_Caulobacteraceae;* s__*metagenome* | 3.518844 | CRAB | 3.244925 | 0.540291 | 0 | 0.001869 | 0 | 0 | 0 | 0 | 0 | 0 | 0 | 0 | 0 |
| k__*Bacteria;* p__*Proteobacteria;* c__*Alphaproteobacteria;* o__*Caulobacterales;* f__*Hyphomonadaceae;* g__*Hirschia;* s__*metagenome* | 3.997237 | CRAB | 3.776425 | 0.540291 | 0.007615 | 0 | 0 | 0 | 0 | 0 | 0 | 0 | 0 | 0 | 0 |
| k__*Bacteria;* p__*Proteobacteria;* c__*Alphaproteobacteria;* o__*Caulobacterales;* f__*Hyphomonadaceae;* g__*unidentified_Hyphomonadaceae;* s__*metagenome* | 3.805151 | CRAB | 3.530075 | 0.540291 | 0 | 0.003614 | 0 | 0 | 0 | 0 | 0 | 0 | 0 | 0 | 0 |
| k__*Bacteria;* p__*Proteobacteria;* c__*Alphaproteobacteria;* o__*Micropepsales;* f__*Micropepsaceae;* g__*unidentified_Micropepsaceae;* s__*metagenome* | 4.187069 | CRAB | 3.925025 | 0.363722 | 0 | 0.008412 | 0 | 0 | 0 | 0 | 0.002208 | 0 | 0 | 0 | 0 |
| k__*Bacteria;* p__*Proteobacteria;* c__*Alphaproteobacteria;* o__*Rhizobiales;* f__*Hyphomicrobiaceae;* g__*Hyphomicrobium;* s__*metagenome* | 4.686121 | CREC | 4.39689 | 0.10247 | 0 | 0 | 0 | 0 | 0 | 0 | 0 | 0 | 0 | 0.032711 | 0 |
| k__*Bacteria;* p__*Proteobacteria;* c__*Alphaproteobacteria;* o__*Rhodospirillales;* f__*Rhodospirillaceae;* g__*unidentified_Rhodospirillaceae;* s__*metagenome* | 4.01128 | CRAB | 3.756904 | 0.363722 | 0 | 0.003552 | 0 | 0 | 0 | 0 | 0.016844 | 0 | 0 | 0 | 0 |
| k__*Bacteria;* p__*Proteobacteria;* c__*Alphaproteobacteria;* o__*Rickettsiales;* f__*unidentified_Rickettsiales;* g__*unidentified_Rickettsiales;* s__*metagenome* | 3.504121 | CRAB | 3.277631 | 0.540291 | 0 | 0.001807 | 0 | 0 | 0 | 0 | 0 | 0 | 0 | 0 | 0 |
| k__*Bacteria;* p__*Proteobacteria;* c__*Alphaproteobacteria;* o__*Sphingomonadales;* f__*Sphingomonadaceae;* g__*Novosphingobium;* s__*metagenome* | 3.456696 | CRAB | 3.212749 | 0.540291 | 0 | 0.00162 | 0 | 0 | 0 | 0 | 0 | 0 | 0 | 0 | 0 |
| k__*Bacteria;* p__*Proteobacteria;* c__*Alphaproteobacteria;* o__*unidentified_Alphaproteobacteria;* f__*unidentified_Alphaproteobacteria;* g__*unidentified_Alphaproteobacteria;* s__*metagenome* | 3.830598 | CRAB | 3.591452 | 0.540291 | 0 | 0.003832 | 0 | 0 | 0 | 0 | 0 | 0 | 0 | 0 | 0 |
| k__*Bacteria;* p__*Proteobacteria;* c__*Gammaproteobacteria;* o__*Burkholderiales;* f__*Alcaligenaceae;* g__*Alcaligenes;* s__*Alcaligenes_faecalis* | 4.747981 | CREC | 4.41625 | 0.10247 | 0 | 0 | 0 | 0 | 0 | 0 | 0 | 0 | 0 | 0.037719 | 0 |
| k__*Bacteria;* p__*Proteobacteria;* c__*Gammaproteobacteria;* o__*Burkholderiales;* f__*Burkholderiaceae;* g__*unidentified_Burkholderiaceae;* s__*Paraburkholderia_sp* | 3.403451 | CRAB | 3.150202 | 0.540291 | 0 | 0.001433 | 0 | 0 | 0 | 0 | 0 | 0 | 0 | 0 | 0 |
| k__*Bacteria;* p__*Proteobacteria;* c__*Gammaproteobacteria;* o__*Burkholderiales;* f__*Comamonadaceae;* g__*Comamonas;* s__*Comamonas_testosteroni* | 2.286304 | CRAB | 2.009104 | 0.540291 | 0 | 0 | 0 | 0 | 0 | 0 | 0.000816 | 0 | 0 | 0 | 0 |
| k__*Bacteria;* p__*Proteobacteria;* c__*Gammaproteobacteria;* o__*Burkholderiales;* f__*Oxalobacteraceae;* g__*Massilia;* s__*Massilia_armeniaca* | 4.779424 | CREC | 4.495318 | 0.10247 | 0 | 0 | 0 | 0 | 0 | 0 | 0 | 0 | 0.027158 | 0 | 0 |
| k__*Bacteria;* p__*Proteobacteria;* c__*Gammaproteobacteria;* o__*Enterobacterales;* f__*Aeromonadaceae;* g__*Aeromonas;* s__*Aeromonas_caviae* | 2.935718 | CRAB | 2.714715 | 0.540291 | 0 | 0 | 0 | 0 | 0 | 0.001513 | 0 | 0 | 0 | 0 | 0 |
| k__*Bacteria;* p__*Proteobacteria;* c__*Gammaproteobacteria;* o__*Enterobacterales;* f__*Aeromonadaceae;* g__*Aeromonas;* s__*Aeromonas_hydrophila* | 3.058357 | CRAB | 2.712135 | 0.363722 | 0 | 0 | 0 | 0 | 0 | 0.00086 | 0 | 0.000889 | 0 | 0 | 0 |
| k__*Bacteria;* p__*Proteobacteria;* c__*Gammaproteobacteria;* o__*Enterobacterales;* f__*Enterobacteriaceae;* g__*Klebsiella;* s__*Klebsiella_pneumoniae* | 4.526946 | CRAB | 4.261871 | 0.540291 | 0 | 0 | 0 | 0 | 0 | 0 | 0.142097 | 0 | 0 | 0 | 0 |
| k__*Bacteria;* p__*Proteobacteria;* c__*Gammaproteobacteria;* o__*Enterobacterales;* f__*Enterobacteriaceae;* g__*Klebsiella;* s__*Klebsiella_variicola* | 4.170291 | CRAB | 3.876108 | 0.363722 | 0 | 0 | 0 | 0 | 0 | 0 | 0.058 | 0.001451 | 0 | 0 | 0 |
| k__*Bacteria;* p__*Proteobacteria;* c__*Gammaproteobacteria;* o__*Enterobacterales;* f__*Enterobacteriaceae;* g__*unidentified_Enterobacteriaceae;* s__*Escherichia_coli* | 3.133858 | CRAB | 2.637454 | 0.823849 | 0 | 9.35E-05 | 0 | 0 | 0 | 0.000267 | 0.000544 | 0.001244 | 0.000356 | 0 | 0 |
| k__*Bacteria;* p__*Proteobacteria;* c__*Gammaproteobacteria;* o__*Pseudomonadales;* f__*Moraxellaceae;* g__*Acinetobacter;* s__*Acinetobacter_baumannii* | 3.344227 | CRAB | 3.099599 | 0.363722 | 0 | 0 | 0 | 0 | 0 | 0.002996 | 0.002117 | 0 | 0 | 0 | 0 |
| k__*Bacteria;* p__*Proteobacteria;* c__*Gammaproteobacteria;* o__*Pseudomonadales;* f__*Moraxellaceae;* g__*Acinetobacter;* s__*Acinetobacter_johnsonii* | 3.963967 | CRAB | 3.565421 | 0.478164 | 0 | 0 | 0 | 0.003906 | 0 | 0.010203 | 0 | 0 | 0.000148 | 0.002252 | 0 |
| k__*Bacteria;* p__*Proteobacteria;* c__*Gammaproteobacteria;* o__*Pseudomonadales;* f__*Moraxellaceae;* g__*Acinetobacter;* s__*Acinetobacter_junii* | 3.272172 | CRAB | 3.016009 | 0.540291 | 0 | 0.001059 | 0 | 0 | 0 | 0 | 0 | 0 | 0 | 0 | 0 |
| k__*Bacteria;* p__*Proteobacteria;* c__*Gammaproteobacteria;* o__*Pseudomonadales;* f__*Moraxellaceae;* g__*Acinetobacter;* s__*Acinetobacter_schindleri* | 4.385553 | CRAB | 3.923046 | 0.540291 | 0 | 0 | 0 | 0.028026 | 0 | 0 | 0 | 0 | 0 | 0 | 0 |
| k__*Bacteria;* p__*Proteobacteria;* c__*Gammaproteobacteria;* o__*Pseudomonadales;* f__*Moraxellaceae;* g__*Acinetobacter;* s__*Acinetobacter_sp_WCHAc010034* | 3.167667 | CRAB | 2.920345 | 0.540291 | 0 | 0 | 0 | 0 | 0 | 0.00258 | 0 | 0 | 0 | 0 | 0 |
| k__*Bacteria;* p__*Proteobacteria;* c__*Gammaproteobacteria;* o__*Pseudomonadales;* f__*Moraxellaceae;* g__*Enhydrobacter;* s__*Moraxella_osloensis* | 5.009712 | CREC | 4.652045 | 0.010198 | 0 | 0 | 0 | 0 | 0 | 0.004983 | 0.00124 | 0.002517 | 0.006879 | 0.026104 | 0.009541 |
| k__*Bacteria;* p__*Proteobacteria;* c__*Gammaproteobacteria;* o__*Pseudomonadales;* f__*Pseudomonadaceae;* g__*Pseudomonas;* s__*Pseudomonas_oryzihabitans* | 3.257532 | CRAB | 3.031619 | 0.540291 | 0 | 0 | 0 | 0 | 0 | 0.003173 | 0 | 0 | 0 | 0 | 0 |
| k__*Bacteria;* p__*Proteobacteria;* c__*Gammaproteobacteria;* o__*Pseudomonadales;* f__*Pseudomonadaceae;* g__*Pseudomonas;* s__*Pseudomonas_putida* | 3.102421 | CRAB | 2.870397 | 0.540291 | 0 | 0.000717 | 0 | 0 | 0 | 0 | 0 | 0 | 0 | 0 | 0 |
| k__*Bacteria;* p__*Proteobacteria;* c__*Gammaproteobacteria;* o__*Pseudomonadales;* f__*Spongiibacteraceae;* g__*unidentified_Spongiibacteraceae;* s__*metagenome* | 2.235152 | CRAB | 1.967697 | 0.540291 | 0 | 0 | 0 | 0 | 0 | 0 | 0.000726 | 0 | 0 | 0 | 0 |
| k__*Bacteria;* p__*Proteobacteria;* c__*Gammaproteobacteria;* o__*Xanthomonadales;* f__*Xanthomonadaceae;* g__*Stenotrophomonas;* s__*Stenotrophomonas_maltophilia* | 3.021871 | CRAB | 2.768818 | 0.363722 | 0 | 0.000312 | 0 | 0 | 0 | 0 | 0.002117 | 0 | 0 | 0 | 0 |
| k__*Bacteria;* p__*Verrucomicrobiota;* c__*Verrucomicrobiae;* o__*Methylacidiphilales;* f__*Methylacidiphilaceae;* g__*unidentified_Methylacidiphilaceae;* s__*metagenome* | 3.203091 | CRAB | 2.943225 | 0.540291 | 0 | 0.000904 | 0 | 0 | 0 | 0 | 0 | 0 | 0 | 0 | 0 |
| *Note: LDA, Linear Discriminant Analysis; CRAB, Carbapenem-resistant Acinetobacter baumannii; CREC, Carbapenem-resistant Escherichia coli.* | | | | | | | | | | | | | | | |
