# Supplementary material for: Integrated microbiomics and metabolomics analysis reveals distinct profiles in carbapenem-resistant Acinetobacter baumannii and Escherichia coli infections in Pancreatitis-associated sepsis
Source: PLoS One. 2026 Feb 10;21(2):e0340895. doi: 10.1371/journal.pone.0340895 (PMC12890157; doi:10.1371/journal.pone.0340895)
Supplement: S4 Table — (DOCX) [file pone.0340895.s009.docx]

S4 Table. Internal standard information.

| Mode | Index | Q1 (Da) | RT (min) | CV |
| --- | --- | --- | --- | --- |
| Pos | MWS2742 | 377.2984 | 6.08 | 0.0122 |
|  | MWS4243 | 200.047 | 2.5 | 0.0158 |
|  | MWS042381 | 292.2347 | 6.13 | 0.0224 |
|  | MWS1055 | 128.0745 | 4.42 | 0.0241 |
| Neg | MWS1055 | 126.0616 | 4.4 | 0.0362 |
|  | MWS04428 | 144.1117 | 6.31 | 0.038 |
|  | MWS04198 | 126.1461 | 5.04 | 0.0422 |
|  | MWS04187 | 208.1147 | 2.36 | 0.0457 |

Note: Q1 (Da), the molecular weight of the precursor ion after the electrospray ion source is added to the ion; RT (min), retention time; CV, coefficient of variation
