# Supplementary material for: Integrated microbiomics and metabolomics analysis reveals distinct profiles in carbapenem-resistant Acinetobacter baumannii and Escherichia coli infections in Pancreatitis-associated sepsis
Source: PLoS One. 2026 Feb 10;21(2):e0340895. doi: 10.1371/journal.pone.0340895 (PMC12890157; doi:10.1371/journal.pone.0340895)
Supplement: S5 Table — (DOCX) [file pone.0340895.s010.docx]

S5 Table. Correlation analysis between microorganisms and metabolites.

| Index | *Taxonomy* | Correlation | P-value | Compounds |
| --- | --- | --- | --- | --- |
| MW0150273 | k__*Bacteria;* p__*Proteobacteria;* c__*Alphaproteobacteria;* o__*Rhizobiales;* f__*Xanthobacteraceae* | 0.887270059 | 0.000269139 | Glutathionylaminopropylcadaverine |
| MW0162939 | k__*Bacteria;* p__*Proteobacteria;* c__*Gammaproteobacteria;* o__*Enterobacterales;* f__*Aeromonadaceae* | 0.887270059 | 0.000269139 | [3-[(9Z,12Z)-octadeca-9,12-dienoyl]oxy-2-[(9Z,12Z,15Z)-octadeca-9,12,15-trienoyl]oxypropyl] 2-(trimethylazaniumyl)ethyl phosphate |
| MW0109935 | k__*Bacteria;* p__*Firmicutes;* c__*Bacilli;* o__*unidentified_Bacilli;* f__*Staphylococcaceae* | 0.852867863 | 0.000847239 | Threoninyl-Isoleucine |
| MW0150171 | k__*Bacteria;* p__*Firmicutes;* c__*Bacilli;* o__*unidentified_Bacilli;* f__*Staphylococcaceae* | 0.852867863 | 0.000847239 | Glu-Thr-Tyr-Glu |
| MW0052505 | k__*Bacteria;* p__*Firmicutes;* c__*Bacilli;* o__*unidentified_Bacilli;* f__*Staphylococcaceae* | 0.852867863 | 0.000847239 | Ecdysone |
| MW0106181 | k__*Bacteria;* p__*Bacteroidota;* c__*Bacteroidia;* o__*Flavobacteriales;* f__*Flavobacteriaceae* | 0.831809397 | 0.001498033 | 2-tert-Pentylcyclohexyl acetate |
| MW0148946 | k__*Bacteria;* p__*Proteobacteria;* c__*Gammaproteobacteria;* o__*Enterobacterales;* f__*Aeromonadaceae* | 0.827788267 | 0.001655873 | Epicainide |
| MEDN0685 | k__*Bacteria;* p__*Proteobacteria;* c__*Alphaproteobacteria;* o__*Rhizobiales;* f__*Xanthobacteraceae* | 0.827788267 | 0.001655873 | Undecanedioic acid |
| MW0011844 | k__*Bacteria;* p__*Proteobacteria;* c__*Gammaproteobacteria;* o__*Enterobacterales;* f__*Aeromonadaceae* | 0.822831451 | 0.001867191 | Tacalcitol |
| MW0053750 | k__*Bacteria;* p__*Firmicutes;* c__*Bacilli;* o__*unidentified_Bacilli;* f__*Staphylococcaceae* | 0.821280165 | 0.001937285 | Glycochenodeoxycholic acid |
| MW0153148 | k__*Bacteria;* p__*Firmicutes;* c__*Bacilli;* o__*unidentified_Bacilli;* f__*Staphylococcaceae* | 0.821280165 | 0.001937285 | Lys-Thr-Glu-Lys |
| MW0130804 | k__*Bacteria;* p__*Bacteroidota;* c__*Bacteroidia;* o__*Flavobacteriales;* f__*Flavobacteriaceae* | 0.810750932 | 0.002466276 | 2-Hydroxy-3-(4-methoxyphenyl)propanoic acid |
| MW0141040 | k__*Bacteria;* p__*Firmicutes;* c__*Bacilli;* o__*unidentified_Bacilli;* f__*Staphylococcaceae* | 0.810750932 | 0.002466276 | Monohydroxymethoxychlor |
| MW0057021 | k__*Bacteria;* p__*Proteobacteria;* c__*Alphaproteobacteria;* o__*Rhizobiales;* f__*Xanthobacteraceae* | 0.807961003 | 0.002622862 | 1-(9Z-octadecenoyl)-2-(9Z,12Z-octadecadienoyl)-sn-glycero-3-phosphocholine |
| MW0049216 | k__*Bacteria;* p__*Proteobacteria;* c__*Alphaproteobacteria;* o__*Rhizobiales;* f__*Xanthobacteraceae* | 0.807961003 | 0.002622862 | Deoxycholic acid |
| MW0059093 | k__*Bacteria;* p__*Proteobacteria;* c__*Alphaproteobacteria;* o__*Rhizobiales;* f__*Xanthobacteraceae* | 0.803004187 | 0.00291916 | 1-(1Z-octadecenyl)-2-(9Z-octadecenoyl)-sn-glycero-3-phosphoethanolamine |
| MEDN0685 | k__*Bacteria;* p__*Bacteroidota;* c__*Bacteroidia;* o__*Flavobacteriales;* f__*Flavobacteriaceae* | 0.800221699 | 0.003096004 | Undecanedioic acid |
| MW0145889 | k__*Bacteria;* p__*Proteobacteria;* c__*Alphaproteobacteria;* o__*Rhizobiales;* f__*Xanthobacteraceae* | -0.807961003 | 0.002622862 | Asn-Ser-Lys-Ile-Val |
| MW0148580 | k__*Bacteria;* p__*Proteobacteria;* c__*Gammaproteobacteria;* o__*Enterobacterales;* f__*Aeromonadaceae* | -0.812917819 | 0.002349546 | Diospyrin |
| MW0141373 | k__*Bacteria;* p__*Proteobacteria;* c__*Gammaproteobacteria;* o__*Enterobacterales;* f__*Aeromonadaceae* | -0.812917819 | 0.002349546 | 13-(beta-D-glucosyloxy)docosanoic acid |
| MW0007374 | k__*Bacteria;* p__*Proteobacteria;* c__*Gammaproteobacteria;* o__*Enterobacterales;* f__*Aeromonadaceae* | -0.812917819 | 0.002349546 | Indapamide |
| MEDL02748 | k__*Bacteria;* p__*Proteobacteria;* c__*Gammaproteobacteria;* o__*Enterobacterales;* f__*Aeromonadaceae* | -0.812917819 | 0.002349546 | Auraptene |
| MW0145216 | k__*Bacteria;* p__*Proteobacteria;* c__*Gammaproteobacteria;* o__*Enterobacterales;* f__*Aeromonadaceae* | -0.812917819 | 0.002349546 | Aquayamycin |
| MEDL02002 | k__*Bacteria;* p__*Proteobacteria;* c__*Gammaproteobacteria;* o__*Enterobacterales;* f__*Aeromonadaceae* | -0.812917819 | 0.002349546 | Erucic acid |
| MEDN1270 | k__*Bacteria;* p__*Firmicutes;* c__*Bacilli;* o__*unidentified_Bacilli;* f__*Staphylococcaceae* | -0.821280165 | 0.001937285 | LPE(22:6/0:0) |
| MW0114723 | k__*Bacteria;* p__*Proteobacteria;* c__*Gammaproteobacteria;* o__*Enterobacterales;* f__*Aeromonadaceae* | -0.822831451 | 0.001867191 | Leucomycin A1 |
| MW0055397 | k__*Bacteria;* p__*Proteobacteria;* c__*Gammaproteobacteria;* o__*Enterobacterales;* f__*Aeromonadaceae* | -0.822831451 | 0.001867191 | Notoginsenoside T1 |
| MW0150931 | k__*Bacteria;* p__*Proteobacteria;* c__*Gammaproteobacteria;* o__*Enterobacterales;* f__*Aeromonadaceae* | -0.827788267 | 0.001655873 | His-Glu-Tyr-Lys |
| MW0054544 | k__*Bacteria;* p__*Proteobacteria;* c__*Gammaproteobacteria;* o__*Enterobacterales;* f__*Aeromonadaceae* | -0.832745083 | 0.001462982 | 1-Arachidonoyl-sn-glycero-3-phosphocholine |
| MW0144032 | k__*Bacteria;* p__*Proteobacteria;* c__*Gammaproteobacteria;* o__*Enterobacterales;* f__*Aeromonadaceae* | -0.832745083 | 0.001462982 | 8-Bromoadenosine |
| MEDP1836 | k__*Bacteria;* p__*Proteobacteria;* c__*Gammaproteobacteria;* o__*Enterobacterales;* f__*Aeromonadaceae* | -0.832745083 | 0.001462982 | Nootkatone |
| MW0105634 | k__*Bacteria;* p__*Proteobacteria;* c__*Gammaproteobacteria;* o__*Enterobacterales;* f__*Aeromonadaceae* | -0.837701899 | 0.001287441 | Antanapeptin C |
| MW0016270 | k__*Bacteria;* p__*Proteobacteria;* c__*Gammaproteobacteria;* o__*Enterobacterales;* f__*Aeromonadaceae* | -0.847615531 | 0.000984231 | Butyric acid |
| MW0054252 | k__*Bacteria;* p__*Proteobacteria;* c__*Gammaproteobacteria;* o__*Enterobacterales;* f__*Aeromonadaceae* | -0.867442795 | 0.000541623 | LacCer(d18:1/16:0) |
| MW0063299 | k__*Bacteria;* p__*Proteobacteria;* c__*Gammaproteobacteria;* o__*Enterobacterales;* f__*Aeromonadaceae* | -0.877356427 | 0.000387485 | R-2 Methanandamide |
| MW0150843 | k__*Bacteria;* p__*Proteobacteria;* c__*Gammaproteobacteria;* o__*Enterobacterales;* f__*Aeromonadaceae* | -0.877356427 | 0.000387485 | His-Arg-Val |
| MW0155388 | k__*Bacteria;* p__*Proteobacteria;* c__*Gammaproteobacteria;* o__*Enterobacterales;* f__*Aeromonadaceae* | -0.877356427 | 0.000387485 | Phe-Tyr-Lys-Arg |
| MW0055403 | k__*Bacteria;* p__*Proteobacteria;* c__*Gammaproteobacteria;* o__*Enterobacterales;* f__*Aeromonadaceae* | -0.887270059 | 0.000269139 | N-Palmitoylsphingosine |
| *Note: CRAB, Carbapenem-resistant Acinetobacter baumannii; CREC, Carbapenem-resistant Escherichia coli.* | | | | |
